# Identification and Characterization of the CRISPR/Cas System in *Staphylococcus aureus* Strains From Diverse Sources

**DOI:** 10.3389/fmicb.2021.656996

**Published:** 2021-06-02

**Authors:** Erick Adrian Cruz-López, Gildardo Rivera, María Antonia Cruz-Hernández, Ana Verónica Martínez-Vázquez, Graciela Castro-Escarpulli, Rebeca Flores-Magallón, Karina Vázquez, Wendy Lizeth Cruz-Pulido, Virgilio Bocanegra-García

**Affiliations:** ^1^Laboratorio Interacción Ambiente-Microorganismo, Centro de Biotecnología Genómica, Instituto Politécnico Nacional, Tamaulipas, Mexico; ^2^Laboratorio de Investigación Clínica y Ambiental, Departamento de Microbiología, Escuela Nacional de Ciencias Biológicas, Instituto Politécnico Nacional, Mexico City, Mexico; ^3^Centro Interdisciplinario de Investigación Para el Desarrollo Integral Regional, Unidad Michoacán, Instituto Politécnico Nacional, Jiquilpan, Mexico; ^4^Facultad de Medicina Veterinaria, Universidad Autónoma de Nuevo León, San Nicolás de los Garza, Mexico; ^5^Research Department, Universidad del Valle de México, Campus Reynosa, Mexico City, Mexico

**Keywords:** *Staphylococcus aureus*, methicillin-resistant *Staphylococcus aureus*, multidrug resistant, CRISPR-Cas system, phage therapy

## Abstract

The CRISPR-Cas [clustered regularly interspaced short palindromic repeats and the CRISPR-associated genes (Cas)] system provides defense mechanisms in bacteria and archaea vs. mobile genetic elements (MGEs), such as plasmids and bacteriophages, which can either be harmful or add sequences that can provide virulence or antibiotic resistance. *Staphylococcus aureus* is a Gram-positive bacterium that could be the etiological agent of important soft tissue infections that can lead to bacteremia and sepsis. The role of the CRISPR-Cas system in *S. aureus* is not completely understood since there is a lack of knowledge about it. We analyzed 716 genomes and 1 genomic island from GENOMES-NCBI and ENA-EMBL searching for the CRISPR-Cas systems and their spacer sequences (SSs). Our bioinformatic analysis shows that only 0.83% (6/716) of the analyzed genomes harbored the CRISPR-Cas system, all of them were subtype III-A, which is characterized by the presence of the *cas10/csm1* gene. Analysis of SSs showed that 91% (40/44) had no match to annotated MGEs and 9% of SSs corresponded to plasmids and bacteriophages, indicating that those phages had infected those *S. aureus* strains. Some of those phages have been proposed as an alternative therapy in biofilm-forming or infection with *S. aureus* strains, but these findings indicate that such antibiotic phage strategy would be ineffective. More research about the CRISPR/Cas system is necessary for a bigger number of *S. aureus* strains from different sources, so additional features can be studied.

## Introduction

The bacteria and archaea have developed defense mechanisms against bacteriophages (Labrie et al., 2010), in the form of restriction and modification system (R-M *system*; Huff et al., 2017) and as the CRISPR-Cas [clustered regularly interspaced short palindromic repeats and the CRISPR-associated genes (Cas)] system, both of which degrade the foreign invader genetic material. The CRISPR-Cas is a natural, memory, and hereditary mechanism that protects bacteria against bacteriophages (Faruque et al., 2005; Box et al., 2015; Hille et al., 2018). It is composed of (1) a group of genes *cas*, (2) a locus or loci, CRISPR formed by spacer sequences (SSs) separated into repeated sequences (SRs), and (3) the leader sequence placed upstream from locus CRISPR (Westra et al., 2014); the set of *cas* genes is divided into the module of adaptation formed by *cas1* and *cas2* genes and the effector complex where the rest of the *cas* genes are placed (Koonin et al., 2017).

The system CRISPR-Cas current classification includes 2 classes, 6 types, and 33 subtypes. Class 1 systems use multi-unit protein complexes (Koonin and Makarova, 2017; Koonin et al., 2017) and Class 2 systems use only one multidomain protein (Shmakov et al., 2017) for the degradation of the genetic material. This DNA degradation occurs in three stages (Hsu et al., 2014): (1) adaptation stage during a primo-infection (Nuñez et al., 2014), (2) expression stage during reinfection, and (3) interference stage for the digestion mobile genetic element (MGE) through endonucleases Cas, which are guided by crRNA (chimera of SE and SR; Hille et al., 2018). In a MGE, there are short sequences (approximately 30 nucleotides) marked by protospacer adjacent motifs (Jiang and Doudna, 2015, 2017), known as protospacers, which are inserted like a SS.

The CRISPR-Cas system has been detected in Gram-positive bacteria, such as *Lactobacillus* spp. (Wang et al., 2020; Yang et al., 2020) and pathogenic bacteria, such as *Enterococcus* spp. (Sanderson et al., 2020). However, in *Staphylococcus aureus*, it has only been detected in few strains. *S. aureus* is a Gram-positive bacterium that colonizes 30% of the population in an asymptomatic way, and also it is the etiological agent of several important infections (Craft et al., 2019). In 1960, the methicillin-resistant *S. aureus* (MRSA) strains were detected (Chambers and Deleo, 2009), and those are still a current cause of soft tissue infections. New effective antibiotic therapies are a current demand (Yang et al., 2018; Labruère et al., 2019). The application of bacteriophages as a therapy to treat *S. aureus* infections (Kaźmierczak et al., 2014) is a promising alternative. The memory capacity of the CRISPR-Cas system allows us to understand the dynamic between an MGE and its hosts (bacteria and archaea). The sequenced bacterial genomes are the current data source for searching CRISPR-Cas system in important medical bacteria such as *S. aureus*. Despite studies searching CRISPR-Cas system in *S. aureus* (Cao et al., 2016; Zhao et al., 2018; Rossi et al., 2019), further research is needed to study this system in more *S. aureus* strains to understand the effects and its association to pathogenicity. Thus, the aim of this study was to search CRISPR-Cas in *S. aureus* genomes and its characterization *via* bioinformatic tools, as well as the association of the SS with MGEs.

## Materials And Methods

### Genomes Collection

The complete *S. aureus* genomes were downloaded from GENOME-NCBI [*n* = 864 (484 chromosomes and 380 plasmids)] and ENA-EMBL [*n* = 521 (232 chromosomes, 288 plasmids, and 1 pathogenicity island)]. In total, there were 716 strains used.

### CRISPR-Cas System Determination

The genomes were analyzed using CRISPRCasFinder 4.2.2 (Grissa et al., 2007; Abby et al., 2014; Couvin et al., 2018). The server was used with default parameters: minimal repeat length 23 bp, maximal repeat length 55 bp, repeat mismatch, minimal spacer size in function of repeat size 0.6, maximal spacer size in function of repeat size 2.5, maximally allowed percentage of similarity between spacers 60, percentage mismatches allowed between repeats 20, percentage mismatches allowed for truncated repeat 33. Also, a default 100 bp size of flanking regions in all potential CRISPR arrays was included. A CRISPR-Cas system that presents a group of genes *cas* and the locus CRISPR with a score of 3 and 4 were considered for the next analysis.

### Cas1, Cas2, Cas6, and Cas10 Phylogenetic Analysis Proteins

The files containing the coding sequences of each CRISPR-Cas system-bearing genome were downloaded from GENOME-NCBI. The *cas* genes were obtained from those files and translated into MEGA-X by using the standard genetic code. Later, the Cas proteins were aligned to the program Clustal O of Unipro UGENE. The scores “pair sum” were calculated in GeneDoc. The best alignments showed lower scores. Subsequently, the phylogenetic trees were built by the UPGMA method using default parameters with 1,000 bootstrap in the program MEGA-X (Kumar et al., 2018).

### Cas Protein Analysis

The Cas sequences annotated images were generated in the program EasyFig 2.2.5 (Sullivan et al., 2011).

### Phylogenetic Analysis of the Repeated Sequences

The analysis was the same process as the Cas proteins, except that we used the neighbor-joining method with default parameters to build the phylogenetic tree, using MEGAX (Rose et al., 2019).

### Secondary Structures of Repeat Sequence Consensus

The secondary structures of repeat sequence consensus (SRc) and the minimum free energy (MFE) were obtained from the RNAfold web server (http://rna.tbi.univie.ac.at//cgi-bin/RNAWebSuite/RNAfold.cgi; Lorenz et al., 2011). The logo of SRc was obtained from WebLogo (Schneider and Stephens, 1990; Crooks et al., 2004).

### Spacer Sequence Analysis

The FASTA files were downloaded from CRISPR-CasFinder. Spacer sequence (SE) were submitted to BLAST, and the results associated with the MGE were the ones considered with expected values (*e*-values) minor or equal to 0.0001 and scores above 40 (Ostria-Hernández et al., 2015). Next, a 0 and 1 matrix was developed, 1 being the cell where the MGE and *S. aureus* strain intercept. That matrix was analyzed in the ClustVis server using default parameters. Then, a heat map was elaborated with the webserver ClustVis (Metsalu and Vilo, 2015), where the MGE known as the aforementioned infected strain of *S. aureus* was appreciated.

## Results

**Figure 1:**
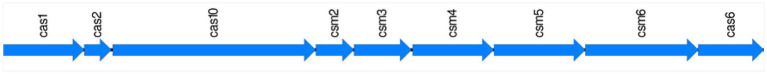
The cluster of genes *cas* of the CRISPR-Cas subtype III-A of *S. aureus* system. The annotation genes *cas* was done in EasyFig 2.2.5.

The CRISPR-Cas system was searched in 1,385 sequences of *S. aureus*, including chromosomes, plasmids, and 1 pathogenicity island, collected from 2 databases (Supplementary Table A). The search showed that 0.83% (6/716) of *S. aureus* strains harbored the CRISPR-Cas system. The strains harboring the CRISPR-Cas system were *S. aureus* 08BA02176 (NC_018608), *S. aureus* KUH140087 (NZ_AP020315), *S. aureus* JS395 (NZ_CP012756), *S. aureus* AR_0470 (NZ_CP029653), *S. aureus* AR_0472 (NZ_CP029649), and *S. aureus* AR_0473 (NZ_CP029681). All these strains have different geographical origin: *S. aureus* 08BA02176 was isolated from a surgery infection in 2008 from a Canadian patient (Golding et al., 2012); *S. aureus* KUH140087 was isolated in 2014 from a septicemia patient in Kyoto, Japan (Hikichi et al., 2019); *S. aureus* JS395 was isolated in 2008 from a patient in Switzerland (Larsen et al., 2017), and the *S. aureus* strains AR_0472, AR_0470, and AR_0473 were sent by the Center for Disease Control and Prevention. While the source of *S. aureus* AR_0472, AR_0470, and AR_0473 is uncertain, the rest of the strains come from clinic sources. All the detected CRISPR-Cas systems were found in chromosome structures, and none were detected in the pathogenicity island; nevertheless, other islands had it (Chakraborty et al., 2009; Carpenter et al., 2017). The detected systems found belong to the III-A subtype, which is characterized by the gen *cas10/csm1* and *cas* genes ordered as shown in Figure 1 (Koonin et al., 2017). The detected CRISPR-Cas system structure was as follows: (1) *cas* genes nearby the locus CRISPR and (2) scores of 3 and 4 in the CRISRPRCasFinder scale (Pourcel et al., 2020; Supplementary Table B). The strain contains a group of *cas* genes (Figure 1) near the locus CRISPR-Cas; the CRISPR locus and the cluster *cas* are separated by 73 nt (strains JS395 and AR_0470), 74 nt (strains 08BA02176, AR_0472, and AR_0473), and 133 nt (strain KUH140087). Each strain with CRISPR-Cas has a unique locus CRISPR with a different number of SS.

**Figure 2:**
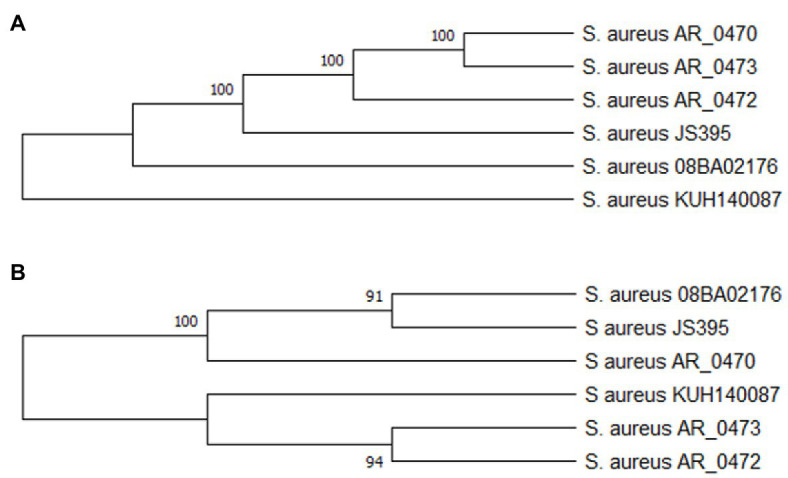
Protein Cas phylogenetic relation **(A)** and SRc **(B)**. The alignment of the amino acid sequences of the proteins Cas and SRc was done with ClustalO, and the phylogenetic trees of SRc and the Cas proteins were built with the neighbor-joining and UPGMA method. There is a tree for the protein Cas (Cas1, Cas2, Cas10/Csm1, and Cas6) because they presented the same phylogenetic relation. The trees are the consensus of 1,000 bootstrap, and they were done with MEGA X.

The SRc was the same in two strains (i.e., AR_0472 and AR_0473) and different in four strains. The SRc length was 36 and 37 nt; the SRc formed by 37 nt is shown in the strains JS395 and AR_0479. The SRc remains nucleotide motifs (Figure 2) that stand out among the conservative nucleotides: four consecutive nucleotides of cysteine (-CCCC-) and four consecutive nucleotides of guanine (-GGGG-). Among the conserved motifs, there is a constant region of eight nucleotides.

**Figure 3:**
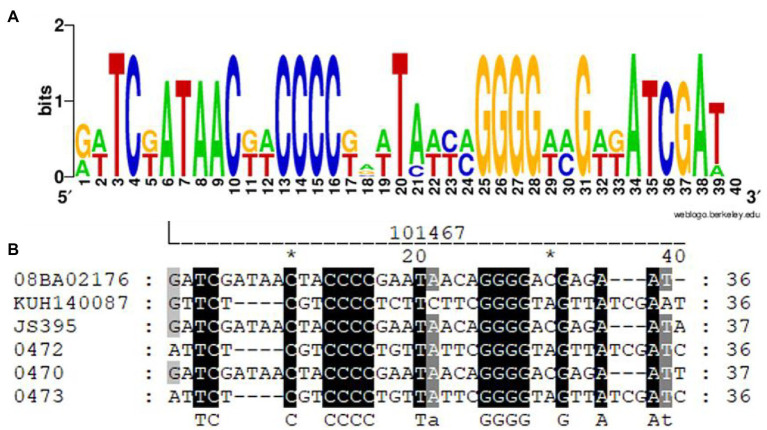
SRc alignment of the CRISPR-Cas system present in *S. aureus*. Visualization **(A)** and alignment of nucleotides **(B)**. The motive nucleotides are under the alignment (capital letters). The alignment was done with MUSCLE (UGENE), and the image was obtained from WebLogo **(A)** and GeneDoc **(B)**.

The Cas proteins and SR keep a function–structure relationship (*stem-loop* structure); the coevolution of both structures is necessary for the correct function of the system CRISPR-Cas. Figures 3A,B show the phylogenetic relations of the Cas proteins and SRc, respectively. The Cas proteins and SRc present in *S. aureus* KUH140087 are phylogenetically away from the ones present in the phylogroup formed by the rest of the strains.

**Figure 4:**
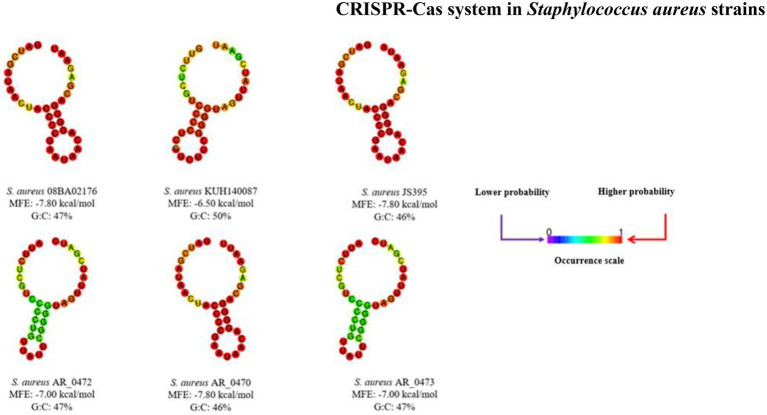
Secondary structures of repeated sequences. Each secondary structure is the result of the interactions of the nucleotides; these structures and the minimum free energy were obtained in the RNAfold server. It shows the scale of occurrence for each nucleotide interaction.

The SR keeps the nucleotides that form the *stem-loop* structure, which gives the signals of the location where the cuts must be done on pre-crRNA. Figure 4 shows the SRc secondary structures of the CRISPR-Cas systems found; the *stem* formed by interactions G:C (guanine:cysteine) can be seen, and the *loop* also indicates the MFE of each structure.

The memory and adaptation characteristics of the CRISPR-Cas system allow the bacteria to identify which MGE infected it. The subtraction of the protospacer from the MGE and its incorporation as SS in the locus CRISPR during the adaptation phase (McGinn and Marraffini, 2019) becomes an advantage in the genomic analysis. The total of SS [6 SS (strain JS395), 12 SS (strain AR_0470, AR_0472, and AR_0473), and 15 SS (strain 08BA02176)] is 62, where 26 (41.93%) are unique SS and 18 are SS duplicated and built 58.07%. Interestingly, the duplicated SSs are in the loci of strains AR_0472, AR_0473, JS395, and AR_0470. The SSs are preserved between the loci CRISPR: the repeated SSs of the strain AR_0472 (*n* = 12) match in order and sequence with the SS of the strain AR_0473 (*n* = 12), and the loci CRISPR of the strain JS395 (*n* = 6) also matches in order and sequence with a final 50% of loci CRISPR of strain AR_0470 (*n* = 12). Hence, only 44 SSs were considered for BLAST analysis.

**Figure 5:**
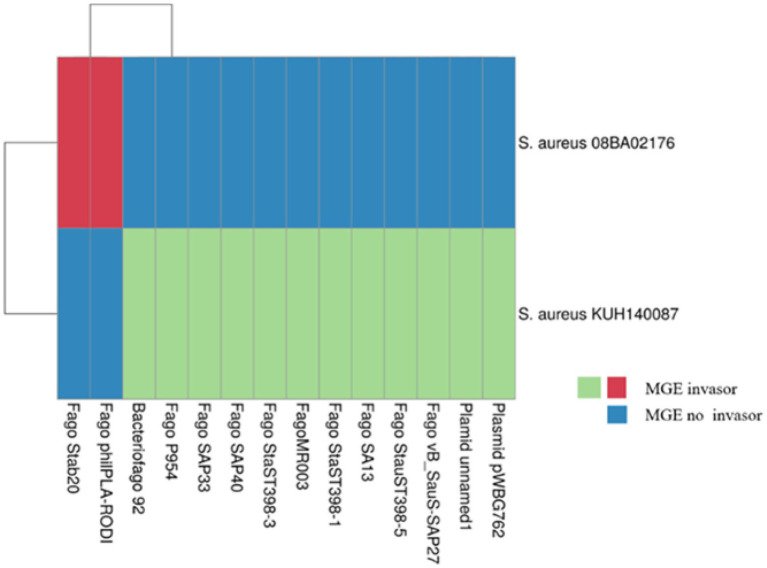
CRISPR-Cas ported strains that exhibited information of known mobile genetic elements (MGE). The annotations of the heat map were appended. The strains shown are unique, in which some of the spacer sequences were associated with an MGE through BLAST. The map was obtained in ClusVis.

The BLAST analysis showed that 9% (4/44) of SSs match with known MGE (Supplementary Table C). In Figure 5, MGEs that infected the *S. aureus* 08BA02176 (SS6) and *S. aureus* KUH140087 (SS1, SS2, and SS3) are presented. However, if a SS is associated with more than one MGE, it means that the protospacers are conserved between MGEs. Besides, according to our analysis, a specific protospacer can be found in plasmids or bacteriophages, but a plasmid protospacer is not found in a bacteriophage and vice versa. The SS1 of *S. aureus* KUH140087 is the only one that interferes with the two plasmids named in Figure 5, and the rest of SS interferes with the bacteriophage.

## Discussion

The CRISPR-Cas system is a heritable mechanism of immunity in bacteria and archaea, which protects them from foreign plasmids and bacteriophages; it is an endonuclease mechanism guided by crRNA (Makarova et al., 2013). Few studies have searched for the CRISPR-Cas system in *Staphylococcus* spp., where the CRISPR-Cas system was found in 0.94% (6/616) of isolated clinics (Cao et al., 2016) and in 7.89% (3/39) of the *S. aureus* strains analyzed for Zhao et al. (2018); moreover, the CRISPR-Cas system was searched in 129 isolated from *Staphylococcus* spp. (*S. aureus n* = 53, *Staphylococcus pseudintermedius n* = 74, *Staphylococcus haemolyticus n* = 1, and *Staphylococcus cohnii n* = 1) from 9 countries, and the 8% (10/129) are CRISPR-Cas system-bearing strains, but it was detected only in *S. pseudintermedius* strains (Rossi et al., 2019). Few studies have searched the CRISPR-Cas system in MGE, such as plasmids (Kamruzzaman and Iredell, 2020) or bacteriophages (Naser et al., 2017). The existence of the CRISPR-Cas system in a minimalist form, inactive, partially active, or active in MGE is the result of the constant coevolution between microorganisms and MGE (Faure et al., 2019), or due to competency between plasmids as a plasmid incompatibility mechanism (Kamruzzaman and Iredell, 2020).

In this study, the CRISPR-Cas system was found in six *S. aureus* strains. Interestingly, the strains were isolated from different countries: *S. aureus* 08BA02176 in Canada (Golding et al., 2012), *S. aureus* KUH140087 in Kyoto, Japan (Hikichi et al., 2019), *S. aureus* JS395 in Switzerland (Larsen et al., 2017), and the *S. aureus* AR_0472, AR_0470, and AR_0473 strains, whose geographical origin is unknown. The few CRISPR-Cas-bearing strains and their different geographical origin led us to think that the CRISPR-Cas system in *S. aureus* might be a spontaneous biological phenomenon, which means that the CRISPR-Cas system found in this study might be part of a bacterium that lives together with *S. aureus*. It has been demonstrated that in *S. aureus* 08BA02176, *S. schleiferi* TSCC54, and *S. capitis* CR01, the CRISPR-Cas system is inside the staphylococcal chromosomic cassette (*SSC*) *SSCmec*. The *SSCmec* is flanked by insertion sequences (IS), in *S. aureus* 08BA02176 by IS6 and ISL3, in *S. schleiferi* TSCC54 by IS6 and IS1182, and in *S. capitis* CR01 by an IS6, and the presence of the MGE mentioned indicates that the CRISPR-Cas system has been transferred horizontally to other strains and species of *Staphylococcus* (Rossi et al., 2017). The results of this study support the proposal of Rossi et al. (2017) and allow us to postulate that the CRISPR-Cas in *S. aureus* might be a spontaneous event consequence of a horizontal transfer of the *SCCmec* because of the low number of strains harboring the CRISPR-Cas system and their different geographical regions. Further evidence of horizontal transfer of the CRISPR-Cas system through *SCCmec* requires additional bioinformatic analysis and its *in vitro* demonstration.

The CRISPR-Cas systems in the *S. aureus* strains analyzed in this study are classified as subtype III-A, since the gen *cas10/csm1* is found (Koonin et al., 2017). Studies have been demonstrated that the HD dominion of the protein Cas10/Csm1 is responsible for the activity ssDNasa and the protein Csm3 of the activity endoribonucleases (Tamulaitis et al., 2017). The crRNA is essential for the operation of the CRISPR-Cas system (Behler and Hess, 2020). Figures 3A,B show that the Cas protein and the SRc coevolution comply with the correct functioning of the CRISPR-Cas system and that the *stem-loop* structure is conserved; in the alignment (Figure 2B) of the SRc, it has been demonstrated that the presence of conserved motifs is formed by four cysteines and four guanines that flanked an inner region of eight nucleotides. Motifs rich C and G can interact to form a pair of C:G, which has also been observed in *Proteus* spp. (Qu et al., 2019). The alignments of SRc evidence the conserved motifs (Yang et al., 2020) that interact to generate secondary structures (Figure 4), which are characterized by the *stem-loop* structure (Bhaya et al., 2011) that serves as a point to process the pre-crRNA through the endonuclease Cas6 (Wakefield et al., 2015). The secondary structure stability is bigger as far as there are more G:C interactions and less MFE (Trotta, 2014); nevertheless, the nucleotides bound in different forms to G:C, so there are also stable structures (Yang et al., 2015; Negahdaripour et al., 2017).

Multidrug-resistant (MDR) strains arise because exposition to antimicrobial compound (AMC) in the environment selects them (Vuotto et al., 2018; Sanderson et al., 2020), as well as horizontal AMC gene transfer (Zarei-Baygi et al., 2019) through MGE (Baker et al., 2018). This relation between ARG and MGE is difficult for the therapy of MDR bacterial infections (Vuotto et al., 2018). *S. aureus* strains that contain the CRISPR-Cas system are detected in this study, three are from a clinical origin (08BA02176, JS395, and KUH140087), and the origin of the rest (AR_0470, AR_0472, and AR_0473) is unknown. The presence of antimicrobial resistance (AMR) in the environment may be the result of its incorrect use, for instance, the livestock industry and the pig industry, where they are used for animal breeding (Zhu et al., 2013) as well as their indiscriminate use to treat infection diseases (Saha et al., 2019) or their long-lasting use in severe or chronic treatments (Karaiskos et al., 2019). The cross pollution favors the outcome of MDR to different places far from its origin (Uhlemann et al., 2017; Aeksiri et al., 2019; Cohen et al., 2019). The effort and the economic consumption to the development of antimicrobial products (Chung and Khanum, 2017; Hashemi et al., 2018), mainly those are effective against MDR strains with metal in the form of a nanoparticle (Alavi and Rai, 2019; Heidary et al., 2019; Kumar et al., 2019), have promoted the search of new treatments, particularly the treatment of bacteriophages (Wernicki et al., 2017). The bacteriophages are being considered as an alternative to therapy in *S. aureus* MDR strains (MRSA), *S. haemolyticus* (MRSH), and *Staphylococcus epidermidis* (MRSE) infections (Oduor et al., 2020). However, in this study, we found that the CRISPR-Cas system may be a factor that could compromise the efficacy of bacteriophage therapy. The BLAST analysis of SE6 has shown that *S. aureus* 08BA02176 is capable of counteracting the Stab20 bacteriophages infection. Oduor et al. (2019) isolated the Stab20, Stab21, Stab22, and Stab23 bacteriophages, and later it was determined that Stab20 and Stab21 infected 41 and 40, among them, 100 *Staphylococcus* spp. (MRSA, MSSA, *Staphylococcus intermedius*, *S. epidermidis*, *Staphylococcus saprophyticus*, and *S. haemolyticus*) strains; moreover, it was found that Stab20 and Stab21 are better spread in some *S. aureus* strains. The Stab21 bacteriophage is capable to infect an isolated *S. aureus* from a patient with chronic sinusitis (Oduor et al., 2020). The presence of one SE that matches with Stab20 in the loci CRISPR of the 08BA02176 strain implies that infection with this strain would be difficult, or impossible to treat with a Stab20 bacteriophage therapy. Likewise, *S. aureus* 08BA02176 strain demonstrated its capability to destroy the ɸIPLA-RODI phage; this phage, when used against *S. aureus* forming a biofilm, caused a reduction of the population of *S. aureus* after 18 h (González et al., 2017); nevertheless, the presence of *S. aureus* 08BA02176 as part of the biofilm makes the use of the ɸIPLA-RODI phage difficult as a treatment. In contrast, it was demonstrated that the ɸMR003 phage infected 97% of the MRSA strains in the study of Peng et al. (2019); however, the CRISPR-Cas system of *S. aureus* KUH140087 prevents attack by the ɸMR003 phage.

Despite the ongoing protocols using the bacteriophages to treat infections caused by *S. aureus* (Kaźmierczak et al., 2014; Cui et al., 2017) in an animal model and human studies, it is necessary to generate more knowledge about the CRISPR-Cas system in more *S. aureus* strains to develop reliable bacteriophage therapies. Nowadays, only 12 *S. aureus* strains contain the reliable CRISPR-Cas system: AH1, AH2, AH3, SH1, SH2, and SH3 strains from isolated clinics (Cao et al., 2016), and the 08BA02176, KUH140087, JS395, AR_0470, AR_0472, and AR_0473 strains found in this investigation, as well as the ones previously found in the study by Cao et al. (2016) are 08BA02176 and JS395 strains as the CRISPR-Cas system carrier.

In conclusion, we determined that the CRISPR-Cas system found has an origin from other bacteria before getting into the different *S. aureus* strains detected in this study, due to its rare presence in clinical infections and its wide geographical countries where the CRISPR-Cas system was detected; moreover, the CRISPR-Cas system-bearing bacteria can destroy the bacteriophages becoming the limiting factor that could avoid the therapeutic use of the bacteriophages. Our results can be complemented with the CRISPR-Cas system detection in more *S. aureus* strains; thus, the enrichment of the database is to associate the memory of the CRISPR-Cas system with the bacteriophages and to discriminate among the best candidates for the curative therapies.

## Data Availability Statement

The datasets presented in this study can be found in online repositories. The names of the repository/repositories and accession number(s) can be found in the article/Supplementary Material.

## Author Contributions

EC-L and VB-G: design of the work. EC-L, MC-H, AM-V, and GC-E: acquisition and analysis of the data. EC-L, GR, WC-P: writing and revision of the content. EC-L, RF-M, and KV: writing of the content and contribution to figures. VB-G: approval of the last version. All authors contributed to the article and approved the submitted version.

### Conflict of Interest

The authors declare that the research was conducted in the absence of any commercial or financial relationships that could be construed as a potential conflict of interest.
