# Supplementary material for: Identification and Characterization of the CRISPR/Cas System in Staphylococcus aureus Strains From Diverse Sources
Source: Front Microbiol. 2021 Jun 2;12:656996. doi: 10.3389/fmicb.2021.656996 (PMC8206494; doi:10.3389/fmicb.2021.656996)
Supplement: Supplementary file 1 [file Table_1.DOCX]

Supplementary table A. Table of genomes of *Staphylococcus aureus* that bear and no-bear the CRISPR-Cas system.

This supplementary table content all CRISPR-Cas system- and CRISPR-Cas system non- bearing genomes. It is shown at first column the accession number of each genome, at the second column is shown the description of each sequence, at the third column either subtype or absence of the CRISPR-Cas system, at the fourth column the evidence level of CRISPR loci, at the fifth column either presence (what *cas* genes) or absence of cluster *cas* genes and column sixth the database come from each sequence nucleotide.

| **Supplementary table A.** **Table of CRISPR-Cas system- and CRISPR-Cas system non-bearing strains of *Staphylococcus aureus*.** | | | | | | |
| --- | --- | --- | --- | --- | --- | --- |
| **#** | **ACCESSION NUMBER** | **DESCRIPTION** | **CRISPR-Cas system** | **EVIDENCE LEVEL** | ***cas* GENES** | **DATABASE** |
| 1 | CP038461 | Strain O217 | ABS | N/A | ABS | NCBI |
| 2 | CP040801 | Strain S15 | ABS | N/A | ABS | NCBI |
| 3 | CP040802 | Strain S15 plasmid unnamed 1 | ABS | N/A | ABS | NCBI |
| 4 | NC_002745 | Subsp. *aureus* Strain N315 | ABS | N/A | ABS | NCBI |
| 5 | NC_002758 | Subsp. *aureus* Strain Mu50 | ABS | N/A | ABS | NCBI |
| 6 | NC_002774 | Subsp. *aureus* Strain Mu50 plasmid VRSAp | ABS | N/A | ABS | NCBI |
| 7 | NC_002951 | Subsp. *aureus* Strain COL | ABS | N/A | ABS | NCBI |
| 8 | NC_002952 | Subsp. *aureus* Strain MRSA252 | ABS | N/A | ABS | NCBI |
| 9 | NC_002953 | Subsp. *aureus* Strain MSSA476 | ABS | N/A | ABS | NCBI |
| 10 | NC_003140 | Subsp. *aureus* Strain N315 plasmid Pn315 | ABS | N/A | ABS | NCBI |
| 11 | NC_003923 | Subsp. *aureus* Strain MW2 | ABS | N/A | ABS | NCBI |
| 12 | NC_005951 | Subsp. *aureus* Strain MSSA476 plasmid pSAS | ABS | N/A | ABS | NCBI |
| 13 | NC_006629 | Subsp. *aureus* Strain COL plasmid pT181 | ABS | N/A | ABS | NCBI |
| 14 | NC_007622 | Strain RF122 | ABS | N/A | ABS | NCBI |
| 15 | NC_007790 | Subsp. *aureus* Strain USA300_FPR3757 plasmid pUSA01 | ABS | N/A | ABS | NCBI |
| 16 | NC_007791 | Subsp. *aureus* Strain USA300_FPR3757 plasmid pUSA02 | ABS | N/A | ABS | NCBI |
| 17 | NC_007792 | Subsp. *aureus* Strain USA300_FPR3757 plasmid pUSA03 | ABS | N/A | ABS | NCBI |
| 18 | NC_007793 | Subsp. *aureus* Strain USA300_FPR3757 | ABS | N/A | ABS | NCBI |
| 19 | NC_007795 | Subsp. *aureus* Strain NCTC_8325 | ABS | N/A | ABS | NCBI |
| 20 | NC_009477 | Subsp. *aureus* Strain JH9 plasmid pSJH901 | ABS | N/A | ABS | NCBI |
| 21 | NC_009487 | Subsp. *aureus* Strain JH9 | ABS | N/A | ABS | NCBI |
| 22 | NC_009619 | Subsp. *aureus* Strain JH1 plasmid pSJH101 | ABS | N/A | ABS | NCBI |
| 23 | NC_009632 | Subsp. *aureus* Strain JH1 | ABS | N/A | ABS | NCBI |
| N/A: No applique, ABS: Absent, NCBI: National Center for Biotechnology Information, ENA: European Nucleotide Archive, *Evidence level 4 and 3, *cas* genes | | | | | | |
| **Supplementary table A. Table of CRISPR-Cas system- and CRISPR-Cas system non-bearing strains of *Staphylococcus aureus* (continuation).** | | | | | | |
| **#** | **ACCESSION NUMBER** | **DESCRIPTION** | **CRISPR-Cas system** | **EVIDENCE LEVEL** | ***cas* GENES** | **DATABASE** |
| 24 | NC_009641 | Subsp. *aureus* Strain Newman | ABS | N/A | ABS | NCBI |
| 25 | NC_009782 | Subsp. *aureus* Strain Mu3 | ABS | N/A | ABS | NCBI |
| 26 | NC_010063 | Subsp. *aureus* Strain USA300_TCH1516 plasmid pUSA300houmr | ABS | N/A | ABS | NCBI |
| 27 | NC_010079 | Subsp. *aureus* Strain USA300_TCH1516 | ABS | N/A | ABS | NCBI |
| 28 | NC_012417 | Subsp. *aureus* Strain USA300_TCH1516 plasmid pUSA01-HOU | ABS | N/A | ABS | NCBI |
| 29 | NC_013450 | Subsp. *aureus* Strain ED98 | ABS | N/A | ABS | NCBI |
| 30 | NC_013451 | Subsp. *aureus* Strain ED98 plasmid pAVY | ABS | N/A | ABS | NCBI |
| 31 | NC_013452 | Subsp. *aureus* Strain ED98 plasmid pT181 | ABS | N/A | ABS | NCBI |
| 32 | NC_013453 | Subsp. *aureus* Strain ED98 plasmid Pavx | ABS | N/A | ABS | NCBI |
| 33 | NC_016912 | Subsp. *aureus* Strain VC40 | ABS | N/A | ABS | NCBI |
| 34 | NC_016928 | Subsp. *aureus* Strain M013 | ABS | N/A | ABS | NCBI |
| 35 | NC_017331 | Subsp. *aureus* Strain TW20 | ABS | N/A | ABS | NCBI |
| 36 | NC_017332 | Subsp. *aureus* Strain TW20 plasmid pTW20_2 | ABS | N/A | ABS | NCBI |
| 37 | NC_017333 | Subsp. *aureus* Strain ST398 | ABS | N/A | ABS | NCBI |
| 38 | NC_017334 | Subsp. *aureus* Strain ST398 isolate SO385 plasmid pS0385-1 | ABS | N/A | ABS | NCBI |
| 39 | NC_017335 | Subsp. *aureus* Strain ST398 isolate SO385 plasmid pS0385-2 | ABS | N/A | ABS | NCBI |
| 40 | NC_017336 | Subsp. *aureus* Strain ST398 isolate SO385 plasmid pS0385-3 | ABS | N/A | ABS | NCBI |
| 41 | NC_017337 | Subsp. *aureus* Strain ED133 | ABS | N/A | ABS | NCBI |
| 42 | NC_017339 | Subsp. *aureus* Strain JKD6159 plasmid pSaa6159 | ABS | N/A | ABS | NCBI |
| 43 | NC_017340 | Strain 04-02981 | ABS | N/A | ABS | NCBI |
| 44 | NC_017341 | Subsp. *aureus* Strain JKD6008 | ABS | N/A | ABS | NCBI |
| 45 | NC_017342 | Subsp. *aureus* Strain TCH60 | ABS | N/A | ABS | NCBI |
| 46 | NC_017343 | Subsp. *aureus* Strain ECT-R2 | ABS | N/A | ABS | NCBI |
| N/A: No applique, ABS: Absent, NCBI: National Center for Biotechnology Information, ENA: European Nucleotide Archive, *Evidence level 4 and 3, *cas* genes | | | | | | |
| **Supplementary table A. Table of CRISPR-Cas system- and CRISPR-Cas system non-bearing strains of *Staphylococcus aureus* (continuation).** | | | | | | |
| **#** | **ACCESSION NUMBER** | **DESCRIPTION** | **CRISPR-Cas system** | **EVIDENCE LEVEL** | ***cas* GENES** | **DATABASE** |
| 47 | NC_017344 | Subsp. *aureus* Strain ECT-R2 plasmid pLUH02 | ABS | N/A | ABS | NCBI |
| 48 | NC_017345 | Subsp. *aureus* Strain TCH60 plasmid | ABS | N/A | ABS | NCBI |
| 49 | NC_017346 | Subsp. *aureus* Strain ECT-R2 plasmid pLUH01 | ABS | N/A | ABS | NCBI |
| 50 | NC_017347 | Subsp. *aureus* Strain T0131 | ABS | N/A | ABS | NCBI |
| 51 | NC_017348 | Subsp. *aureus* Strain LGA251 plasmid pLGA251 | ABS | N/A | ABS | NCBI |
| 52 | NC_017349 | Subsp. *aureus* Strain LGA251 | ABS | N/A | ABS | NCBI |
| 53 | NC_017350 | Subsp. *aureus* Strain 11819-97 plasmid p11819-97 | ABS | N/A | ABS | NCBI |
| 54 | NC_017351 | Subsp. *aureus* Strain 11819-97 | ABS | N/A | ABS | NCBI |
| 55 | NC_017352 | Subsp. *aureus* Strain TW20 plasmid pTW20_1 | ABS | N/A | ABS | NCBI |
| 56 | NC_017763 | Subsp. *aureus* Strain HO 5096 0412 | ABS | N/A | ABS | NCBI |
| 57 | NC_018608 | Strain 08BA02176 | III-A | 4 | *cas1, cas2, cas10, csm2, csm3, csm4, csm5, csm6, cas6* | NCBI |
| 58 | NC_020529 | Subsp. *aureus* Strain ST228 isolate 10338 | ABS | N/A | ABS | NCBI |
| 59 | NC_020530 | Subsp. *aureus* Strain ST228 isolate 10338 plasmid pl1T1 | ABS | N/A | ABS | NCBI |
| 60 | NC_020531 | Subsp. *aureus* Strain ST228 isolate 10338 plasmid pl1T2 | ABS | N/A | ABS | NCBI |
| 61 | NC_020532 | Subsp. *aureus* Strain ST228 isolate 15532 | ABS | N/A | ABS | NCBI |
| 62 | NC_020533 | Subsp. *aureus* Strain ST228 isolate 16035 | ABS | N/A | ABS | NCBI |
| 63 | NC_020534 | Subsp. *aureus* Strain ST228 isolate 16035 plasmid pl4T8 | ABS | N/A | ABS | NCBI |
| 64 | NC_020535 | Subsp. *aureus* Strain ST228 isolate 16125 plasmid pl5S5 | ABS | N/A | ABS | NCBI |
| 65 | NC_020536 | Subsp. *aureus* Strain ST228 isolate 18341 | ABS | N/A | ABS | NCBI |
| 66 | NC_020537 | Subsp. *aureus* Strain ST228 isolate 18412 | ABS | N/A | ABS | NCBI |
| 67 | NC_020538 | Subsp. *aureus* Strain ST228 isolate 18412 plasmid pI7S6 | ABS | N/A | ABS | NCBI |
| 68 | NC_020539 | Subsp. *aureus* Strain ST228 isolate 18583 plasmid pI8T7 | ABS | N/A | ABS | NCBI |
| 69 | NC_020564 | Subsp. *aureus* Strain ST228 isolate 10497 | ABS | N/A | ABS | NCBI |
| N/A: No applique, ABS: Absent, NCBI: National Center for Biotechnology Information, ENA: European Nucleotide Archive, *Evidence level 4 and 3, *cas* genes | | | | | | |
| **Supplementary table A. Table of CRISPR-Cas system- and CRISPR-Cas system non-bearing strains of *Staphylococcus aureus* (continuation).** | | | | | | |
| **#** | **ACCESSION NUMBER** | **DESCRIPTION** | **CRISPR-Cas system** | **EVIDENCE LEVEL** | ***cas* GENES** | **DATABASE** |
| 70 | NC_020565 | Subsp. *aureus* Strain ST228 isolate 15532 plasmid pI3T3 | ABS | N/A | ABS | NCBI |
| 71 | NC_020566 | Subsp. *aureus* Strain ST228 isolate 16125 | ABS | N/A | ABS | NCBI |
| 72 | NC_020567 | Subsp. *aureus* Strain ST228 isolate 18341 plasmid pI6T6 | ABS | N/A | ABS | NCBI |
| 73 | NC_020568 | Subsp. *aureus* Strain ST228 isolate 18583 | ABS | N/A | ABS | NCBI |
| 74 | NC_021552 | Strain CA-347 plasmid | ABS | N/A | ABS | NCBI |
| 75 | NC_021554 | Strain CA-347 | ABS | N/A | ABS | NCBI |
| 76 | NC_021657 | Without Strain plasmid pBmb9393 | ABS | N/A | ABS | NCBI |
| 77 | NC_021670 | Without Strain | ABS | N/A | ABS | NCBI |
| 78 | NC_022113 | Subsp. *aureus* Strain 55_2053 | ABS | N/A | ABS | NCBI |
| 79 | NC_022126 | Subsp. *aureus* Strain 55_2053 plasmid | ABS | N/A | ABS | NCBI |
| 80 | NC_022222 | Subsp. *aureus* Strain 6850 | ABS | N/A | ABS | NCBI |
| 81 | NC_022226 | Subsp. *aureus* Strain CN1 | ABS | N/A | ABS | NCBI |
| 82 | NC_022227 | Subsp. *aureus* Strain CN1 plasmid | ABS | N/A | ABS | NCBI |
| 83 | NC_022228 | Subsp. *aureus* Strain CN1 plasmid | ABS | N/A | ABS | NCBI |
| 84 | NC_022442 | Subsp. *aureus* Strain SA957 | ABS | N/A | ABS | NCBI |
| 85 | NC_022443 | Subsp. *aureus* Strain SA40 | ABS | N/A | ABS | NCBI |
| 86 | NC_022604 | Subsp. *aureus* Strain Z172 | ABS | N/A | ABS | NCBI |
| 87 | NC_022605 | Subsp. *aureus* Strain Z172 plasmid pZ172_2 | ABS | N/A | ABS | NCBI |
| 88 | NC_022610 | Subsp. *aureus* Strain Z172 plasmid pZ172_1 | ABS | N/A | ABS | NCBI |
| 89 | NC_021670 | Without Strain | ABS | N/A | ABS | NCBI |
| 90 | NZ_017338 | Subsp. *aureus* Strain JKD6159 | ABS | N/A | ABS | NCBI |
| 91 | NZ_AP014652 | Subsp. *aureus* Strain TMUS2126 | ABS | N/A | ABS | NCBI |
| 92 | NZ_AP014653 | Subsp. *aureus* Strain TMUS2134 | ABS | N/A | ABS | NCBI |
| N/A: No applique, ABS: Absent, NCBI: National Center for Biotechnology Information, ENA: European Nucleotide Archive, *Evidence level 4 and 3, *cas* genes | | | | | | |
| **Supplementary table A. Table of CRISPR-Cas system- and CRISPR-Cas system non-bearing strains of *Staphylococcus aureus* (continuation).** | | | | | | |
| **#** | **ACCESSION NUMBER** | **DESCRIPTION** | **CRISPR-Cas system** | **EVIDENCE LEVEL** | ***cas* GENES** | **DATABASE** |
| 93 | NZ_AP014921 | Strain JH4899 | ABS | N/A | ABS | NCBI |
| 94 | NZ_AP014922 | Strain JH4899 plasmid pJSA01 | ABS | N/A | ABS | NCBI |
| 95 | NZ_AP014942 | Strain FDA209P | ABS | N/A | ABS | NCBI |
| 96 | NZ_AP014943 | Strain FDA209P plasmid pFDA209P | ABS | N/A | ABS | NCBI |
| 97 | NZ_AP017320 | Strain MI | ABS | N/A | ABS | NCBI |
| 98 | NZ_AP017321 | Strain MI plasmid pMI | ABS | N/A | ABS | NCBI |
| 99 | NZ_AP017377 | Strain OC8 | ABS | N/A | ABS | NCBI |
| 100 | NZ_AP017891 | Strain GN3 | ABS | N/A | ABS | NCBI |
| 101 | NZ_AP017922 | Strain JP080 | ABS | N/A | ABS | NCBI |
| 102 | NZ_AP017923 | Strain JP080 plasmid pJP080 | ABS | N/A | ABS | NCBI |
| 103 | NZ_AP018349 | Strain GN1 | ABS | N/A | ABS | NCBI |
| 104 | NZ_AP018922 | Strain JMUB1273 | ABS | N/A | ABS | NCBI |
| 105 | NZ_AP018923 | Strain JMUB3031 | ABS | N/A | ABS | NCBI |
| 106 | NZ_AP018924 | Strain JMUB3031 plasmid pJMUB3031 | ABS | N/A | ABS | NCBI |
| 107 | NZ_AP019305 | Strain TUM9458 | ABS | N/A | ABS | NCBI |
| 108 | NZ_AP019306 | Strain TUM9463 | ABS | N/A | ABS | NCBI |
| 109 | NZ_AP019542 | Strain KG-03 | ABS | N/A | ABS | NCBI |
| 110 | NZ_AP019543 | Strain KG-18 | ABS | N/A | ABS | NCBI |
| 111 | NZ_AP019544 | Strain KG-18 plasmid pKG-18 | ABS | N/A | ABS | NCBI |
| 112 | NZ_AP019545 | Strain KG-22 | ABS | N/A | ABS | NCBI |
| 113 | NZ_AP019546 | Strain KG-22 plasmid pKG-22 | ABS | N/A | ABS | NCBI |
| 114 | NZ_AP019712 | Strain Tokyo12480 | ABS | N/A | ABS | NCBI |
| 115 | NZ_AP019751 | Strain JRA307 | ABS | N/A | ABS | NCBI |
| N/A: No applique, ABS: Absent, NCBI: National Center for Biotechnology Information, ENA: European Nucleotide Archive, *Evidence level 4 and 3, *cas* genes | | | | | | |
| **Supplementary table A. Table of CRISPR-Cas system- and CRISPR-Cas system non-bearing strains of *Staphylococcus aureus* (continuation).** | | | | | | |
| **#** | **ACCESSION NUMBER** | **DESCRIPTION** | **CRISPR-Cas system** | **EVIDENCE LEVEL** | ***cas* GENES** | **DATABASE** |
| 116 | NZ_AP019752 | Strain JRA307 plasmid pJRA307-1 | ABS | N/A | ABS | NCBI |
| 117 | NZ_AP020311 | Strain KUH140013 | ABS | N/A | ABS | NCBI |
| 118 | NZ_AP020312 | Strain KUH140013 plasmid p01KUH140013 | ABS | N/A | ABS | NCBI |
| 119 | NZ_AP020313 | Strain KUH140046 | ABS | N/A | ABS | NCBI |
| 120 | NZ_AP020314 | Strain KUH140046 plasmid p01KUH140046 | ABS | N/A | ABS | NCBI |
| 121 | NZ_AP020315 | Strain KUH140087 | III-A | 3 | *cas6, csm6, csm5, csm4, csm3, csm2, cas10, cas2, cas1* | NCBI |
| 122 | NZ_AP020316 | Strain KUH140331 | ABS | N/A | ABS | NCBI |
| 123 | NZ_AP020317 | Strain KUH140331 plasmid p01KUH140331 | ABS | N/A | ABS | NCBI |
| 124 | NZ_AP020318 | Strain KUH180038 | ABS | N/A | ABS | NCBI |
| 125 | NZ_AP020319 | Strain KUH180038 plasmid p01KUH180038 | ABS | N/A | ABS | NCBI |
| 126 | NZ_AP020320 | Strain KUH180062 | ABS | N/A | ABS | NCBI |
| 127 | NZ_AP020321 | Strain KUH180062 plasmid p01KUH180062 | ABS | N/A | ABS | NCBI |
| 128 | NZ_AP020322 | Strain KUH180129 | ABS | N/A | ABS | NCBI |
| 129 | NZ_AP020323 | Strain KUH180129 plasmid p01KUH180129 | ABS | N/A | ABS | NCBI |
| 130 | NZ_AP020324 | Strain KUN1163 | ABS | N/A | ABS | NCBI |
| 131 | NZ_AP020325 | Strain KUN1163 plasmid p01KUN1163 | ABS | N/A | ABS | NCBI |
| 132 | NZ_CP006630 | Subsp. *aureus* Strain SA268 | ABS | N/A | ABS | NCBI |
| 133 | NZ_CP007176 | Strain USA300-ISMMS1 | ABS | N/A | ABS | NCBI |
| 134 | NZ_CP007177 | Strain USA300-ISMMS1 plasmid pUSA01-ISMMS | ABS | N/A | ABS | NCBI |
| 135 | NZ_CP007178 | Strain USA300-ISMMS1 plasmid pUSA02-ISMMS | ABS | N/A | ABS | NCBI |
| 136 | NZ_CP007447 | Strain XN108 | ABS | N/A | ABS | NCBI |
| 137 | NZ_CP007454 | Strain 502A isolate RN6607 | ABS | N/A | ABS | NCBI |
| 138 | NZ_CP007455 | Strain 502A isolate RN6607 plasmid | ABS | N/A | ABS | NCBI |
| N/A: No applique, ABS: Absent, NCBI: National Center for Biotechnology Information, ENA: European Nucleotide Archive, *Evidence level 4 and 3, *cas* genes | | | | | | |
| **Supplementary table A. Table of CRISPR-Cas system- and CRISPR-Cas system non-bearing strains of *Staphylococcus aureus* (continuation).** | | | | | | |
| **#** | **ACCESSION NUMBER** | **DESCRIPTION** | **CRISPR-Cas system** | **EVIDENCE LEVEL** | ***cas* GENES** | **DATABASE** |
| 139 | NZ_CP007499 | Strain 2395_USA500 | ABS | N/A | ABS | NCBI |
| 140 | NZ_CP007500 | Strain 2395_USA500 plasmid pUSA500 | ABS | N/A | ABS | NCBI |
| 141 | NZ_CP007539 | Subsp. *aureus* Strain FDAARGOS_5 | ABS | N/A | ABS | NCBI |
| 142 | NZ_CP007657 | Strain V2200 | ABS | N/A | ABS | NCBI |
| 143 | NZ_CP007658 | Strain V2200 plasmid pV2200 | ABS | N/A | ABS | NCBI |
| 144 | NZ_CP007659 | Subsp. *aureus* Strain H-EMRSA-15 | ABS | N/A | ABS | NCBI |
| 145 | NZ_CP007670 | Strain M121 | ABS | N/A | ABS | NCBI |
| 146 | NZ_CP007671 | Strain M121 plasmid pM121 | ABS | N/A | ABS | NCBI |
| 147 | NZ_CP007672 | Strain CA12 | ABS | N/A | ABS | NCBI |
| 148 | NZ_CP007673 | Strain CA12 plasmid pCAS12 | ABS | N/A | ABS | NCBI |
| 149 | NZ_CP007674 | Strain CA15 | ABS | N/A | ABS | NCBI |
| 150 | NZ_CP007675 | Strain CA15 plasmid pCA15 | ABS | N/A | ABS | NCBI |
| 151 | NZ_CP007676 | Strain HUV05 | ABS | N/A | ABS | NCBI |
| 152 | NZ_CP007677 | Strain HUV05 plasmid pHUV05-01 | ABS | N/A | ABS | NCBI |
| 153 | NZ_CP007678 | Strain HUV05 plasmid pHUV05-02 | ABS | N/A | ABS | NCBI |
| 154 | NZ_CP007679 | Strain HUV05 plasmid pHUV05-03 | ABS | N/A | ABS | NCBI |
| 155 | NZ_CP007690 | Strain UA-S391_USA300 | ABS | N/A | ABS | NCBI |
| 156 | NZ_CP009361 | Subsp. *aureus* Strain ATCC 25923 | ABS | N/A | ABS | NCBI |
| 157 | NZ_CP009362 | Subsp. *aureus* Strain ATCC 25923 plasmid pS1945 | ABS | N/A | ABS | NCBI |
| 158 | NZ_CP009423 | Subsp. *aureus* Strain USA300_SUR1 | ABS | N/A | ABS | NCBI |
| 159 | NZ_CP009424 | Subsp. *aureus* Strain USA300_SUR1 plasmid pSUR1A | ABS | N/A | ABS | NCBI |
| 160 | NZ_CP009425 | Subsp. *aureus* Strain USA300_SUR1 plasmid pSUR1B | ABS | N/A | ABS | NCBI |
| 161 | NZ_CP009554 | Subsp. *aureus* Strain FORC 001 | ABS | N/A | ABS | NCBI |
| N/A: No applique, ABS: Absent, NCBI: National Center for Biotechnology Information, ENA: European Nucleotide Archive, *Evidence level 4 and 3, *cas* genes | | | | | | |
| **Supplementary table A. Table of CRISPR-Cas system- and CRISPR-Cas system non-bearing strains of *Staphylococcus aureus* (continuation).** | | | | | | |
| **#** | **ACCESSION NUMBER** | **DESCRIPTION** | **CRISPR-Cas system** | **EVIDENCE LEVEL** | ***cas* GENES** | **DATABASE** |
| 162 | NZ_CP009681 | Subsp. *aureus* Strain Gv69 | ABS | N/A | ABS | NCBI |
| 163 | NZ_CP009828 | Strain MS4 | ABS | N/A | ABS | NCBI |
| 164 | NZ_CP010295 | Strain 29b_MRSA | ABS | N/A | ABS | NCBI |
| 165 | NZ_CP010296 | Strain 31b_MRSA | ABS | N/A | ABS | NCBI |
| 166 | NZ_CP010297 | Strain 33b | ABS | N/A | ABS | NCBI |
| 167 | NZ_CP010298 | Strain 26b_MRSA | ABS | N/A | ABS | NCBI |
| 168 | NZ_CP010299 | Strain 25b_MRSA | ABS | N/A | ABS | NCBI |
| 169 | NZ_CP010300 | Strain 27b_MRSA | ABS | N/A | ABS | NCBI |
| 170 | NZ_CP010402 | Subsp. *aureus* Strain GR2 | ABS | N/A | ABS | NCBI |
| 171 | NZ_CP010403 | Subsp. *aureus* Strain GR2 plasmid pGR2A | ABS | N/A | ABS | NCBI |
| 172 | NZ_CP010404 | Subsp. *aureus* Strain GR2 plasmid pGR2B | ABS | N/A | ABS | NCBI |
| 173 | NZ_CP010526 | Subsp. *aureus* ST772-MRSA-V Strain DAR4145 | ABS | N/A | ABS | NCBI |
| 174 | NZ_CP010890 | Strain SA564 | ABS | N/A | ABS | NCBI |
| 175 | NZ_CP010891 | Strain SA564 plasmid pSA564 | ABS | N/A | ABS | NCBI |
| 176 | NZ_CP010998 | Strain FORC_012 | ABS | N/A | ABS | NCBI |
| 177 | NZ_CP011147 | Strain FCFHV36 | ABS | N/A | ABS | NCBI |
| 178 | NZ_CP011526 | Subsp. *aureus* Strain DSM 20231 | ABS | N/A | ABS | NCBI |
| 179 | NZ_CP011527 | Subsp. *aureus* Strain DSM 20231 plasmid | ABS | N/A | ABS | NCBI |
| 180 | NZ_CP011528 | Strain RKI4 | ABS | N/A | ABS | NCBI |
| 181 | NZ_CP011529 | Strain RKI4 plasmid | ABS | N/A | ABS | NCBI |
| 182 | NZ_CP011685 | Strain ZJ5499 | ABS | N/A | ABS | NCBI |
| 183 | NZ_CP012011 | Subsp. *aureus* Strain HC1340 | ABS | N/A | ABS | NCBI |
| 184 | NZ_CP012012 | Subsp. *aureus* Strain HC1335 | ABS | N/A | ABS | NCBI |
| N/A: No applique, ABS: Absent, NCBI: National Center for Biotechnology Information, ENA: European Nucleotide Archive, *Evidence level 4 and 3, *cas* genes | | | | | | |
| **Supplementary table A. Table of CRISPR-Cas system- and CRISPR-Cas system non-bearing strains of *Staphylococcus aureus* (continuation).** | | | | | | |
| **#** | **ACCESSION NUMBER** | **DESCRIPTION** | **CRISPR-Cas system** | **EVIDENCE LEVEL** | ***cas* GENES** | **DATABASE** |
| 185 | NZ_CP012013 | Subsp. *aureus* Strain Be62 | ABS | N/A | ABS | NCBI |
| 186 | NZ_CP012014 | Subsp. *aureus* Strain Be62 plasmid pBe62 | ABS | N/A | ABS | NCBI |
| 187 | NZ_CP012015 | Subsp. *aureus* Strain Gv51 | ABS | N/A | ABS | NCBI |
| 188 | NZ_CP012016 | Subsp. *aureus* Strain Gv51 plasmid pGv51 | ABS | N/A | ABS | NCBI |
| 189 | NZ_CP012017 | Subsp. *aureus* Strain Gv51 plasmid pGv88 | ABS | N/A | ABS | NCBI |
| 190 | NZ_CP012018 | Subsp. *aureus* Strain Gv88 | ABS | N/A | ABS | NCBI |
| 191 | NZ_CP012118 | Subsp. *aureus* Strain USA300_2014.C01 plasmid pC01b | ABS | N/A | ABS | NCBI |
| 192 | NZ_CP012119 | Subsp. *aureus* Strain USA300_2014.C01 | ABS | N/A | ABS | NCBI |
| 193 | NZ_CP012120 | Subsp. *aureus* Strain USA300_2014.C02 | ABS | N/A | ABS | NCBI |
| 194 | NZ_CP012121 | Subsp. *aureus* Strain USA300_2014.C02 plasmid pC02 | ABS | N/A | ABS | NCBI |
| 195 | NZ_CP012409 | Subsp. *aureus* Strain Tager 104 | ABS | N/A | ABS | NCBI |
| 196 | NZ_CP012593 | Subsp. *aureus* Strain HOU1444-VR | ABS | N/A | ABS | NCBI |
| 197 | NZ_CP012594 | Subsp. *aureus* Strain HOU1444-VR plasmid pVR-MSSA_01 | ABS | N/A | ABS | NCBI |
| 198 | NZ_CP012595 | Subsp. *aureus* Strain HOU1444-VR plasmid pVR-MSSA_02 | ABS | N/A | ABS | NCBI |
| 199 | NZ_CP012596 | Subsp. *aureus* Strain HOU1444-VR plasmid pVR-MSSA_03 | ABS | N/A | ABS | NCBI |
| 200 | NZ_CP012692 | Strain FORC 027 | ABS | N/A | ABS | NCBI |
| 201 | NZ_CP012756 | Subsp. *aureus* Strain JS395 | III-A | 4 | *cas1, cas2, cas10, csm2, csm3, csm4, csm5, csm6, cas6* | NCBI |
| 202 | NZ_CP012757 | Subsp. *aureus* Strain JS395 plasmid | ABS | N/A | ABS | NCBI |
| 203 | NZ_CP012970 | Strain ST20130939 | ABS | N/A | ABS | NCBI |
| 204 | NZ_CP012971 | Strain ST20130939 plasmid pST20130939 | ABS | N/A | ABS | NCBI |
| 205 | NZ_CP012972 | Strain ST0130938 | ABS | N/A | ABS | NCBI |
| 206 | NZ_CP012973 | Strain ST0130938 plasmid pST0130938 | ABS | N/A | ABS | NCBI |
| 207 | NZ_CP012974 | Strain ST20130943 | ABS | N/A | ABS | NCBI |
| N/A: No applique, ABS: Absent, NCBI: National Center for Biotechnology Information, ENA: European Nucleotide Archive, *Evidence level 4 and 3, *cas* genes | | | | | | |
| **Supplementary table A. Table of CRISPR-Cas system- and CRISPR-Cas system non-bearing strains of *Staphylococcus aureus* (continuation).** | | | | | | |
| **#** | **ACCESSION NUMBER** | **DESCRIPTION** | **CRISPR-Cas system** | **EVIDENCE LEVEL** | ***cas* GENES** | **DATABASE** |
| 208 | NZ_CP012975 | Strain ST20130943 plasmid pST20130943 | ABS | N/A | ABS | NCBI |
| 209 | NZ_CP012976 | Strain ST20130942 | ABS | N/A | ABS | NCBI |
| 210 | NZ_CP012977 | Strain ST20130942 plasmid pST20130942 | ABS | N/A | ABS | NCBI |
| 211 | NZ_CP012978 | Strain ST20130941 | ABS | N/A | ABS | NCBI |
| 212 | NZ_CP012979 | Strain ST20130940 | ABS | N/A | ABS | NCBI |
| 213 | NZ_CP012980 | Strain ST20130940 plasmid pST20130940 | ABS | N/A | ABS | NCBI |
| 214 | NZ_CP013132 | Strain FORC_026 | ABS | N/A | ABS | NCBI |
| 215 | NZ_CP013137 | Strain XQ | ABS | N/A | ABS | NCBI |
| 216 | NZ_CP013182 | Strain SA40TW | ABS | N/A | ABS | NCBI |
| 217 | NZ_CP013218 | Subsp. *aureus* Strain LA-MRSA ST398 isolate E154 | ABS | N/A | ABS | NCBI |
| 218 | NZ_CP013227 | Subsp. *aureus* Strain UTSW MRSA 55 plasmid pUTSW55_1 | ABS | N/A | ABS | NCBI |
| 219 | NZ_CP013228 | Subsp. *aureus* Strain UTSW MRSA 55 plasmid pUTSW55_2 | ABS | N/A | ABS | NCBI |
| 220 | NZ_CP013229 | Subsp. *aureus* Strain UTSW MRSA 55 plasmid pUTSW55_3 | ABS | N/A | ABS | NCBI |
| 221 | NZ_CP013230 | Subsp. *aureus* Strain UTSW MRSA 55 plasmid pUTSW55_4 | ABS | N/A | ABS | NCBI |
| 222 | NZ_CP013231 | Strain UTSW MRSA 55 | ABS | N/A | ABS | NCBI |
| 223 | NZ_CP013616 | Strain RIVM1295 | ABS | N/A | ABS | NCBI |
| 224 | NZ_CP013617 | Strain RIVM1295 plasmid pRIVM1295-1 | ABS | N/A | ABS | NCBI |
| 225 | NZ_CP013618 | Strain RIVM1295 plasmid pRIVM1295-2 | ABS | N/A | ABS | NCBI |
| 226 | NZ_CP013619 | Strain RIVM1607 | ABS | N/A | ABS | NCBI |
| 227 | NZ_CP013620 | Strain RIVM1607 plasmid pRIVM1607 | ABS | N/A | ABS | NCBI |
| 228 | NZ_CP013621 | Strain RIVM3897 | ABS | N/A | ABS | NCBI |
| 229 | NZ_CP013953 | Strain NCCP14558 | ABS | N/A | ABS | NCBI |
| 230 | NZ_CP013954 | Strain NCCP14558 plasmid pNCCP14558 | ABS | N/A | ABS | NCBI |
| N/A: No applique, ABS: Absent, NCBI: National Center for Biotechnology Information, ENA: European Nucleotide Archive, *Evidence level 4 and 3, *cas* genes | | | | | | |
| **Supplementary table A. Table of CRISPR-Cas system- and CRISPR-Cas system non-bearing strains of *Staphylococcus aureus* (continuation).** | | | | | | |
| **#** | **ACCESSION NUMBER** | **DESCRIPTION** | **CRISPR-Cas system** | **EVIDENCE LEVEL** | ***cas* GENES** | **DATABASE** |
| 231 | NZ_CP013955 | Strain NCCP14562 | ABS | N/A | ABS | NCBI |
| 232 | NZ_CP013956 | Strain NCCP14562 plasmid pNCCP14562 | ABS | N/A | ABS | NCBI |
| 233 | NZ_CP013957 | Strain V521 | ABS | N/A | ABS | NCBI |
| 234 | NZ_CP013958 | Strain V521 plasmid pV521 | ABS | N/A | ABS | NCBI |
| 235 | NZ_CP014063 | Strain FDAARGOS_159 plasmid unnamed | ABS | N/A | ABS | NCBI |
| 236 | NZ_CP014064 | Strain FDAARGOS_159 | ABS | N/A | ABS | NCBI |
| 237 | NZ_CP014362 | Strain USA300-SUR1 | ABS | N/A | ABS | NCBI |
| 238 | NZ_CP014363 | Strain USA300-SUR1 plasmid pUSA01-1-SUR1 | ABS | N/A | ABS | NCBI |
| 239 | NZ_CP014364 | Strain USA300-SUR1 plasmid pUSA04-1-SUR1 | ABS | N/A | ABS | NCBI |
| 240 | NZ_CP014365 | Strain USA300-SUR2 | ABS | N/A | ABS | NCBI |
| 241 | NZ_CP014366 | Strain USA300-SUR2 plasmid pUSA01-1-SUR2 | ABS | N/A | ABS | NCBI |
| 242 | NZ_CP014367 | Strain USA300-SUR2 plasmid pUSA04-1-SUR2 | ABS | N/A | ABS | NCBI |
| 243 | NZ_CP014368 | Strain USA300-SUR3 | ABS | N/A | ABS | NCBI |
| 244 | NZ_CP014369 | Strain USA300-SUR3 plasmid pUSA01-1-SUR3 | ABS | N/A | ABS | NCBI |
| 245 | NZ_CP014370 | Strain USA300-SUR3 plasmid pUSA04-1-SUR3 | ABS | N/A | ABS | NCBI |
| 246 | NZ_CP014371 | Strain USA300-SUR4 | ABS | N/A | ABS | NCBI |
| 247 | NZ_CP014372 | Strain USA300-SUR4 plasmid pUSA01-1-SUR4 | ABS | N/A | ABS | NCBI |
| 248 | NZ_CP014373 | Strain USA300-SUR4 plasmid pUSA04-2-SUR4 | ABS | N/A | ABS | NCBI |
| 249 | NZ_CP014374 | Strain USA300-SUR4 plasmid pUSA05-1-SUR4 | ABS | N/A | ABS | NCBI |
| 250 | NZ_CP014375 | Strain USA300-SUR4 plasmid pUSA06-1-SUR4 | ABS | N/A | ABS | NCBI |
| 251 | NZ_CP014376 | Strain USA300-SUR5 | ABS | N/A | ABS | NCBI |
| 252 | NZ_CP014377 | Strain USA300-SUR5 plasmid pUSA01-1-SUR5 | ABS | N/A | ABS | NCBI |
| 253 | NZ_CP014378 | Strain USA300-SUR5 plasmid pUSA04-2-SUR5 | ABS | N/A | ABS | NCBI |
| N/A: No applique, ABS: Absent, NCBI: National Center for Biotechnology Information, ENA: European Nucleotide Archive, *Evidence level 4 and 3, *cas* genes | | | | | | |
| **Supplementary table A. Table of CRISPR-Cas system- and CRISPR-Cas system non-bearing strains of *Staphylococcus aureus* (continuation).** | | | | | | |
| **#** | **ACCESSION NUMBER** | **DESCRIPTION** | **CRISPR-Cas system** | **EVIDENCE LEVEL** | ***cas* GENES** | **DATABASE** |
| 254 | NZ_CP014379 | Strain USA300-SUR5 plasmid pUSA05-1-SUR5 | ABS | N/A | ABS | NCBI |
| 255 | NZ_CP014380 | Strain USA300-SUR5 plasmid pUSA06-1-SUR5 | ABS | N/A | ABS | NCBI |
| 256 | NZ_CP014381 | Strain USA300-SUR6 | ABS | N/A | ABS | NCBI |
| 257 | NZ_CP014382 | Strain USA300-SUR6 plasmid pUSA01-1-SUR6 | ABS | N/A | ABS | NCBI |
| 258 | NZ_CP014383 | Strain USA300-SUR6 plasmid pUSA04-1-SUR6 | ABS | N/A | ABS | NCBI |
| 259 | NZ_CP014384 | Strain USA300-SUR7 | ABS | N/A | ABS | NCBI |
| 260 | NZ_CP014385 | Strain USA300-SUR7 plasmid pUSA01-1-SUR7 | ABS | N/A | ABS | NCBI |
| 261 | NZ_CP014386 | Strain USA300-SUR7 plasmid pUSA04-3-SUR7 | ABS | N/A | ABS | NCBI |
| 262 | NZ_CP014387 | Strain USA300-SUR8 | ABS | N/A | ABS | NCBI |
| 263 | NZ_CP014388 | Strain USA300-SUR8 plasmid pUSA01-1-SUR8 | ABS | N/A | ABS | NCBI |
| 264 | NZ_CP014389 | Strain USA300-SUR8 plasmid pUSA04-2-SUR8 | ABS | N/A | ABS | NCBI |
| 265 | NZ_CP014390 | Strain USA300-SUR8 plasmid pUSA05-1-SUR8 | ABS | N/A | ABS | NCBI |
| 266 | NZ_CP014391 | Strain USA300-SUR8 plasmid pUSA06-1-SUR8 | ABS | N/A | ABS | NCBI |
| 267 | NZ_CP014392 | Strain USA300-SUR9 | ABS | N/A | ABS | NCBI |
| 268 | NZ_CP014393 | Strain USA300-SUR9 plasmid pUSA01-1-SUR9 | ABS | N/A | ABS | NCBI |
| 269 | NZ_CP014394 | Strain USA300-SUR9 plasmid pUSA04-2-SUR9 | ABS | N/A | ABS | NCBI |
| 270 | NZ_CP014395 | Strain USA300-SUR9 plasmid pUSA05-1-SUR9 | ABS | N/A | ABS | NCBI |
| 271 | NZ_CP014396 | Strain USA300-SUR9 plasmid pUSA06-1-SUR9 | ABS | N/A | ABS | NCBI |
| 272 | NZ_CP014397 | Strain USA300-SUR10 | ABS | N/A | ABS | NCBI |
| 273 | NZ_CP014398 | Strain USA300-SUR10 plasmid pUSA01-1-SUR10 | ABS | N/A | ABS | NCBI |
| 274 | NZ_CP014399 | Strain USA300-SUR10 plasmid pUSA04-1-SUR10 | ABS | N/A | ABS | NCBI |
| 275 | NZ_CP014400 | Strain USA300-SUR10 plasmid pUSA05-1-SUR10 | ABS | N/A | ABS | NCBI |
| 276 | NZ_CP014401 | Strain USA300-SUR10 plasmid pUSA06-1-SUR10 | ABS | N/A | ABS | NCBI |
| N/A: No applique, ABS: Absent, NCBI: National Center for Biotechnology Information, ENA: European Nucleotide Archive, *Evidence level 4 and 3, *cas* genes | | | | | | |
| **Supplementary table A. Table of CRISPR-Cas system- and CRISPR-Cas system non-bearing strains of *Staphylococcus aureus* (continuation).** | | | | | | |
| **#** | **ACCESSION NUMBER** | **DESCRIPTION** | **CRISPR-Cas system** | **EVIDENCE LEVEL** | ***cas* GENES** | **DATABASE** |
| 277 | NZ_CP014402 | Strain USA300-SUR11 | ABS | N/A | ABS | NCBI |
| 278 | NZ_CP014403 | Strain USA300-SUR11 plasmid pUSA01-1-SUR11 | ABS | N/A | ABS | NCBI |
| 279 | NZ_CP014404 | Strain USA300-SUR11 plasmid pUSA04-2-SUR11 | ABS | N/A | ABS | NCBI |
| 280 | NZ_CP014405 | Strain USA300-SUR11 plasmid pUSA05-1-SUR11 | ABS | N/A | ABS | NCBI |
| 281 | NZ_CP014406 | Strain USA300-SUR11 plasmid pUSA06-1-SUR11 | ABS | N/A | ABS | NCBI |
| 282 | NZ_CP014407 | Strain USA300-SUR12 | ABS | N/A | ABS | NCBI |
| 283 | NZ_CP014408 | Strain USA300-SUR12 plasmid pUSA04-1-SUR12 | ABS | N/A | ABS | NCBI |
| 284 | NZ_CP014409 | Strain USA300-SUR13 | ABS | N/A | ABS | NCBI |
| 285 | NZ_CP014410 | Strain USA300-SUR13 plasmid pUSA01-1-SUR13 | ABS | N/A | ABS | NCBI |
| 286 | NZ_CP014411 | Strain USA300-SUR13 plasmid pUSA04-1-SUR13 | ABS | N/A | ABS | NCBI |
| 287 | NZ_CP014412 | Strain USA300-SUR14 | ABS | N/A | ABS | NCBI |
| 288 | NZ_CP014413 | Strain USA300-SUR14 plasmid pUSA01-1-SUR14 | ABS | N/A | ABS | NCBI |
| 289 | NZ_CP014414 | Strain USA300-SUR14 plasmid pUSA04-1-SUR14 | ABS | N/A | ABS | NCBI |
| 290 | NZ_CP014415 | Strain USA300-SUR15 | ABS | N/A | ABS | NCBI |
| 291 | NZ_CP014416 | Strain USA300-SUR15 plasmid pUSA04-1-SUR15 | ABS | N/A | ABS | NCBI |
| 292 | NZ_CP014417 | Strain USA300-SUR15 plasmid pUSA05-1-SUR15 | ABS | N/A | ABS | NCBI |
| 293 | NZ_CP014418 | Strain USA300-SUR15 plasmid pUSA07-1-SUR15 | ABS | N/A | ABS | NCBI |
| 294 | NZ_CP014419 | Strain USA300-SUR15 plasmid pUSA08-1-SUR15 | ABS | N/A | ABS | NCBI |
| 295 | NZ_CP014420 | Strain USA300-SUR16 | ABS | N/A | ABS | NCBI |
| 296 | NZ_CP014421 | Strain USA300-SUR16 plasmid pUSA01-1-SUR16 | ABS | N/A | ABS | NCBI |
| 297 | NZ_CP014422 | Strain USA300-SUR16 plasmid pUSA04-1-SUR16 | ABS | N/A | ABS | NCBI |
| 298 | NZ_CP014423 | Strain USA300-SUR17 | ABS | N/A | ABS | NCBI |
| 299 | NZ_CP014424 | Strain USA300-SUR17 plasmid pUSA01-1-SUR17 | ABS | N/A | ABS | NCBI |
| N/A: No applique, ABS: Absent, NCBI: National Center for Biotechnology Information, ENA: European Nucleotide Archive, *Evidence level 4 and 3, *cas* genes | | | | | | |
| **Supplementary table A. Table of CRISPR-Cas system- and CRISPR-Cas system non-bearing strains of *Staphylococcus aureus* (continuation).** | | | | | | |
| **#** | **ACCESSION NUMBER** | **DESCRIPTION** | **CRISPR-Cas system** | **EVIDENCE LEVEL** | ***cas* GENES** | **DATABASE** |
| 300 | NZ_CP014425 | Strain USA300-SUR17 plasmid pUSA04-1-SUR17 | ABS | N/A | ABS | NCBI |
| 301 | NZ_CP014426 | Strain USA300-SUR18 | ABS | N/A | ABS | NCBI |
| 302 | NZ_CP014427 | Strain USA300-SUR18 plasmid pUSA01-1-SUR18 | ABS | N/A | ABS | NCBI |
| 303 | NZ_CP014428 | Strain USA300-SUR18 plasmid pUSA04-1-SUR18 | ABS | N/A | ABS | NCBI |
| 304 | NZ_CP014429 | Strain USA300-SUR19 | ABS | N/A | ABS | NCBI |
| 305 | NZ_CP014430 | Strain USA300-SUR19 plasmid pUSA01-1-SUR19 | ABS | N/A | ABS | NCBI |
| 306 | NZ_CP014432 | Strain USA300-SUR20 | ABS | N/A | ABS | NCBI |
| 307 | NZ_CP014433 | Strain USA300-SUR20 plasmid pUSA01-1-SUR20 | ABS | N/A | ABS | NCBI |
| 308 | NZ_CP014434 | Strain USA300-SUR20 plasmid pUSA04-1-SUR20 | ABS | N/A | ABS | NCBI |
| 309 | NZ_CP014435 | Strain USA300-SUR21 | ABS | N/A | ABS | NCBI |
| 310 | NZ_CP014436 | Strain USA300-SUR21 plasmid pUSA01-1-SUR21 | ABS | N/A | ABS | NCBI |
| 311 | NZ_CP014437 | Strain USA300-SUR21 plasmid pUSA04-1-SUR21 | ABS | N/A | ABS | NCBI |
| 312 | NZ_CP014438 | Strain USA300-SUR22 | ABS | N/A | ABS | NCBI |
| 313 | NZ_CP014439 | Strain USA300-SUR22 plasmid pUSA01-1-SUR22 | ABS | N/A | ABS | NCBI |
| 314 | NZ_CP014440 | Strain USA300-SUR22 plasmid pUSA04-1-SUR22 | ABS | N/A | ABS | NCBI |
| 315 | NZ_CP014441 | Strain USA300-SUR23 | ABS | N/A | ABS | NCBI |
| 316 | NZ_CP014442 | Strain USA300-SUR23 plasmid pUSA01-1-SUR23 | ABS | N/A | ABS | NCBI |
| 317 | NZ_CP014443 | Strain USA300-SUR23 plasmid pUSA04-1-SUR23 | ABS | N/A | ABS | NCBI |
| 318 | NZ_CP014444 | Strain USA300-SUR24 | ABS | N/A | ABS | NCBI |
| 319 | NZ_CP014445 | Strain USA300-SUR24 plasmid pUSA04-1-SUR24 | ABS | N/A | ABS | NCBI |
| 320 | NZ_CP014446 | Strain USA300-SUR24 plasmid pUSA05-1-SUR24 | ABS | N/A | ABS | NCBI |
| 321 | NZ_CP014447 | Strain USA300-SUR24 plasmid pUSA07-1-SUE24 | ABS | N/A | ABS | NCBI |
| 322 | NZ_CP014448 | Strain USA300-SUR24 plasmid pUSA08-1-SUR24 | ABS | N/A | ABS | NCBI |
| N/A: No applique, ABS: Absent, NCBI: National Center for Biotechnology Information, ENA: European Nucleotide Archive, *Evidence level 4 and 3, *cas* genes | | | | | | |
| **Supplementary table A. Table of CRISPR-Cas system- and CRISPR-Cas system non-bearing strains of *Staphylococcus aureus* (continuation).** | | | | | | |
| **#** | **ACCESSION NUMBER** | **DESCRIPTION** | **CRISPR-Cas system** | **EVIDENCE LEVEL** | ***cas* GENES** | **DATABASE** |
| 323 | NZ_CP014694 | Subsp. *aureus* Strain LA-MRSA ST398 plasmid plinE154 | ABS | N/A | ABS | NCBI |
| 324 | NZ_CP014695 | Subsp. *aureus* Strain LA-MRSA ST398 plasmid paadD | ABS | N/A | ABS | NCBI |
| 325 | NZ_CP014791 | Strain MCRF184 | ABS | N/A | ABS | NCBI |
| 326 | NZ_CP015173 | Strain RIVM6519 | ABS | N/A | ABS | NCBI |
| 327 | NZ_CP015174 | Strain RIVM6519 plasmid pRIVM6519-1 | ABS | N/A | ABS | NCBI |
| 328 | NZ_CP015175 | Strain RIVM6519 plasmid pRIVM6519-2 | ABS | N/A | ABS | NCBI |
| 329 | NZ_CP015447 | Strain M92 | ABS | N/A | ABS | NCBI |
| 330 | NZ_CP015645 | Strain 08-02119 | ABS | N/A | ABS | NCBI |
| 331 | NZ_CP015646 | Strain 08-02300 | ABS | N/A | ABS | NCBI |
| 332 | NZ_CP015817 | Strain FORC_039 | ABS | N/A | ABS | NCBI |
| 333 | NZ_CP015818 | Strain FORC_039 plasmid | ABS | N/A | ABS | NCBI |
| 334 | NZ_CP016398 | Strain FORC_40 | ABS | N/A | ABS | NCBI |
| 335 | NZ_CP016399 | Strain FORC_040 plasmid unnamed1 | ABS | N/A | ABS | NCBI |
| 336 | NZ_CP016854 | Subsp. *aureus* Strain 5118.N plasmid p5118.Nb | ABS | N/A | ABS | NCBI |
| 337 | NZ_CP016855 | Subsp. *aureus* Strain 5118.N | ABS | N/A | ABS | NCBI |
| 338 | NZ_CP016856 | Subsp. *aureus* Strain 2148.N | ABS | N/A | ABS | NCBI |
| 339 | NZ_CP016857 | Subsp. *aureus* Strain 1971.C01 plasmid p1971.C01c | ABS | N/A | ABS | NCBI |
| 340 | NZ_CP016858 | Subsp. *aureus* Strain 1971.C01 | ABS | N/A | ABS | NCBI |
| 341 | NZ_CP016859 | Subsp. *aureus* Strain 1971.C01 plasmid p1971.C01b | ABS | N/A | ABS | NCBI |
| 342 | NZ_CP016860 | Subsp. *aureus* Strain 1969.N plasmid p1969.N | ABS | N/A | ABS | NCBI |
| 343 | NZ_CP016861 | Subsp. *aureus* Strain 1969.N | ABS | N/A | ABS | NCBI |
| 344 | NZ_CP016862 | Subsp. *aureus* Strain 1625.C01 plasmid p1625.C01 | ABS | N/A | ABS | NCBI |
| 345 | NZ_CP016863 | Subsp. *aureus* Strain 1625.C01 | ABS | N/A | ABS | NCBI |
| N/A: No applique, ABS: Absent, NCBI: National Center for Biotechnology Information, ENA: European Nucleotide Archive, *Evidence level 4 and 3, *cas* genes | | | | | | |
| **Supplementary table A. Table of CRISPR-Cas system- and CRISPR-Cas system non-bearing strains of *Staphylococcus aureus* (continuation).** | | | | | | |
| **#** | **ACCESSION NUMBER** | **DESCRIPTION** | **CRISPR-Cas system** | **EVIDENCE LEVEL** | ***cas* GENES** | **DATABASE** |
| 346 | NZ_CP017090 | Strain ISU935 | ABS | N/A | ABS | NCBI |
| 347 | NZ_CP017091 | Subsp. *aureus* Strain ISU926 | ABS | N/A | ABS | NCBI |
| 348 | NZ_CP017094 | Subsp. *aureus* Strain 2148.C01 | ABS | N/A | ABS | NCBI |
| 349 | NZ_CP017095 | Subsp. *aureus* Strain 2148.C01 plasmid p2148.CO1_B | ABS | N/A | ABS | NCBI |
| 350 | NZ_CP017115 | Strain FORC_045 | ABS | N/A | ABS | NCBI |
| 351 | NZ_CP017677 | Strain CFSAN007894 | ABS | N/A | ABS | NCBI |
| 352 | NZ_CP017678 | Strain CFSAN007894 plasmid pCFSAN007894 | ABS | N/A | ABS | NCBI |
| 353 | NZ_CP017679 | Strain CFSAN007883 | ABS | N/A | ABS | NCBI |
| 354 | NZ_CP017680 | Strain CFSAN007851 | ABS | N/A | ABS | NCBI |
| 355 | NZ_CP017681 | Strain CFSAN007851 plasmid pCFSAN007851 | ABS | N/A | ABS | NCBI |
| 356 | NZ_CP017682 | Strain CFSAN007850 | ABS | N/A | ABS | NCBI |
| 357 | NZ_CP017683 | Strain CFSAN007850 plasmid pCFSAN007850 | ABS | N/A | ABS | NCBI |
| 358 | NZ_CP017684 | Strain CFSAN007847 | ABS | N/A | ABS | NCBI |
| 359 | NZ_CP017685 | Strain CFSAN007835 | ABS | N/A | ABS | NCBI |
| 360 | NZ_CP018205 | Subsp. *aureus* Strain HG001 | ABS | N/A | ABS | NCBI |
| 361 | NZ_CP018629 | Strain MRSA107 | ABS | N/A | ABS | NCBI |
| 362 | NZ_CP018766 | Subsp. *aureus* Strain UCI62 | ABS | N/A | ABS | NCBI |
| 363 | NZ_CP018767 | Subsp. *aureus* Strain UCI62 plasmid pUCI62 | ABS | N/A | ABS | NCBI |
| 364 | NZ_CP018768 | Subsp. *aureus* Strain UCI 28 | ABS | N/A | ABS | NCBI |
| 365 | NZ_CP018769 | Subsp. *aureus* Strain UCI 28 plasmid pUCI28 | ABS | N/A | ABS | NCBI |
| 366 | NZ_CP019117 | Strain SJTUF_J27 | ABS | N/A | ABS | NCBI |
| 367 | NZ_CP019563 | Strain SR434 | ABS | N/A | ABS | NCBI |
| 368 | NZ_CP019564 | Strain SR434 plasmid pSR01 | ABS | N/A | ABS | NCBI |
| N/A: No applique, ABS: Absent, NCBI: National Center for Biotechnology Information, ENA: European Nucleotide Archive, *Evidence level 4 and 3, *cas* genes | | | | | | |
| **Supplementary table A. Table of CRISPR-Cas system- and CRISPR-Cas system non-bearing strains of *Staphylococcus aureus* (continuation).** | | | | | | |
| **#** | **ACCESSION NUMBER** | **DESCRIPTION** | **CRISPR-Cas system** | **EVIDENCE LEVEL** | ***cas* GENES** | **DATABASE** |
| 369 | NZ_CP019565 | Strain SR434 plasmid pSR02 | ABS | N/A | ABS | NCBI |
| 370 | NZ_CP019566 | Strain SR434 plasmid pSR03 | ABS | N/A | ABS | NCBI |
| 371 | NZ_CP019567 | Strain SR434 plasmid pSR04 | ABS | N/A | ABS | NCBI |
| 372 | NZ_CP019574 | Strain USA400-0051 | ABS | N/A | ABS | NCBI |
| 373 | NZ_CP019575 | Strain USA400-0051 plasmid unnamed | ABS | N/A | ABS | NCBI |
| 374 | NZ_CP019590 | Strain C2406 | ABS | N/A | ABS | NCBI |
| 375 | NZ_CP019591 | Strain 293G | ABS | N/A | ABS | NCBI |
| 376 | NZ_CP019593 | Strain GD705 | ABS | N/A | ABS | NCBI |
| 377 | NZ_CP019594 | Strain GD1539 | ABS | N/A | ABS | NCBI |
| 378 | NZ_CP019595 | Strain GD1677 | ABS | N/A | ABS | NCBI |
| 379 | NZ_CP019945 | Strain BA01611 | ABS | N/A | ABS | NCBI |
| 380 | NZ_CP020019 | Strain 08S00974 | ABS | N/A | ABS | NCBI |
| 381 | NZ_CP020020 | Subsp. *aureus* Strain ATCC 6538 | ABS | N/A | ABS | NCBI |
| 382 | NZ_CP020021 | Subsp. *aureus* Strain ATCC 6538 plasmid unnamed1 | ABS | N/A | ABS | NCBI |
| 383 | NZ_CP020354 | Strain FORC59 | ABS | N/A | ABS | NCBI |
| 384 | NZ_CP020355 | Strain FORC59 plasmid pFORC59 | ABS | N/A | ABS | NCBI |
| 385 | NZ_CP020467 | Strain CFSAN007896 | ABS | N/A | ABS | NCBI |
| 386 | NZ_CP020619 | Strain JE2 | ABS | N/A | ABS | NCBI |
| 387 | NZ_CP020656 | Strain K5 | ABS | N/A | ABS | NCBI |
| 388 | NZ_CP020713 | Strain K17 | ABS | N/A | ABS | NCBI |
| 389 | NZ_CP020714 | Strain K18 | ABS | N/A | ABS | NCBI |
| 390 | NZ_CP020741 | Strain HZW450 | ABS | N/A | ABS | NCBI |
| 391 | NZ_CP020956 | Strain C8879 | ABS | N/A | ABS | NCBI |
| N/A: No applique, ABS: Absent, NCBI: National Center for Biotechnology Information, ENA: European Nucleotide Archive, *Evidence level 4 and 3, *cas* genes | | | | | | |
| **Supplementary table A. Table of CRISPR-Cas system- and CRISPR-Cas system non-bearing strains of *Staphylococcus aureus* (continuation).** | | | | | | |
| **#** | **ACCESSION NUMBER** | **DESCRIPTION** | **CRISPR-Cas system** | **EVIDENCE LEVEL** | ***cas* GENES** | **DATABASE** |
| 392 | NZ_CP020957 | Strain C3948 | ABS | N/A | ABS | NCBI |
| 393 | NZ_CP020959 | Strain H489 | ABS | N/A | ABS | NCBI |
| 394 | NZ_CP020960 | Strain JK3137 | ABS | N/A | ABS | NCBI |
| 395 | NZ_CP021105 | Strain CC5 | ABS | N/A | ABS | NCBI |
| 396 | NZ_CP021171 | Strain CHU15-056 | ABS | N/A | ABS | NCBI |
| 397 | NZ_CP021905 | Strain Seattle 1945 isolate G477 | ABS | N/A | ABS | NCBI |
| 398 | NZ_CP021906 | Strain Seattle 1945 isolate G477 plasmid pG477 | ABS | N/A | ABS | NCBI |
| 399 | NZ_CP021907 | Strain Seattler 1945 isolate G478 | ABS | N/A | ABS | NCBI |
| 400 | NZ_CP021908 | Strain Seattle 1945 isolate G478 plasmid pG478 | ABS | N/A | ABS | NCBI |
| 401 | NZ_CP022290 | Subsp. *aureus* EDCC5458 | ABS | N/A | ABS | NCBI |
| 402 | NZ_CP022291 | Subsp. *aureus* EDCC5464 | ABS | N/A | ABS | NCBI |
| 403 | NZ_CP022582 | Strain FORC 062 | ABS | N/A | ABS | NCBI |
| 404 | NZ_CP022607 | Strain FORC 061 | ABS | N/A | ABS | NCBI |
| 405 | NZ_CP022608 | Strain FORC_061 plasmid pFORC61_2 | ABS | N/A | ABS | NCBI |
| 406 | NZ_CP022682 | Strain 78 | ABS | N/A | ABS | NCBI |
| 407 | NZ_CP022683 | Strain 78 plasmid pPS00085.1A.1 | ABS | N/A | ABS | NCBI |
| 408 | NZ_CP022717 | Strain 27 | ABS | N/A | ABS | NCBI |
| 409 | NZ_CP022718 | Strain 45 | ABS | N/A | ABS | NCBI |
| 410 | NZ_CP022719 | Strain 45 plasmid pPS00086.1A.1 | ABS | N/A | ABS | NCBI |
| 411 | NZ_CP022720 | Strain 135 | ABS | N/A | ABS | NCBI |
| 412 | NZ_CP022721 | Strain 135 plasmid pPS00087.1A.1 | ABS | N/A | ABS | NCBI |
| 413 | NZ_CP022892 | Strain 54 | ABS | N/A | ABS | NCBI |
| 414 | NZ_CP022893 | Strain 61 | ABS | N/A | ABS | NCBI |
| N/A: No applique, ABS: Absent, NCBI: National Center for Biotechnology Information, ENA: European Nucleotide Archive, *Evidence level 4 and 3, *cas* genes | | | | | | |
| **Supplementary table A. Table of CRISPR-Cas system- and CRISPR-Cas system non-bearing strains of *Staphylococcus aureus* (continuation).** | | | | | | |
| **#** | **ACCESSION NUMBER** | **DESCRIPTION** | **CRISPR-Cas system** | **EVIDENCE LEVEL** | ***cas* GENES** | **DATABASE** |
| 415 | NZ_CP022894 | Strain 191 | ABS | N/A | ABS | NCBI |
| 416 | NZ_CP022895 | Strain 85 | ABS | N/A | ABS | NCBI |
| 417 | NZ_CP022896 | Strain 277 | ABS | N/A | ABS | NCBI |
| 418 | NZ_CP022897 | Strain 128 | ABS | N/A | ABS | NCBI |
| 419 | NZ_CP022898 | Strain 422 | ABS | N/A | ABS | NCBI |
| 420 | NZ_CP022899 | Strain 143 | ABS | N/A | ABS | NCBI |
| 421 | NZ_CP022900 | Strain 468 | ABS | N/A | ABS | NCBI |
| 422 | NZ_CP022901 | Strain 466 | ABS | N/A | ABS | NCBI |
| 423 | NZ_CP022902 | Strain 165 | ABS | N/A | ABS | NCBI |
| 424 | NZ_CP022903 | Strain 187 | ABS | N/A | ABS | NCBI |
| 425 | NZ_CP022904 | Strain 629 | ABS | N/A | ABS | NCBI |
| 426 | NZ_CP022905 | Strain 628 | ABS | N/A | ABS | NCBI |
| 427 | NZ_CP022906 | Strain 546 | ABS | N/A | ABS | NCBI |
| 428 | NZ_CP022907 | Strain 546 plasmid pPS00077.1A.1 | ABS | N/A | ABS | NCBI |
| 429 | NZ_CP022908 | Strain 545 | ABS | N/A | ABS | NCBI |
| 430 | NZ_CP022909 | Strain 545 plasmid pPS00078.1A.1 | ABS | N/A | ABS | NCBI |
| 431 | NZ_CP022910 | Strain 164 | ABS | N/A | ABS | NCBI |
| 432 | NZ_CP022911 | Strain 164 plasmid pPS00089.1A.1 | ABS | N/A | ABS | NCBI |
| 433 | NZ_CP023390 | Subsp. *aureus* Strain Newman | ABS | N/A | ABS | NCBI |
| 434 | NZ_CP023391 | Subsp. *aureus* Strain Newman | ABS | N/A | ABS | NCBI |
| 435 | NZ_CP023500 | Strain FDAARGOS_412 | ABS | N/A | ABS | NCBI |
| 436 | NZ_CP023561 | Strain TF3198 | ABS | N/A | ABS | NCBI |
| 437 | NZ_CP023562 | Strain TF3198 plasmid pTF3198 | ABS | N/A | ABS | NCBI |
| N/A: No applique, ABS: Absent, NCBI: National Center for Biotechnology Information, ENA: European Nucleotide Archive, *Evidence level 4 and 3, *cas* genes | | | | | | |
| **Supplementary table A. Table of CRISPR-Cas system- and CRISPR-Cas system non-bearing strains of *Staphylococcus aureus* (continuation).** | | | | | | |
| **#** | **ACCESSION NUMBER** | **DESCRIPTION** | **CRISPR-Cas system** | **EVIDENCE LEVEL** | ***cas* GENES** | **DATABASE** |
| 438 | NZ_CP024998 | Strain 55-99-44 | ABS | N/A | ABS | NCBI |
| 439 | NZ_CP024999 | Strain 55-99-44 plasmid punamed1 | ABS | N/A | ABS | NCBI |
| 440 | NZ_CP025395 | Strain O46 | ABS | N/A | ABS | NCBI |
| 441 | NZ_CP025481 | Subsp. *aureus* Strain 2148.N plasmid p2148.N | ABS | N/A | ABS | NCBI |
| 442 | NZ_CP025482 | Subsp. *aureus* Strain 5118.N plasmid p5118.N_A | ABS | N/A | ABS | NCBI |
| 443 | NZ_CP025486 | Subsp. *aureus* Strain 1971.C01 plasmid p1971.C01_A | ABS | N/A | ABS | NCBI |
| 444 | NZ_CP025487 | Subsp. *aureus* Strain 1969.N plasmid p1969.N_A | ABS | N/A | ABS | NCBI |
| 445 | NZ_CP025488 | Subsp. *aureus* Strain 2148.C01 plasmid p2148.C01_A | ABS | N/A | ABS | NCBI |
| 446 | NZ_CP025489 | Subsp. *aureus* Strain USA300_2014.C02 plasmid pC02_A | ABS | N/A | ABS | NCBI |
| 447 | NZ_CP025490 | Subsp. *aureus* Strain USA300_2014.C01 plasmid pC01_A | ABS | N/A | ABS | NCBI |
| 448 | NZ_CP025495 | Subsp. *aureus* Strain 3020_C01 | ABS | N/A | ABS | NCBI |
| 449 | NZ_CP025496 | Subsp. *aureus* Strain 3020.C01 plasmid p3020.C01a | ABS | N/A | ABS | NCBI |
| 450 | NZ_CP025497 | Subsp. *aureus* Strain 3020.C01 plasmid p3020.C01b | ABS | N/A | ABS | NCBI |
| 451 | NZ_CP026063 | Strain NRS149 | ABS | N/A | ABS | NCBI |
| 452 | NZ_CP026064 | Strain NRS271 | ABS | N/A | ABS | NCBI |
| 453 | NZ_CP026065 | Strain NRS271 plasmid unnamed | ABS | N/A | ABS | NCBI |
| 454 | NZ_CP026066 | Strain NRS484 | ABS | N/A | ABS | NCBI |
| 455 | NZ_CP026067 | Strain NRS153 | ABS | N/A | ABS | NCBI |
| 456 | NZ_CP026068 | Strain NRS146 | ABS | N/A | ABS | NCBI |
| 457 | NZ_CP026069 | Strain NRS1 | ABS | N/A | ABS | NCBI |
| 458 | NZ_CP026070 | Strain NRS133 | ABS | N/A | ABS | NCBI |
| 459 | NZ_CP026071 | Strain NRS143 | ABS | N/A | ABS | NCBI |
| 460 | NZ_CP026072 | Strain NRS120 | ABS | N/A | ABS | NCBI |
| N/A: No applique, ABS: Absent, NCBI: National Center for Biotechnology Information, ENA: European Nucleotide Archive, *Evidence level 4 and 3, *cas* genes | | | | | | |
| **Supplementary table A. Table of CRISPR-Cas system- and CRISPR-Cas system non-bearing strains of *Staphylococcus aureus* (continuation).** | | | | | | |
| **#** | **ACCESSION NUMBER** | **DESCRIPTION** | **CRISPR-Cas system** | **EVIDENCE LEVEL** | ***cas* GENES** | **DATABASE** |
| 461 | NZ_CP026073 | Strain Mw2 | ABS | N/A | ABS | NCBI |
| 462 | NZ_CP026074 | Strain HPV107 | ABS | N/A | ABS | NCBI |
| 463 | NZ_CP026075 | Strain HPB107 plasmid unnamed | ABS | N/A | ABS | NCBI |
| 464 | NZ_CP026076 | Strain TCH 959 | ABS | N/A | ABS | NCBI |
| 465 | NZ_CP026077 | Strain NRS107 | ABS | N/A | ABS | NCBI |
| 466 | NZ_CP026078 | Strain NRS107 plasmid unnamed | ABS | N/A | ABS | NCBI |
| 467 | NZ_CP026079 | Strain NRS70 | ABS | N/A | ABS | NCBI |
| 468 | NZ_CP026080 | Strain NRS137 | ABS | N/A | ABS | NCBI |
| 469 | NZ_CP026081 | Strain NRS137 plasmid unnamed | ABS | N/A | ABS | NCBI |
| 470 | NZ_CP026646 | Strain 2288 | ABS | N/A | ABS | NCBI |
| 471 | NZ_CP026953 | Strain FDAARGOS_48 | ABS | N/A | ABS | NCBI |
| 472 | NZ_CP026954 | Strain FDAARGOS_48 plasmid unnamed1 | ABS | N/A | ABS | NCBI |
| 473 | NZ_CP026955 | Strain FDAARGOS_48 plasmid unnamed2 | ABS | N/A | ABS | NCBI |
| 474 | NZ_CP026956 | Strain FDAARGOS_48 plasmid unnamed3 | ABS | N/A | ABS | NCBI |
| 475 | NZ_CP026957 | Strain FDAARGOS_43 | ABS | N/A | ABS | NCBI |
| 476 | NZ_CP026958 | Strain FDAARGOS_40 | ABS | N/A | ABS | NCBI |
| 477 | NZ_CP026959 | Strain FDAARGOS_40 plasmid unnamed1 | ABS | N/A | ABS | NCBI |
| 478 | NZ_CP026960 | Strain FDAARGOS_15 | ABS | N/A | ABS | NCBI |
| 479 | NZ_CP026961 | Strain FDAARGOS_10 | ABS | N/A | ABS | NCBI |
| 480 | NZ_CP026962 | Strain FDAARGOS_6 | ABS | N/A | ABS | NCBI |
| 481 | NZ_CP026963 | Strain FDAARGOS_6 plasmid unnamed1 | ABS | N/A | ABS | NCBI |
| 482 | NZ_CP026964 | Strain FDAARGOS_2 | ABS | N/A | ABS | NCBI |
| 483 | NZ_CP026965 | Strain FDAARGOS_2 plasmid unnamed1 | ABS | N/A | ABS | NCBI |
| N/A: No applique, ABS: Absent, NCBI: National Center for Biotechnology Information, ENA: European Nucleotide Archive, *Evidence level 4 and 3, *cas* genes | | | | | | |
| **Supplementary table A. Table of CRISPR-Cas system- and CRISPR-Cas system non-bearing strains of *Staphylococcus aureus* (continuation).** | | | | | | |
| **#** | **ACCESSION NUMBER** | **DESCRIPTION** | **CRISPR-Cas system** | **EVIDENCE LEVEL** | ***cas* GENES** | **DATABASE** |
| 484 | NZ_CP026966 | Strain FDAARGOS_2 plasmid unnamed2 | ABS | N/A | ABS | NCBI |
| 485 | NZ_CP026967 | Strain FDAARGOS_2 plasmid unnamed3 | ABS | N/A | ABS | NCBI |
| 486 | NZ_CP026968 | Strain FDAARGOS_1 | ABS | N/A | ABS | NCBI |
| 487 | NZ_CP026969 | Strain FDAARGOS_1 plasmid unnamed1 | ABS | N/A | ABS | NCBI |
| 488 | NZ_CP026970 | Strain FDAARGOS_1 plasmid unnamed2 | ABS | N/A | ABS | NCBI |
| 489 | NZ_CP026971 | Strain FDAARGOS_1 plasmid unnamed3 | ABS | N/A | ABS | NCBI |
| 490 | NZ_CP026972 | Strain FDAARGOS_1 plasmid unnamed4 | ABS | N/A | ABS | NCBI |
| 491 | NZ_CP027788 | Strain CMRSA-6 | ABS | N/A | ABS | NCBI |
| 492 | NZ_CP028163 | Strain CFSAN064038 | ABS | N/A | ABS | NCBI |
| 493 | NZ_CP028164 | Strain CFSAN064038 plasmid pGMI17-006 | ABS | N/A | ABS | NCBI |
| 494 | NZ_CP028165 | Strain CFSAN064037 | ABS | N/A | ABS | NCBI |
| 495 | NZ_CP028189 | Strain CFSAN018750 | ABS | N/A | ABS | NCBI |
| 496 | NZ_CP028190 | Strain CFSAN018749 | ABS | N/A | ABS | NCBI |
| 497 | NZ_CP028191 | Strain CFSAN018749 plasmid pGMI14-005 | ABS | N/A | ABS | NCBI |
| 498 | NZ_CP028468 | Strain IT1-S | ABS | N/A | ABS | NCBI |
| 499 | NZ_CP028469 | Strain IT1-S plasmid pIT1-S | ABS | N/A | ABS | NCBI |
| 500 | NZ_CP028470 | Strain IT4-R | ABS | N/A | ABS | NCBI |
| 501 | NZ_CP028471 | Strain IT4-R plasmid pIT4-R | ABS | N/A | ABS | NCBI |
| 502 | NZ_CP029030 | Subsp. *aureus* Strain CAR | ABS | N/A | ABS | NCBI |
| 503 | NZ_CP029031 | Subsp. *aureus* Strain CIT | ABS | N/A | ABS | NCBI |
| 504 | NZ_CP029032 | Subsp. *aureus* Strain OXLIM | ABS | N/A | ABS | NCBI |
| 505 | NZ_CP029079 | Strain AR466 plasmid unnamed1 | ABS | N/A | ABS | NCBI |
| 506 | NZ_CP029080 | Strain AR466 | ABS | N/A | ABS | NCBI |
| N/A: No applique, ABS: Absent, NCBI: National Center for Biotechnology Information, ENA: European Nucleotide Archive, *Evidence level 4 and 3, *cas* genes | | | | | | |
| **Supplementary table A. Table of CRISPR-Cas system- and CRISPR-Cas system non-bearing strains of *Staphylococcus aureus* (continuation).** | | | | | | |
| **#** | **ACCESSION NUMBER** | **DESCRIPTION** | **CRISPR-Cas system** | **EVIDENCE LEVEL** | ***cas* GENES** | **DATABASE** |
| 507 | NZ_CP029081 | Strain AR465 plasmid nnamed1 | ABS | N/A | ABS | NCBI |
| 508 | NZ_CP029082 | Strain AR465 | ABS | N/A | ABS | NCBI |
| 509 | NZ_CP029083 | Strain AR464 plasmid unnamed1 | ABS | N/A | ABS | NCBI |
| 510 | NZ_CP029084 | Strain AR464 | ABS | N/A | ABS | NCBI |
| 511 | NZ_CP029085 | Strain AR462 plasmid unnamed1 | ABS | N/A | ABS | NCBI |
| 512 | NZ_CP029086 | Strain AR462 | ABS | N/A | ABS | NCBI |
| 513 | NZ_CP029087 | Strain AR461 | ABS | N/A | ABS | NCBI |
| 514 | NZ_CP029165 | Strain SVH7513 plasmid pSVH7513b | ABS | N/A | ABS | NCBI |
| 515 | NZ_CP029166 | Strain SVH7513 | ABS | N/A | ABS | NCBI |
| 516 | NZ_CP029167 | Strain SVH7513 plasmid pSVH7513a | ABS | N/A | ABS | NCBI |
| 517 | NZ_CP029168 | Strain PTDrAP2 plasmid pPTDrAP2a | ABS | N/A | ABS | NCBI |
| 518 | NZ_CP029169 | Strain PTDrAP2 plasmid pPTDrAP2b | ABS | N/A | ABS | NCBI |
| 519 | NZ_CP029170 | Strain PTDrAP2 plasmid pPTDrAP2c | ABS | N/A | ABS | NCBI |
| 520 | NZ_CP029171 | Strain PTDrAP2 plasmid pPTDrAP2d | ABS | N/A | ABS | NCBI |
| 521 | NZ_CP029172 | Strain PTDrAP2 | ABS | N/A | ABS | NCBI |
| 522 | NZ_CP029198 | Strain *aureus* | ABS | N/A | ABS | NCBI |
| 523 | NZ_CP029199 | Strain *aureus* plasmid pFORC_090.1 | ABS | N/A | ABS | NCBI |
| 524 | NZ_CP029474 | Strain USA 100 isolate 30-47 | ABS | N/A | ABS | NCBI |
| 525 | NZ_CP029475 | Strain USA 100 isolate 30-47 plasmid unnamed | ABS | N/A | ABS | NCBI |
| 526 | NZ_CP029627 | Strain MOK042 | ABS | N/A | ABS | NCBI |
| 527 | NZ_CP029628 | Strain MOK042 plasmid unnamed | ABS | N/A | ABS | NCBI |
| 528 | NZ_CP029629 | Strain MOK63 | ABS | N/A | ABS | NCBI |
| 529 | NZ_CP029648 | Strain AR_0472 plasmid unnamed1 | ABS | N/A | ABS | NCBI |
| N/A: No applique, ABS: Absent, NCBI: National Center for Biotechnology Information, ENA: European Nucleotide Archive, *Evidence level 4 and 3, *cas* genes | | | | | | |
| **Supplementary table A. Table of CRISPR-Cas system- and CRISPR-Cas system non-bearing strains of *Staphylococcus aureus* (continuation).** | | | | | | |
| **#** | **ACCESSION NUMBER** | **DESCRIPTION** | **CRISPR-Cas system** | **EVIDENCE LEVEL** | ***cas* GENES** | **DATABASE** |
| 530 | NZ_CP029649 | Strain AR_0472 | III-A | 4 | *cas6, csm6, csm5, csm4, csm3, csm2, cas10, cas2, cas1* | NCBI |
| 531 | NZ_CP029650 | Strain AR_0471 plasmid unnamed1 | ABS | N/A | ABS | NCBI |
| 532 | NZ_CP029651 | Strain AR_0471 plasmid unnamed2 | ABS | N/A | ABS | NCBI |
| 533 | NZ_CP029652 | Strain AR_0471 | ABS | N/A | ABS | NCBI |
| 534 | NZ_CP029653 | Strain AR_0470 | III-A | 4 | *cas1, cas2, cas10, csm2, csm3, csm4, csm5, csm6, cas6* | NCBI |
| 535 | NZ_CP029654 | Strain AR_0469 plasmid unnamed1 | ABS | N/A | ABS | NCBI |
| 536 | NZ_CP029655 | Strain AR_0469 | ABS | N/A | ABS | NCBI |
| 537 | NZ_CP029656 | Strain AR_0468 plasmid unnamed1 | ABS | N/A | ABS | NCBI |
| 538 | NZ_CP029657 | Strain AR_0468 | ABS | N/A | ABS | NCBI |
| 539 | NZ_CP029658 | Strain AR_0467 | ABS | N/A | ABS | NCBI |
| 540 | NZ_CP029659 | Strain AR_0467 plasmid unnamed1 | ABS | N/A | ABS | NCBI |
| 541 | NZ_CP029661 | Strain AR_0228 plasmid unnamed1 | ABS | N/A | ABS | NCBI |
| 542 | NZ_CP029662 | Strain AR_0228 plasmid unnamed2 | ABS | N/A | ABS | NCBI |
| 543 | NZ_CP029663 | Strain AR_0228 | ABS | N/A | ABS | NCBI |
| 544 | NZ_CP029664 | Strain AR_0226 | ABS | N/A | ABS | NCBI |
| 545 | NZ_CP029665 | Strain AR_0226 plasmid unnamed1 | ABS | N/A | ABS | NCBI |
| 546 | NZ_CP029666 | Strain AR_0225 plasmid unnamed1 | ABS | N/A | ABS | NCBI |
| 547 | NZ_CP029667 | Strain AR_0225 | ABS | N/A | ABS | NCBI |
| 548 | NZ_CP029668 | Strain AR_0223 plasmid unnamed1 | ABS | N/A | ABS | NCBI |
| 549 | NZ_CP029669 | Strain AR_0223 | ABS | N/A | ABS | NCBI |
| 550 | NZ_CP029670 | Strain AR_0222 plasmid unnamed1 | ABS | N/A | ABS | NCBI |
| 551 | NZ_CP029671 | Strain AR_0222 | ABS | N/A | ABS | NCBI |
| 552 | NZ_CP029672 | Strain AR_220 plasmid unnamed1 | ABS | N/A | ABS | NCBI |
| N/A: No applique, ABS: Absent, NCBI: National Center for Biotechnology Information, ENA: European Nucleotide Archive, *Evidence level 4 and 3, *cas* genes | | | | | | |
| **Supplementary table A. Table of CRISPR-Cas system- and CRISPR-Cas system non-bearing strains of *Staphylococcus aureus* (continuation).** | | | | | | |
| **#** | **ACCESSION NUMBER** | **DESCRIPTION** | **CRISPR-Cas system** | **EVIDENCE LEVEL** | ***cas* GENES** | **DATABASE** |
| 553 | NZ_CP029673 | Strain AR_0220 | ABS | N/A | ABS | NCBI |
| 554 | NZ_CP029674 | Strain AR_0219 plasmid unnamed1 | ABS | N/A | ABS | NCBI |
| 555 | NZ_CP029675 | Strain AR_0219 | ABS | N/A | ABS | NCBI |
| 556 | NZ_CP029676 | Strain AR_0216 plasmid unnamed 1 | ABS | N/A | ABS | NCBI |
| 557 | NZ_CP029677 | Strain AR_0216 plasmid unnamed2 | ABS | N/A | ABS | NCBI |
| 558 | NZ_CP029678 | Strain AR_0216 | ABS | N/A | ABS | NCBI |
| 559 | NZ_CP029679 | Strain AR_0215 plasmid unnamed1 | ABS | N/A | ABS | NCBI |
| 560 | NZ_CP029680 | Strain AR_0215 | ABS | N/A | ABS | NCBI |
| 561 | NZ_CP029681 | Strain AR_0473 | III-A | 4 | *cas6, csm6, csm5, csm4, csm3, csm2, cas10, cas2, cas1* | NCBI |
| 562 | NZ_CP029685 | Strain CMRSA-3 | ABS | N/A | ABS | NCBI |
| 563 | NZ_CP030136 | Strain S57 | ABS | N/A | ABS | NCBI |
| 564 | NZ_CP030137 | Strain M51 | ABS | N/A | ABS | NCBI |
| 565 | NZ_CP039138 | Strain M48 | ABS | N/A | ABS | NCBI |
| 566 | NZ_CP030323 | Strain AR_475 | ABS | N/A | ABS | NCBI |
| 567 | NZ_CP030324 | Strain AR_475 plasmid unnamed1 | ABS | N/A | ABS | NCBI |
| 568 | NZ_CP030325 | Strain AR_474 plasmid unnamed1 | ABS | N/A | ABS | NCBI |
| 569 | NZ_CP030326 | Strain AR_474 | ABS | N/A | ABS | NCBI |
| 570 | NZ_CP031130 | Strain F17SA003 | ABS | N/A | ABS | NCBI |
| 571 | NZ_CP031131 | Strain E16SA093 | ABS | N/A | ABS | NCBI |
| 572 | NZ_CP031265 | Strain 13 | ABS | N/A | ABS | NCBI |
| 573 | NZ_CP031537 | Strain WCH-SK2 | ABS | N/A | ABS | NCBI |
| 574 | NZ_CP031661 | Strain 82 | ABS | N/A | ABS | NCBI |
| 575 | NZ_CP031662 | Strain 82 plasmid pPS00051.1A.1 | ABS | N/A | ABS | NCBI |
| N/A: No applique, ABS: Absent, NCBI: National Center for Biotechnology Information, ENA: European Nucleotide Archive, *Evidence level 4 and 3, *cas* genes | | | | | | |
| **Supplementary table A. Table of CRISPR-Cas system- and CRISPR-Cas system non-bearing strains of *Staphylococcus aureus* (continuation).** | | | | | | |
| **#** | **ACCESSION NUMBER** | **DESCRIPTION** | **CRISPR-Cas system** | **EVIDENCE LEVEL** | ***cas* GENES** | **DATABASE** |
| 576 | NZ_CP031663 | Strain 82 plasmid pPS00051.1A.2 | ABS | N/A | ABS | NCBI |
| 577 | NZ_CP031664 | Strain 28 | ABS | N/A | ABS | NCBI |
| 578 | NZ_CP031665 | Strain 28 plasmid pPS00026.1A.1 | ABS | N/A | ABS | NCBI |
| 579 | NZ_CP031666 | Strain 28 plasmid pPS00026.1A.2 | ABS | N/A | ABS | NCBI |
| 580 | NZ_CP031667 | Strain 199 | ABS | N/A | ABS | NCBI |
| 581 | NZ_CP031668 | Strain 199 plasmid pPS00120.1A.1 | ABS | N/A | ABS | NCBI |
| 582 | NZ_CP031669 | Strain 199 plasmid pPS00120.1A.2 | ABS | N/A | ABS | NCBI |
| 583 | NZ_CP031670 | Strain 64 | ABS | N/A | ABS | NCBI |
| 584 | NZ_CP031671 | Strain 64 plasmid pPS00119.1A.1 | ABS | N/A | ABS | NCBI |
| 585 | NZ_CP031672 | Strain 64 plasmid pP200119.1A.2 | ABS | N/A | ABS | NCBI |
| 586 | NZ_CP031673 | Strain MOZ66 | ABS | N/A | ABS | NCBI |
| 587 | NZ_CP031779 | Strain CFBR-105 | ABS | N/A | ABS | NCBI |
| 588 | NZ_CP031838 | Strain QD-CD9 | ABS | N/A | ABS | NCBI |
| 589 | NZ_CP031839 | Strain NX-T55 | ABS | N/A | ABS | NCBI |
| 590 | NZ_CP031840 | Strain NX-T55 plasmid unnamed | ABS | N/A | ABS | NCBI |
| 591 | NZ_CP031886 | Strain CFSAN082783 | ABS | N/A | ABS | NCBI |
| 592 | NZ_CP031887 | Strain CFSAN082783 plasmid pMRSA_24 | ABS | N/A | ABS | NCBI |
| 593 | NZ_CP031888 | Strain CFSAN082782 | ABS | N/A | ABS | NCBI |
| 594 | NZ_CP031889 | Strain CFSAN082782 plasmid pMRSA_23 | ABS | N/A | ABS | NCBI |
| 595 | NZ_CP031890 | Strain CFSAN082781 | ABS | N/A | ABS | NCBI |
| 596 | NZ_CP031891 | Strain CFSAN082781 plasmid pMRSA_22 | ABS | N/A | ABS | NCBI |
| 597 | NZ_CP032051 | Strain O17 | ABS | N/A | ABS | NCBI |
| 598 | NZ_CP032481 | Strain O326 | ABS | N/A | ABS | NCBI |
| N/A: No applique, ABS: Absent, NCBI: National Center for Biotechnology Information, ENA: European Nucleotide Archive, *Evidence level 4 and 3, *cas* genes | | | | | | |
| **Supplementary table A. Table of CRISPR-Cas system- and CRISPR-Cas system non-bearing strains of *Staphylococcus aureus* (continuation).** | | | | | | |
| **#** | **ACCESSION NUMBER** | **DESCRIPTION** | **CRISPR-Cas system** | **EVIDENCE LEVEL** | ***cas* GENES** | **DATABASE** |
| 599 | NZ_CP033112 | Strain ST20130944 | ABS | N/A | ABS | NCBI |
| 600 | NZ_CP033113 | Strain ST20130944 plasmid pST20130944 | ABS | N/A | ABS | NCBI |
| 601 | NZ_CP033114 | Strain ST20130945 | ABS | N/A | ABS | NCBI |
| 602 | NZ_CP033115 | Strain ST20130945 plasmid pST20130945 | ABS | N/A | ABS | NCBI |
| 603 | NZ_CP033505 | Strain ATCC BAA-39 | ABS | N/A | ABS | NCBI |
| 604 | NZ_CP033865 | Strain FDAARGOS_504 | ABS | N/A | ABS | NCBI |
| 605 | NZ_CP033973 | Strain P2D15C1 plasmid unnamed1 | ABS | N/A | ABS | NCBI |
| 606 | NZ_CP033974 | Strain P2D15C1 plasmid unnamed2 | ABS | N/A | ABS | NCBI |
| 607 | NZ_CP033975 | Strain P2D15C1 plasmid unnamed3 | ABS | N/A | ABS | NCBI |
| 608 | NZ_CP033976 | Strain P2D15C1 plasmid unnamed4 | ABS | N/A | ABS | NCBI |
| 609 | NZ_CP033977 | Strain P2D15C1 | ABS | N/A | ABS | NCBI |
| 610 | NZ_CP033978 | Strain P2D8C1 plasmid unnamed1 | ABS | N/A | ABS | NCBI |
| 611 | NZ_CP033979 | Strain P2D8C1 plasmid unnamed2 | ABS | N/A | ABS | NCBI |
| 612 | NZ_CP033980 | Strain P2D8C1 plasmid unnamed3 | ABS | N/A | ABS | NCBI |
| 613 | NZ_CP033981 | Strain P2D8C1 plasmid unnamed4 | ABS | N/A | ABS | NCBI |
| 614 | NZ_CP033982 | Strain P2D8C1 | ABS | N/A | ABS | NCBI |
| 615 | NZ_CP033983 | Strain P2D1C1 plasmid unnamed1 | ABS | N/A | ABS | NCBI |
| 616 | NZ_CP033984 | Strain P2D1C1 plasmid unnamed2 | ABS | N/A | ABS | NCBI |
| 617 | NZ_CP033985 | Strain P2D1C1 plasmid unnamed3 | ABS | N/A | ABS | NCBI |
| 618 | NZ_CP033986 | Strain P2D1C1 plasmid unnamed4 | ABS | N/A | ABS | NCBI |
| 619 | NZ_CP033987 | Strain P2D1C1 | ABS | N/A | ABS | NCBI |
| 620 | NZ_CP033988 | Strain P1D14C1 plasmid unnamed1 | ABS | N/A | ABS | NCBI |
| 621 | NZ_CP033989 | Strain P1D14C1 plasmid unnamed2 | ABS | N/A | ABS | NCBI |
| N/A: No applique, ABS: Absent, NCBI: National Center for Biotechnology Information, ENA: European Nucleotide Archive, *Evidence level 4 and 3, *cas* genes | | | | | | |
| **Supplementary table A. Table of CRISPR-Cas system- and CRISPR-Cas system non-bearing strains of *Staphylococcus aureus* (continuation).** | | | | | | |
| **#** | **ACCESSION NUMBER** | **DESCRIPTION** | **CRISPR-Cas system** | **EVIDENCE LEVEL** | ***cas* GENES** | **DATABASE** |
| 622 | NZ_CP033990 | Strain P1D14C1 | ABS | N/A | ABS | NCBI |
| 623 | NZ_CP033994 | Strain P1D9C1 plasmid unnamed1 | ABS | N/A | ABS | NCBI |
| 624 | NZ_CP033995 | Strain P1D9C1 plasmid unnamed2 | ABS | N/A | ABS | NCBI |
| 625 | NZ_CP033996 | Strain P1D9C1 | ABS | N/A | ABS | NCBI |
| 626 | NZ_CP033997 | Strain P1D8C2 plasmid unnamed1 | ABS | N/A | ABS | NCBI |
| 627 | NZ_CP033998 | Strain P1D8C2 plasmid unnamed2 | ABS | N/A | ABS | NCBI |
| 628 | NZ_CP033999 | Strain P1D8C2 | ABS | N/A | ABS | NCBI |
| 629 | NZ_CP034000 | Strain P1D8C1 plasmid unnamed1 | ABS | N/A | ABS | NCBI |
| 630 | NZ_CP034001 | Strain P1D8C1 plasmid unnamed2 | ABS | N/A | ABS | NCBI |
| 631 | NZ_CP034002 | Strain P1D8C1 | ABS | N/A | ABS | NCBI |
| 632 | NZ_CP034003 | Strain P1D7C1 plasmid unnamed1 | ABS | N/A | ABS | NCBI |
| 633 | NZ_CP034004 | Strain P1D7C1 plasmid unnamed2 | ABS | N/A | ABS | NCBI |
| 634 | NZ_CP034005 | Strain P1D7C1 | ABS | N/A | ABS | NCBI |
| 635 | NZ_CP034006 | Strain P1D5C2 plasmid unnamed1 | ABS | N/A | ABS | NCBI |
| 636 | NZ_CP034007 | Strain P1D5C2 plasmid unnamed2 | ABS | N/A | ABS | NCBI |
| 637 | NZ_CP034008 | Strain P1D5C2 | ABS | N/A | ABS | NCBI |
| 638 | NZ_CP034009 | Strain P1D5C1 plasmid unnamed1 | ABS | N/A | ABS | NCBI |
| 639 | NZ_CP034010 | Strain P1D5C1 plasmid unnamed2 | ABS | N/A | ABS | NCBI |
| 640 | NZ_CP034011 | Strain P1D5C1 | ABS | N/A | ABS | NCBI |
| 641 | NZ_CP034012 | Strain P1D1C1 plasmid unnamed1 | ABS | N/A | ABS | NCBI |
| 642 | NZ_CP034013 | Strain P1D1C1 plasmid unnamed2 | ABS | N/A | ABS | NCBI |
| 643 | NZ_CP034014 | Strain P1D1C1 | ABS | N/A | ABS | NCBI |
| 644 | NZ_CP034098 | Strain 80wphwpl_v1 | ABS | N/A | ABS | NCBI |
| N/A: No applique, ABS: Absent, NCBI: National Center for Biotechnology Information, ENA: European Nucleotide Archive, *Evidence level 4 and 3, *cas* genes | | | | | | |
| **Supplementary table A. Table of CRISPR-Cas system- and CRISPR-Cas system non-bearing strains of *Staphylococcus aureus* (continuation).** | | | | | | |
| **#** | **ACCESSION NUMBER** | **DESCRIPTION** | **CRISPR-Cas system** | **EVIDENCE LEVEL** | ***cas* GENES** | **DATABASE** |
| 645 | NZ_CP034102 | Strain O267 | ABS | N/A | ABS | NCBI |
| 646 | NZ_CP034257 | Strain P1D12C1 plasmid unnamed2 | ABS | N/A | ABS | NCBI |
| 647 | NZ_CP034258 | Strain P1D12C1 plasmid unnamed1 | ABS | N/A | ABS | NCBI |
| 648 | NZ_CP034259 | Strain P1D12C1 | ABS | N/A | ABS | NCBI |
| 649 | NZ_CP034349 | Subsp. *aureus* Strain 80wphpl | ABS | N/A | ABS | NCBI |
| 650 | NZ_CP034441 | Strain PMB 81-4 | ABS | N/A | ABS | NCBI |
| 651 | NZ_CP034486 | Strain PMB 64-1 | ABS | N/A | ABS | NCBI |
| 652 | NZ_CP035003 | Strain PCFA-221 | ABS | N/A | ABS | NCBI |
| 653 | NZ_CP035004 | Strain PCFA-221 plasmid pP541 | ABS | N/A | ABS | NCBI |
| 654 | NZ_CP035005 | Strain PCFH-226 | ABS | N/A | ABS | NCBI |
| 655 | NZ_CP035006 | Strain PCFH-226 plasmid pP541 | ABS | N/A | ABS | NCBI |
| 656 | NZ_CP035101 | Subsp. *aureus* Strain ATCC 12600 | ABS | N/A | ABS | NCBI |
| 657 | NZ_CP035102 | Subsp. *aureus* Strain ATCC 12600 plasmid unnamed | ABS | N/A | ABS | NCBI |
| 658 | NZ_CP035369 | Strain LAC | ABS | N/A | ABS | NCBI |
| 659 | NZ_CP035370 | Strain LAC plasmid unnamed | ABS | N/A | ABS | NCBI |
| 660 | NZ_CP035670 | Subsp. *aureus* Strain VB9352 | ABS | N/A | ABS | NCBI |
| 661 | NZ_CP035671 | Subsp. *aureus* Strain VB31683 | ABS | N/A | ABS | NCBI |
| 662 | NZ_CP035791 | Strain 592 | ABS | N/A | ABS | NCBI |
| 663 | NZ_CP035792 | Strain 592 plasmid unnamed | ABS | N/A | ABS | NCBI |
| 664 | NZ_CP038021 | Strain 04-002 | ABS | N/A | ABS | NCBI |
| 665 | NZ_CP038268 | Strain 055 isolate B118 | ABS | N/A | ABS | NCBI |
| 666 | NZ_CP038269 | Strain O331 isolate B114 | ABS | N/A | ABS | NCBI |
| 667 | NZ_CP038270 | Strain O408 isolate B115 | ABS | N/A | ABS | NCBI |
| N/A: No applique, ABS: Absent, NCBI: National Center for Biotechnology Information, ENA: European Nucleotide Archive, *Evidence level 4 and 3, *cas* genes | | | | | | |
| **Supplementary table A. Table of CRISPR-Cas system- and CRISPR-Cas system non-bearing strains of *Staphylococcus aureus* (continuation).** | | | | | | |
| **#** | **ACCESSION NUMBER** | **DESCRIPTION** | **CRISPR-Cas system** | **EVIDENCE LEVEL** | ***cas* GENES** | **DATABASE** |
| 668 | NZ_CP038460 | Strain B119 | ABS | N/A | ABS | NCBI |
| 669 | NZ_CP038612 | Strain O268 | ABS | N/A | ABS | NCBI |
| 670 | NZ_CP038819 | Strain O82 | ABS | N/A | ABS | NCBI |
| 671 | NZ_CP038850 | Strain Lr2 | ABS | N/A | ABS | NCBI |
| 672 | NZ_CP038851 | Strain Lr2 plasmid pLr2-1 | ABS | N/A | ABS | NCBI |
| 673 | NZ_CP039156 | Subsp. *aureus* Strain WCUH29 | ABS | N/A | ABS | NCBI |
| 674 | NZ_CP039157 | Strain p10 | ABS | N/A | ABS | NCBI |
| 675 | NZ_CP039158 | Strain P10 plasmid pP10-1 | ABS | N/A | ABS | NCBI |
| 676 | NZ_CP039159 | Strain P10 plasmid pP10-2 | ABS | N/A | ABS | NCBI |
| 677 | NZ_CP039160 | Strain Lr6 | ABS | N/A | ABS | NCBI |
| 678 | NZ_CP039161 | Strain Lr6 plasmid pLr6 | ABS | N/A | ABS | NCBI |
| 679 | NZ_CP039162 | Strain Lr12 | ABS | N/A | ABS | NCBI |
| 680 | NZ_CP039163 | Strain Lr12 plasmid pLr12 | ABS | N/A | ABS | NCBI |
| 681 | NZ_CP039164 | Strain R46 | ABS | N/A | ABS | NCBI |
| 682 | NZ_CP039165 | Strain R46 plasmid pR46-1 | ABS | N/A | ABS | NCBI |
| 683 | NZ_CP039166 | Strain R46 plasmid pR46-2 | ABS | N/A | ABS | NCBI |
| 684 | NZ_CP039167 | Strain R50 | ABS | N/A | ABS | NCBI |
| 685 | NZ_CP039168 | Strain R50 plasmid pR50 | ABS | N/A | ABS | NCBI |
| 686 | NZ_CP039448 | Strain VGC1 | ABS | N/A | ABS | NCBI |
| 687 | NZ_CP039449 | Strain VGC1 plasmid pVGC1_1 | ABS | N/A | ABS | NCBI |
| 688 | NZ_CP039450 | Strain VGC1 plasmid pVGC1_2 | ABS | N/A | ABS | NCBI |
| 689 | NZ_CP039992 | Strain Lr3 | ABS | N/A | ABS | NCBI |
| 690 | NZ_CP039996 | Subsp. *aureus* M013 plasmid pM013 | ABS | N/A | ABS | NCBI |
| N/A: No applique, ABS: Absent, NCBI: National Center for Biotechnology Information, ENA: European Nucleotide Archive, *Evidence level 4 and 3, *cas* genes | | | | | | |
| **Supplementary table A. Table of CRISPR-Cas system- and CRISPR-Cas system non-bearing strains of *Staphylococcus aureus* (continuation).** | | | | | | |
| **#** | **ACCESSION NUMBER** | **DESCRIPTION** | **CRISPR-Cas system** | **EVIDENCE LEVEL** | ***cas* GENES** | **DATABASE** |
| 691 | NZ_CP040229 | Strain GD487 | ABS | N/A | ABS | NCBI |
| 692 | NZ_CP040230 | Strain GD1108 | ABS | N/A | ABS | NCBI |
| 693 | NZ_CP040232 | Strain GD1706 | ABS | N/A | ABS | NCBI |
| 694 | NZ_CP040233 | Strain GD1696 | ABS | N/A | ABS | NCBI |
| 695 | NZ_CP040560 | Strain Col52-A5 | ABS | N/A | ABS | NCBI |
| 696 | NZ_CP040561 | Strain Col52-A5 plasmid pCOL52-A5 | ABS | N/A | ABS | NCBI |
| 697 | NZ_CP040619 | Strain J01 | ABS | N/A | ABS | NCBI |
| 698 | NZ_CP040620 | Strain J01 plasmid pJ01-01 | ABS | N/A | ABS | NCBI |
| 699 | NZ_CP040621 | Strain J01 plasmid pJ01-02 | ABS | N/A | ABS | NCBI |
| 700 | NZ_CP040622 | Strain JKD6004 | ABS | N/A | ABS | NCBI |
| 701 | NZ_CP040623 | Strain D592-HR | ABS | N/A | ABS | NCBI |
| 702 | NZ_CP040624 | Strain D592-HR plasmid pSaD592-HR | ABS | N/A | ABS | NCBI |
| 703 | NZ_CP040625 | Strain JKD6004-DR | ABS | N/A | ABS | NCBI |
| 704 | NZ_CP040665 | Strain D592 | ABS | N/A | ABS | NCBI |
| 705 | NZ_CP040666 | Strain D592 plasmid pSaD592 | ABS | N/A | ABS | NCBI |
| 706 | NZ_CP040998 | Strain FDAARGOS_773 | ABS | N/A | ABS | NCBI |
| 707 | NZ_CP041000 | Strain FDAARGOS_773 plasmid unnamed2 | ABS | N/A | ABS | NCBI |
| 708 | NZ_CP041009 | Strain FDAARGOS_776 plasmid unnamed1 | ABS | N/A | ABS | NCBI |
| 709 | NZ_CP041010 | Strain FDAARGOS_766 | ABS | N/A | ABS | NCBI |
| 710 | NZ_CP041037 | Strain NP66 | ABS | N/A | ABS | NCBI |
| 711 | NZ_CP042003 | Strain B3-14B | ABS | N/A | ABS | NCBI |
| 712 | NZ_CP042004 | Strain B3-14B plasmid pSALNT46 | ABS | N/A | ABS | NCBI |
| 713 | NZ_CP042005 | Strain B3-14B plasmid pSALNT16 | ABS | N/A | ABS | NCBI |
| N/A: No applique, ABS: Absent, NCBI: National Center for Biotechnology Information, ENA: European Nucleotide Archive, *Evidence level 4 and 3, *cas* genes | | | | | | |
| **Supplementary table A. Table of CRISPR-Cas system- and CRISPR-Cas system non-bearing strains of *Staphylococcus aureus* (continuation).** | | | | | | |
| **#** | **ACCESSION NUMBER** | **DESCRIPTION** | **CRISPR-Cas system** | **EVIDENCE LEVEL** | ***cas* GENES** | **DATABASE** |
| 714 | NZ_CP042006 | Strain B3-14B plasmid pSALNT4.9 | ABS | N/A | ABS | NCBI |
| 715 | NZ_CP042007 | Strain B4-14B plasmid pSALNT2.2 | ABS | N/A | ABS | NCBI |
| 716 | NZ_CP042043 | Strain B2-15A | ABS | N/A | ABS | NCBI |
| 717 | NZ_CP042044 | Strain B2-15A plasmid pSALNCL1.4 | ABS | N/A | ABS | NCBI |
| 718 | NZ_CP042045 | Strain B2-15A plasmid pSALNCL17 | ABS | N/A | ABS | NCBI |
| 719 | NZ_CP042046 | Strain B2-7A | ABS | N/A | ABS | NCBI |
| 720 | NZ_CP042047 | Strain B2-7A plasmid pSALNBL75 | ABS | N/A | ABS | NCBI |
| 721 | NZ_CP042107 | Strain B8-13D | ABS | N/A | ABS | NCBI |
| 722 | NZ_CP042108 | Strain B8-13D plasmid pSALNCG17 | ABS | N/A | ABS | NCBI |
| 723 | NZ_CP042109 | Strain B8-13D plasmid pSALNCG1.5 | ABS | N/A | ABS | NCBI |
| 724 | NZ_CP042153 | Strain B4-59C | ABS | N/A | ABS | NCBI |
| 725 | NZ_CP042154 | Strain B4-59C plasmid pSALNC14 | ABS | N/A | ABS | NCBI |
| 726 | NZ_CP042155 | Strain B4-59C plasmid pSALNC2.8 | ABS | N/A | ABS | NCBI |
| 727 | NZ_CP042156 | Strain B4-59C plasmid pSALNC1.4-2 | ABS | N/A | ABS | NCBI |
| 728 | NZ_CP042157 | Strain B3-17D | ABS | N/A | ABS | NCBI |
| 729 | NZ_CP042158 | Strain B3-17D plasmid pSALNC17 | ABS | N/A | ABS | NCBI |
| 730 | NZ_CP042159 | Strain B3-17D plasmid pSALNC1.6 | ABS | N/A | ABS | NCBI |
| 731 | NZ_CP042160 | Strain B3-17D plasmid pSALNC1.4-1 | ABS | N/A | ABS | NCBI |
| 732 | NZ_CP042346 | Strain BSN9S | ABS | N/A | ABS | NCBI |
| 733 | NZ_CP042347 | Strain BSN9S plasmid pSaBSN9 | ABS | N/A | ABS | NCBI |
| 734 | NZ_CP042348 | Strain BSN9R | ABS | N/A | ABS | NCBI |
| 735 | NZ_CP042349 | Strain BSN9R plasmid pSaBSN9S | ABS | N/A | ABS | NCBI |
| 736 | NZ_CP042650 | Strain X22 | ABS | N/A | ABS | NCBI |
| N/A: No applique, ABS: Absent, NCBI: National Center for Biotechnology Information, ENA: European Nucleotide Archive, *Evidence level 4 and 3, *cas* genes | | | | | | |
| **Supplementary table A. Table of CRISPR-Cas system- and CRISPR-Cas system non-bearing strains of *Staphylococcus aureus* (continuation).** | | | | | | |
| **#** | **ACCESSION NUMBER** | **DESCRIPTION** | **CRISPR-Cas system** | **EVIDENCE LEVEL** | ***cas* GENES** | **DATABASE** |
| 737 | NZ_CP042651 | Strain X22 plasmid unnamed | ABS | N/A | ABS | NCBI |
| 738 | NZ_CP043302 | Strain 16445 | ABS | N/A | ABS | NCBI |
| 739 | NZ_CP043303 | Strain 16445 plasmid unnamed1 | ABS | N/A | ABS | NCBI |
| 740 | NZ_CP043386 | Strain NRS384 | ABS | N/A | ABS | NCBI |
| 741 | NZ_CP043387 | Strain NRS384 plasmid pNRS384_1 | ABS | N/A | ABS | NCBI |
| 742 | NZ_CP043388 | Strain NS384 plasmid pNRS384_2 | ABS | N/A | ABS | NCBI |
| 743 | NZ_CP043389 | Strain NRS384-rpoB-H481N-SCV | ABS | N/A | ABS | NCBI |
| 744 | NZ_CP043390 | Strain NRSA384-rpoB-H481N-SCV plasmid pNRS384_1 | ABS | N/A | ABS | NCBI |
| 745 | NZ_CP043391 | Strain NRSA384-rpoB-H481N-SCV plasmid pNRS384_2 | ABS | N/A | ABS | NCBI |
| 746 | NZ_CP043392 | Strain NRS384-rpoB-H481N-NCV | ABS | N/A | ABS | NCBI |
| 747 | NZ_CP043393 | Strain NRS384-rpoB-H481N-NCV plasmid pNRS384_1 | ABS | N/A | ABS | NCBI |
| 748 | NZ_CP043394 | Strain NRS384-rpoB-H481-NCV plasmid pNRS384_2 | ABS | N/A | ABS | NCBI |
| 749 | NZ_CP044105 | Strain FDAARGOS_660 plasmid unnamed1 | ABS | N/A | ABS | NCBI |
| 750 | NZ_CP044106 | Strain FDAARGOS_660 | ABS | N/A | ABS | NCBI |
| 751 | NZ_CP045472 | Strain ZY05 | ABS | N/A | ABS | NCBI |
| 752 | NZ_CP045473 | Strain ZY05 plasmid pZY05 | ABS | N/A | ABS | NCBI |
| 753 | NZ_CP045866 | Strain CFSAN007894 | ABS | N/A | ABS | NCBI |
| 754 | NZ_CP045867 | Strain CFSAN007894 plasmid pCFSAN00794 | ABS | N/A | ABS | NCBI |
| 755 | NZ_CP0144431 | Strain USA300-SUR19 plasmid pUSA04-1-1sur19 | ABS | N/A | ABS | NCBI |
| 756 | NZ_CP0409991 | Strain FDAARGOS_773 plasmid unnamed1 | ABS | N/A | ABS | NCBI |
| 757 | NZ_LT992477 | Strain Isolate 22_LA_562 | ABS | N/A | ABS | NCBI |
| 758 | NZ_LN626917 | Strain ILRI Eymole1_1 | ABS | N/A | ABS | NCBI |
| 759 | NZ_LN831036 | Strain NCTC13435 | ABS | N/A | ABS | NCBI |
| N/A: No applique, ABS: Absent, NCBI: National Center for Biotechnology Information, ENA: European Nucleotide Archive, *Evidence level 4 and 3, *cas* genes | | | | | | |
| **Supplementary table A. Table of CRISPR-Cas system- and CRISPR-Cas system non-bearing strains of *Staphylococcus aureus* (continuation).** | | | | | | |
| **#** | **ACCESSION NUMBER** | **DESCRIPTION** | **CRISPR-Cas system** | **EVIDENCE LEVEL** | ***cas* GENES** | **DATABASE** |
| 760 | NZ_LN831037 | Strain NCTC13435 plasmid2 | ABS | N/A | ABS | NCBI |
| 761 | NZ_LN831038 | Strain NCTC13435 plasmid 3 | ABS | N/A | ABS | NCBI |
| 762 | NZ_LN854556 | Strain BB155 | ABS | N/A | ABS | NCBI |
| 763 | NZ_LR027869 | Strain BPH2869 | ABS | N/A | ABS | NCBI |
| 764 | NZ_LR027870 | Strain BPH2019 | ABS | N/A | ABS | NCBI |
| 765 | NZ_LR027871 | Strain BPH2019 plasmid 2 | ABS | N/A | ABS | NCBI |
| 766 | NZ_LR027872 | Strain BPH2019 plasmid 3 | ABS | N/A | ABS | NCBI |
| 767 | NZ_LR027873 | Strain BPH2070 | ABS | N/A | ABS | NCBI |
| 768 | NZ_LR027874 | Strain BPH2056 | ABS | N/A | ABS | NCBI |
| 769 | NZ_LR027875 | Strain BPH2056 plasmid 2 | ABS | N/A | ABS | NCBI |
| 770 | NZ_LR027876 | Strain JKD6009 | ABS | N/A | ABS | NCBI |
| 771 | NZ_LR027877 | Strain BPH3244 | ABS | N/A | ABS | NCBI |
| 772 | NZ_LR027878 | Strain BPH2003 | ABS | N/A | ABS | NCBI |
| 773 | NZ_LR027879 | Strain BPH2003 plasmid2 | ABS | N/A | ABS | NCBI |
| 774 | NZ_LR134193 | Strain NCTC13616 | ABS | N/A | ABS | NCBI |
| 775 | NZ_LR130509 | Strain BPH2760 | ABS | N/A | ABS | NCBI |
| 776 | NZ_LR130510 | Strain BPH2760 plasmid 2 | ABS | N/A | ABS | NCBI |
| 777 | NZ_LR130511 | Strain BPH2819 | ABS | N/A | ABS | NCBI |
| 778 | NZ_LR130512 | Strain BPH2819 plasmid 2 | ABS | N/A | ABS | NCBI |
| 779 | NZ_LR130513 | Strain BPH2900 | ABS | N/A | ABS | NCBI |
| 780 | NZ_LR130514 | Strain BPH2900 plasmid 2 | ABS | N/A | ABS | NCBI |
| 781 | NZ_LR130515 | Strain BPH2947 | ABS | N/A | ABS | NCBI |
| 782 | NZ_LR130516 | Strain BPH2947 plasmid 2 | ABS | N/A | ABS | NCBI |
| N/A: No applique, ABS: Absent, NCBI: National Center for Biotechnology Information, ENA: European Nucleotide Archive, *Evidence level 4 and 3, *cas* genes | | | | | | |
| **Supplementary table A. Table of CRISPR-Cas system- and CRISPR-Cas system non-bearing strains of *Staphylococcus aureus* (continuation).** | | | | | | |
| **#** | **ACCESSION NUMBER** | **DESCRIPTION** | **CRISPR-Cas system** | **EVIDENCE LEVEL** | ***cas* GENES** | **DATABASE** |
| 783 | NZ_LR130517 | Strain BPH2947 plasmid 3 | ABS | N/A | ABS | NCBI |
| 784 | NZ_LR130518 | Strain BPH2986 | ABS | N/A | ABS | NCBI |
| 785 | NZ_LR130519 | Strain BPH2986 plasmid 2 | ABS | N/A | ABS | NCBI |
| 786 | NZ_LR130520 | Strain BPH2986 plasmid 3 | ABS | N/A | ABS | NCBI |
| 787 | NZ_LR133917 | Strain NCTC8317 | ABS | N/A | ABS | NCBI |
| 788 | NZ_LR134084 | Strain NCTC13552 | ABS | N/A | ABS | NCBI |
| 789 | NZ_LR134085 | Strain NCTC12233 | ABS | N/A | ABS | NCBI |
| 790 | NZ_LR134086 | Strain NCTC13142 | ABS | N/A | ABS | NCBI |
| 791 | NZ_LR134087 | Strain NCTC7121 | ABS | N/A | ABS | NCBI |
| 792 | NZ_LR134090 | Strain NCTC9555 | ABS | N/A | ABS | NCBI |
| 793 | NZ_LR134091 | Strain NCTC4137 | ABS | N/A | ABS | NCBI |
| 794 | NZ_LR134093 | Strain NCTC11965 | ABS | N/A | ABS | NCBI |
| 795 | NZ_LR134139 | Strain NCTC4163 | ABS | N/A | ABS | NCBI |
| 796 | NZ_LR134271 | Strain NCTC988 | ABS | N/A | ABS | NCBI |
| 797 | NZ_LR134305 | Strain NCTC1803 | ABS | N/A | ABS | NCBI |
| 798 | NZ_LR134351 | Strain NCTC13811 | ABS | N/A | ABS | NCBI |
| 799 | NZ_LS483300 | Strain NCTC7485 | ABS | N/A | ABS | NCBI |
| 800 | NZ_LS483301 | Strain NCTC13394 | ABS | N/A | ABS | NCBI |
| 801 | NZ_LS483302 | Strain NCTC8726 | ABS | N/A | ABS | NCBI |
| 802 | NZ_LS483308 | Strain NCTC13137 | ABS | N/A | ABS | NCBI |
| 803 | NZ_LS483309 | Strain NCTC9944 | ABS | N/A | ABS | NCBI |
| 804 | NZ_LS483310 | Strain NCTC9752 | ABS | N/A | ABS | NCBI |
| 805 | NZ_LS483311 | Strain NCTC6136 | ABS | N/A | ABS | NCBI |
| N/A: No applique, ABS: Absent, NCBI: National Center for Biotechnology Information, ENA: European Nucleotide Archive, *Evidence level 4 and 3, *cas* genes | | | | | | |
| **Supplementary table A. Table of CRISPR-Cas system- and CRISPR-Cas system non-bearing strains of *Staphylococcus aureus* (continuation).** | | | | | | |
| **#** | **ACCESSION NUMBER** | **DESCRIPTION** | **CRISPR-Cas system** | **EVIDENCE LEVEL** | ***cas* GENES** | **DATABASE** |
| 806 | NZ_LS483314 | Strain NCTC3761 | ABS | N/A | ABS | NCBI |
| 807 | NZ_LS483316 | Strain NCTC13395 | ABS | N/A | ABS | NCBI |
| 808 | NZ_LS483317 | Strain NCTC5663 | ABS | N/A | ABS | NCBI |
| 809 | NZ_LS483319 | Strain NCTC13140 | ABS | N/A | ABS | NCBI |
| 810 | NZ_LS483350 | Strain NCTC11940 | ABS | N/A | ABS | NCBI |
| 811 | NZ_LS483365 | Strain NCTC8325 | ABS | N/A | ABS | NCBI |
| 812 | NZ_LS483484 | Strain NCTC13277 | ABS | N/A | ABS | NCBI |
| 813 | NZ_LT009690 | Strain NZAK3 | ABS | N/A | ABS | NCBI |
| 814 | NZ_LT009691 | Strain NZAK3 plasmid 2 | ABS | N/A | ABS | NCBI |
| 815 | NZ_LT598688 | Strain Sa_Newman_UoM | ABS | N/A | ABS | NCBI |
| 816 | NZ_LT615218 | Strain AUS0325 | ABS | N/A | ABS | NCBI |
| 817 | NZ_LT671859 | Subs. *aureus* Clinical isolate | ABS | N/A | ABS | NCBI |
| 818 | NZ_LT671860 | Subs. *aureus* Clinical isolate plasmid II | ABS | N/A | ABS | NCBI |
| 819 | NZ_LT699704 | Strain NZ15MR0322 | ABS | N/A | ABS | NCBI |
| 820 | NZ_LT699705 | Strain NZ15MR0322 plasmid 2 | ABS | N/A | ABS | NCBI |
| 821 | NZ_LT992434 | Isolate 1549-WT | ABS | N/A | ABS | NCBI |
| 822 | NZ_LT992435 | Isolate 1549-SCV | ABS | N/A | ABS | NCBI |
| 823 | NZ_LT992436 | Isolate 1549-REV | ABS | N/A | ABS | NCBI |
| 824 | NZ_LT992456 | Isolate 1_1439 | ABS | N/A | ABS | NCBI |
| 825 | NZ_LT992457 | Isolate 1_1439 plasmid II | ABS | N/A | ABS | NCBI |
| 826 | NZ_LT992458 | Isolate 7_4623 | ABS | N/A | ABS | NCBI |
| 827 | NZ_LT992460 | Isolate 9_LA_281 | ABS | N/A | ABS | NCBI |
| 828 | NZ_LT992461 | Isolate 8_LA_272 | ABS | N/A | ABS | NCBI |
| N/A: No applique, ABS: Absent, NCBI: National Center for Biotechnology Information, ENA: European Nucleotide Archive, *Evidence level 4 and 3, *cas* genes | | | | | | |
| **Supplementary table A. Table of CRISPR-Cas system- and CRISPR-Cas system non-bearing strains of *Staphylococcus aureus* (continuation).** | | | | | | |
| **#** | **ACCESSION NUMBER** | **DESCRIPTION** | **CRISPR-Cas system** | **EVIDENCE LEVEL** | ***cas* GENES** | **DATABASE** |
| 829 | NZ_LT992462 | Isolate 5_3949 | ABS | N/A | ABS | NCBI |
| 830 | NZ_LT992463 | Isolate 2_LA_86 | ABS | N/A | ABS | NCBI |
| 831 | NZ_LT992464 | Isolate 3_LA_115 | ABS | N/A | ABS | NCBI |
| 832 | NZ_LT992465 | Isolate 6 _LA_232 | ABS | N/A | ABS | NCBI |
| 833 | NZ_LT992466 | Isolate 4_LA_208 | ABS | N/A | ABS | NCBI |
| 834 | NZ_LT992467 | Isolate 16_LA_309 | ABS | N/A | ABS | NCBI |
| 835 | NZ_LT992468 | Isolate 12_LA_293 | ABS | N/A | ABS | NCBI |
| 836 | NZ_LT992469 | Isolate 15_LA_305 | ABS | N/A | ABS | NCBI |
| 837 | NZ_LT992470 | Isolate 13_LA_301 | ABS | N/A | ABS | NCBI |
| 838 | NZ_LT992471 | Isolate 17_LA_343 | ABS | N/A | ABS | NCBI |
| 839 | NZ_LT992472 | Isolate 10_5235 | ABS | N/A | ABS | NCBI |
| 840 | NZ_LT992473 | Isolate 14_5418 | ABS | N/A | ABS | NCBI |
| 841 | NZ_LT992474 | Isolate 19_LA_388 | ABS | N/A | ABS | NCBI |
| 842 | NZ_LT992475 | Isolate 20_LA_415 | ABS | N/A | ABS | NCBI |
| 843 | NZ_LT992476 | Isolate 21_LA_436 | ABS | N/A | ABS | NCBI |
| 844 | NZ_LT996889 | Isolate 24117-REV | ABS | N/A | ABS | NCBI |
| 845 | NZ_LT996890 | Isolate 24117-SCV | ABS | N/A | ABS | NCBI |
| 846 | NZ_LT996891 | Isolate 24117-WT | ABS | N/A | ABS | NCBI |
| 847 | AB255366 | Strain IMCJ1379 plasmid pSA1379 | ABS | N/A | ABS | ENA |
| 848 | AB860416 | Strain Tokyo11212 pathogenicity island SaPITokyo11212 | ABS | N/A | ABS | ENA |
| 849 | AP015012 | Strain No. 10 | ABS | N/A | ABS | ENA |
| 850 | AP015013 | Strain No. 10 plasmid pno10 | ABS | N/A | ABS | ENA |
| 851 | AP019713 | Strain Tokyo12482 | ABS | N/A | ABS | ENA |
| N/A: No applique, ABS: Absent, NCBI: National Center for Biotechnology Information, ENA: European Nucleotide Archive, *Evidence level 4 and 3, *cas* genes | | | | | | |
| **Supplementary table A. Table of CRISPR-Cas system- and CRISPR-Cas system non-bearing strains of *Staphylococcus aureus* (continuation).** | | | | | | |
| **#** | **ACCESSION NUMBER** | **DESCRIPTION** | **CRISPR-Cas system** | **EVIDENCE LEVEL** | ***cas* GENES** | **DATABASE** |
| 852 | CM002749 | Strain 880 plasmid pHMPREF1625_1 | ABS | N/A | ABS | ENA |
| 853 | CM002750 | Strain 880 plasmid pHMPREF1625_2 | ABS | N/A | ABS | ENA |
| 854 | CM003164 | Subsp. *aureus* Strain A900624 plasmid pA900624 | ABS | N/A | ABS | ENA |
| 855 | CM003165 | Subsp. *aureus* Strain CCM5757 plasmid pCCM5757-1 | ABS | N/A | ABS | ENA |
| 856 | CM003166 | Subsp. *aureus* Strain CCM5757 plasmid pCCM5757-2 | ABS | N/A | ABS | ENA |
| 857 | CM003167 | Subsp. *aureus* Strain DSM 799 plasmid pDSM799 | ABS | N/A | ABS | ENA |
| 858 | CM003168 | Subsp. *aureus* Strain FRI1151m plasmid pFRI1151m | ABS | N/A | ABS | ENA |
| 859 | CM003169 | Subsp. *aureus* Strain SA-022 plasmid pSA-022 | ABS | N/A | ABS | ENA |
| 860 | CM003170 | Subsp. *aureus* Strain SA-038 plasmid pSA-038 | ABS | N/A | ABS | ENA |
| 861 | CM003171 | Subsp. *aureus* Strain SA-260 plasmid pSA-260 | ABS | N/A | ABS | ENA |
| 862 | CM003172 | Subsp. *aureus* Strain SA-047 plasmid pSA-047 | ABS | N/A | ABS | ENA |
| 863 | CM003173 | Subsp. *aureus* Strain SA-067 plasmid pSA-067 | ABS | N/A | ABS | ENA |
| 864 | CM003311 | Subsp. *aureus* Strain B6 plasmid pSA-B6-1 | ABS | N/A | ABS | ENA |
| 865 | CM003519 | Strain CC017 plasmid pSA-CC017-1 | ABS | N/A | ABS | ENA |
| 866 | CM003520 | Strain CC022 plasmid pSA-CC022-1 | ABS | N/A | ABS | ENA |
| 867 | CM003521 | Strain CC022 plasmid pSA-CC022-2 | ABS | N/A | ABS | ENA |
| 868 | CM003522 | Strain CC169 plasmid pSA-CC169-1 | ABS | N/A | ABS | ENA |
| 869 | CM003523 | Strain CC169 plasmid pSA-CC169-2 | ABS | N/A | ABS | ENA |
| 870 | CM003524 | Strain CC072 plasmid pSA-CC072-1 | ABS | N/A | ABS | ENA |
| 871 | CM003525 | Strain CC175 plasmid pSA-CC175-1 | ABS | N/A | ABS | ENA |
| 872 | CM003527 | Strain CC445 plasmid pSA-CC445-1 | ABS | N/A | ABS | ENA |
| 873 | CM003616 | Subsp. *aureus* Strain MRSA_S1 plasmid pHUGS1 | ABS | N/A | ABS | ENA |
| 874 | CM003617 | Subsp. *aureus* Strain MRSA_S5 plasmid pSAHUG_S5a | ABS | N/A | ABS | ENA |
| N/A: No applique, ABS: Absent, NCBI: National Center for Biotechnology Information, ENA: European Nucleotide Archive, *Evidence level 4 and 3, *cas* genes | | | | | | |
| **Supplementary table A. Table of CRISPR-Cas system- and CRISPR-Cas system non-bearing strains of *Staphylococcus aureus* (continuation).** | | | | | | |
| **#** | **ACCESSION NUMBER** | **DESCRIPTION** | **CRISPR-Cas system** | **EVIDENCE LEVEL** | ***cas* GENES** | **DATABASE** |
| 875 | CM003618 | Subsp. *aureus* Strain MRSA_S5 plasmid pSAHUG_S5b | ABS | N/A | ABS | ENA |
| 876 | CM003619 | Subsp. *aureus* Strain MRSA_S2 plasmid pHUGS2 | ABS | N/A | ABS | ENA |
| 877 | CM003620 | Subsp. *aureus* Strain MRSA_S15 plasmid pSAHUG_S15a | ABS | N/A | ABS | ENA |
| 878 | CM003621 | Subsp. *aureus* Strain MRSA_S15 plasmid pSAHUG_S15b | ABS | N/A | ABS | ENA |
| 879 | CM003622 | Subsp. *aureus* Strain MRSA_S20 plasmid pSAHUG_S20a | ABS | N/A | ABS | ENA |
| 880 | CM003623 | Subsp. *aureus* Strain MRSA_S19 plasmid pSAHUG_S19a | ABS | N/A | ABS | ENA |
| 881 | CM003624 | Subsp. *aureus* Strain MRSA_S19 plasmid pSAHUG_S19b | ABS | N/A | ABS | ENA |
| 882 | CM003625 | Subsp. *aureus* Strain MRSA_S26 plasmid pSAHUG_S26a | ABS | N/A | ABS | ENA |
| 883 | CM004484 | Subsp. *aureus* Strain 137dia93A plasmid pSA93A | ABS | N/A | ABS | ENA |
| 884 | CM007848 | Strain ATCC 27217 plasmid pATCC27217-2 | ABS | N/A | ABS | ENA |
| 885 | CM007849 | Strain ATCC 14458 plasmid pATCC14458-1 | ABS | N/A | ABS | ENA |
| 886 | CM007850 | Strain ATCC 14458 plasmid pATCC14458-2 | ABS | N/A | ABS | ENA |
| 887 | CM007851 | Strain ATCC 14458 plasmid pATCC14458-3 | ABS | N/A | ABS | ENA |
| 888 | CM007996 | Strain SCPM-O-B-7905 plasmid pMW2 | ABS | N/A | ABS | ENA |
| 889 | CM007997 | Strain SCPM-O-B-7906 plasmid pMW2 | ABS | N/A | ABS | ENA |
| 890 | CM009343 | Subsp. *aureus* Strain 1626.C01 plasmid p1626.C01a | ABS | N/A | ABS | ENA |
| 891 | CM009344 | Subsp. *aureus* Strain 1626.C01 plasmid p1626.C01b | ABS | N/A | ABS | ENA |
| 892 | CM009345 | Subsp. *aureus* Strain 1626.C01 plasmid p1626.C01c | ABS | N/A | ABS | ENA |
| 893 | CM010230 | Strain SAUR1404 plasmid pSAUR1404_A | ABS | N/A | ABS | ENA |
| 894 | CM010231 | Strain SAUR1404 plasmid pSAUR1404_B | ABS | N/A | ABS | ENA |
| 895 | CM010232 | Strain SAUR390 plasmid pSAUR390_A | ABS | N/A | ABS | ENA |
| 896 | CM010233 | Strain SAUR678 plasmid pSAUR678_A | ABS | N/A | ABS | ENA |
| 897 | CM010234 | Strain SAUR678 plasmid pSAUR678_B | ABS | N/A | ABS | ENA |
| N/A: No applique, ABS: Absent, NCBI: National Center for Biotechnology Information, ENA: European Nucleotide Archive, *Evidence level 4 and 3, *cas* genes | | | | | | |
| **Supplementary table A. Table of CRISPR-Cas system- and CRISPR-Cas system non-bearing strains of *Staphylococcus aureus* (continuation).** | | | | | | |
| **#** | **ACCESSION NUMBER** | **DESCRIPTION** | **CRISPR-Cas system** | **EVIDENCE LEVEL** | ***cas* GENES** | **DATABASE** |
| 898 | CM010667 | Strain AFIPCBER_B_8.4 plasmid pAFIPCBER_B84 | ABS | N/A | ABS | ENA |
| 899 | CM011642 | Strain SABHZ053 plasmid pSA053-1 | ABS | N/A | ABS | ENA |
| 900 | CM011643 | Strain SABHZ053 plasmid pSA053-2 | ABS | N/A | ABS | ENA |
| 901 | CM011644 | Strain SABHZ079 plasmid pSABHZ079-1 | ABS | N/A | ABS | ENA |
| 902 | CM011645 | Strain SABHZ079 plasmid pSABHZ079-2 | ABS | N/A | ABS | ENA |
| 903 | CM011646 | Strain SABHZ079 plasmid pSABHZ079-3 | ABS | N/A | ABS | ENA |
| 904 | CM011647 | Strain GD-G33 plasmid pG38 | ABS | N/A | ABS | ENA |
| 905 | CM011648 | Strain GD-G38 plasmid pG38 | ABS | N/A | ABS | ENA |
| 906 | CM012181 | Strain UGA22 plasmid pUGA22 | ABS | N/A | ABS | ENA |
| 907 | CM014774 | Strain SCPM-O-B-8680 plasmid unnamed | ABS | N/A | ABS | ENA |
| 908 | CM014775 | Strain SCPM-O-B-8681 plasmid unnamed | ABS | N/A | ABS | ENA |
| 909 | CM014776 | Strain SCPM-O-B-8479 plasmid unnamed | ABS | N/A | ABS | ENA |
| 910 | CM014777 | Strain SCPM-O-B-8478 plasmid unnamed | ABS | N/A | ABS | ENA |
| 911 | CM014778 | Strain SCPM-O-B-8480 plasmid unnamed | ABS | N/A | ABS | ENA |
| 912 | CM014779 | Strain SCPM-O-B-8472 plasmid unnamed | ABS | N/A | ABS | ENA |
| 913 | CM014780 | Strain SCPM-O-B-8477 plasmid unnamed | ABS | N/A | ABS | ENA |
| 914 | CM014781 | Strain SCPM-O-B-8473 plasmid unnamed | ABS | N/A | ABS | ENA |
| 915 | CM014782 | Strain SCPM-O-B-8474 plasmid unnamed | ABS | N/A | ABS | ENA |
| 916 | CM014783 | Strain SCPM-O-B-7904 plasmid unnamed | ABS | N/A | ABS | ENA |
| 917 | CM014784 | Strain SCPM-O-B-7907 plasmid unnamed | ABS | N/A | ABS | ENA |
| 918 | CM016517 | Strain MRSA SAZ_10 plasmid pSAZ10A | ABS | N/A | ABS | ENA |
| 919 | CM016518 | Strain MRSA SAZ_10 plasmid pSAZ10B | ABS | N/A | ABS | ENA |
| 920 | CM016519 | Strain MRSA SAZ_10 plasmid pSAZ1C | ABS | N/A | ABS | ENA |
| N/A: No applique, ABS: Absent, NCBI: National Center for Biotechnology Information, ENA: European Nucleotide Archive, *Evidence level 4 and 3, *cas* genes | | | | | | |
| **Supplementary table A. Table of CRISPR-Cas system- and CRISPR-Cas system non-bearing strains of *Staphylococcus aureus* (continuation).** | | | | | | |
| **#** | **ACCESSION NUMBER** | **DESCRIPTION** | **CRISPR-Cas system** | **EVIDENCE LEVEL** | ***cas* GENES** | **DATABASE** |
| 921 | CM017113 | Strain SAZ_31 plasmid pSAZ_31 | ABS | N/A | ABS | ENA |
| 922 | CM018788 | Strain ER04615.3 plasmid unnamed1 | ABS | N/A | ABS | ENA |
| 923 | CP002132 | Strain 18805 plasmid p18805-P03 | ABS | N/A | ABS | ENA |
| 924 | CP002133 | Strain 18805 plasmid p18805-P01 | ABS | N/A | ABS | ENA |
| 925 | CP002134 | Strain 18806 plasmid p18806-P03 | ABS | N/A | ABS | ENA |
| 926 | CP002135 | Strain 18807 plasmid p18807-P03 | ABS | N/A | ABS | ENA |
| 927 | CP002136 | Strain 18807 plasmid p18807-P01 | ABS | N/A | ABS | ENA |
| 928 | CP002137 | Strain 18808 plasmid p18808-P03 | ABS | N/A | ABS | ENA |
| 929 | CP002138 | Strain 18808 plasmid p18808-P01 | ABS | N/A | ABS | ENA |
| 930 | CP002139 | Strain 18809 plasmid p18809-P03 | ABS | N/A | ABS | ENA |
| 931 | CP002140 | Strain 18809 plasmid p18809-P01 | ABS | N/A | ABS | ENA |
| 932 | CP002141 | Strain 18810 plasmid p18810-P03 | ABS | N/A | ABS | ENA |
| 933 | CP002142 | Strain 18810 plasmid p18810-P01 | ABS | N/A | ABS | ENA |
| 934 | CP002143 | Strain 18811 plasmid p18811-P03 | ABS | N/A | ABS | ENA |
| 935 | CP002144 | Strain 18811 plasmid p18811-P01 | ABS | N/A | ABS | ENA |
| 936 | CP002145 | Strain 18813 plasmid p18813-P03 | ABS | N/A | ABS | ENA |
| 937 | CP002146 | Strain 18813 plasmid p18809-P04 | ABS | N/A | ABS | ENA |
| 938 | CP002147 | Strain 19321 plasmid p19321-P03 | ABS | N/A | ABS | ENA |
| 939 | CP002148 | Strain 19321 plasmid p19321-P01 | ABS | N/A | ABS | ENA |
| 940 | CP002149 | Strain LAC plasmid pLAC-P03 | ABS | N/A | ABS | ENA |
| 941 | CP002150 | Strain LAC plasmid pLAC-P01 | ABS | N/A | ABS | ENA |
| 942 | CP005288 | Strain Bmb9393 | ABS | N/A | ABS | ENA |
| 943 | CP005289 | Strain Bmb9393 plasmid pBmb9393 | ABS | N/A | ABS | ENA |
| N/A: No applique, ABS: Absent, NCBI: National Center for Biotechnology Information, ENA: European Nucleotide Archive, *Evidence level 4 and 3, *cas* genes | | | | | | |
| **Supplementary table A. Table of CRISPR-Cas system- and CRISPR-Cas system non-bearing strains of *Staphylococcus aureus* (continuation).** | | | | | | |
| **#** | **ACCESSION NUMBER** | **DESCRIPTION** | **CRISPR-Cas system** | **EVIDENCE LEVEL** | ***cas* GENES** | **DATABASE** |
| 944 | CP013959 | Strain V605 | ABS | N/A | ABS | ENA |
| 945 | CP023390 | Subsp. *aureus* Strain Newman Strain NYU_Newman | ABS | N/A | ABS | ENA |
| 946 | CP023391 | Subsp. *aureus* Strain Newman_D2C | ABS | N/A | ABS | ENA |
| 947 | CP024649 | Strain O11 | ABS | N/A | ABS | ENA |
| 948 | CP026074 | Strain HPV107 | ABS | N/A | ABS | ENA |
| 949 | CP027101 | Strain FDAARGOS_140 | ABS | N/A | ABS | ENA |
| 950 | CP027486 | Strain ST2594 isolate 004 | ABS | N/A | ABS | ENA |
| 951 | CP029199 | Strain *aureus* plasmid pFORC_090.1 | ABS | N/A | ABS | ENA |
| 952 | CP030375 | Strain ER04041.3 plasmid unnamed1 | ABS | N/A | ABS | ENA |
| 953 | CP030376 | Strain ER04041.3 plasmid unnamed2 | ABS | N/A | ABS | ENA |
| 954 | CP030377 | Strain ER04041.3 | ABS | N/A | ABS | ENA |
| 955 | CP030381 | Strain ER03761.3 | ABS | N/A | ABS | ENA |
| 956 | CP030382 | Strain ER03761.3 plasmid unnamed1 | ABS | N/A | ABS | ENA |
| 957 | CP030383 | Strain ER03761.3 plasmid unnamed2 | ABS | N/A | ABS | ENA |
| 958 | CP030384 | Strain ER00959.3 | ABS | N/A | ABS | ENA |
| 959 | CP030385 | Strain ER00959.3 plasmid unnamed1 | ABS | N/A | ABS | ENA |
| 960 | CP030386 | Strain ER01062.3 | ABS | N/A | ABS | ENA |
| 961 | CP030387 | Strain ER01062.3 plasmid unnamed1 | ABS | N/A | ABS | ENA |
| 962 | CP030388 | Strain ER01062.3 plasmid unnamed2 | ABS | N/A | ABS | ENA |
| 963 | CP030389 | Strain ER01689.3 plasmid unnamed1 | ABS | N/A | ABS | ENA |
| 964 | CP030390 | Strain ER01689.3 | ABS | N/A | ABS | ENA |
| 965 | CP030391 | Strain ER04142.3 | ABS | N/A | ABS | ENA |
| 966 | CP030392 | Strain ER04142.3 plasmid unnamed1 | ABS | N/A | ABS | ENA |
| N/A: No applique, ABS: Absent, NCBI: National Center for Biotechnology Information, ENA: European Nucleotide Archive, *Evidence level 4 and 3, *cas* genes | | | | | | |
| **Supplementary table A. Table of CRISPR-Cas system- and CRISPR-Cas system non-bearing strains of *Staphylococcus aureus* (continuation).** | | | | | | |
| **#** | **ACCESSION NUMBER** | **DESCRIPTION** | **CRISPR-Cas system** | **EVIDENCE LEVEL** | ***cas* GENES** | **DATABASE** |
| 967 | CP030393 | Strain ER02360.3 | ABS | N/A | ABS | ENA |
| 968 | CP030394 | Strain ER01425.3 plasmid unnamed1 | ABS | N/A | ABS | ENA |
| 969 | CP030395 | Strain ER01425.3 | ABS | N/A | ABS | ENA |
| 970 | CP030396 | Strain ER01719.3 | ABS | N/A | ABS | ENA |
| 971 | CP030397 | Strain ER01719.3 plasmid unnamed1 | ABS | N/A | ABS | ENA |
| 972 | CP030398 | Strain ER04612.3 | ABS | N/A | ABS | ENA |
| 973 | CP030399 | Strain ER02637.3 | ABS | N/A | ABS | ENA |
| 974 | CP030400 | Strain ER02637.3 plasmid unnamed1 | ABS | N/A | ABS | ENA |
| 975 | CP030401 | Strain ER02637.3 plasmid unnamed2 | ABS | N/A | ABS | ENA |
| 976 | CP030402 | Strain ER03868.3 plasmid unnamed1 | ABS | N/A | ABS | ENA |
| 977 | CP030403 | Strain ER03868.3 | ABS | N/A | ABS | ENA |
| 978 | CP030404 | Strain ER04219.3 | ABS | N/A | ABS | ENA |
| 979 | CP030405 | Strain ER04219.3 plasmid unnamed1 | ABS | N/A | ABS | ENA |
| 980 | CP030406 | Strain PS00002.3 | ABS | N/A | ABS | ENA |
| 981 | CP030407 | Strain PS00002.3 plasmid unnamed1 | ABS | N/A | ABS | ENA |
| 982 | CP030408 | Strain PS00002.3 plasmid unnamed2 | ABS | N/A | ABS | ENA |
| 983 | CP030409 | Strain ER03996.3 plasmid unnamed1 | ABS | N/A | ABS | ENA |
| 984 | CP030410 | Strain ER03996.3 | ABS | N/A | ABS | ENA |
| 985 | CP030411 | Strain ER04567.3 | ABS | N/A | ABS | ENA |
| 986 | CP030412 | Strain ER02947.3 | ABS | N/A | ABS | ENA |
| 987 | CP030413 | Strain ER02947.3 plasmid unnamed1 | ABS | N/A | ABS | ENA |
| 988 | CP030414 | Strain ER03113.3 plasmid unnamed1 | ABS | N/A | ABS | ENA |
| 989 | CP030415 | Strain ER03113.3 | ABS | N/A | ABS | ENA |
| N/A: No applique, ABS: Absent, NCBI: National Center for Biotechnology Information, ENA: European Nucleotide Archive, *Evidence level 4 and 3, *cas* genes | | | | | | |
| **Supplementary table A. Table of CRISPR-Cas system- and CRISPR-Cas system non-bearing strains of *Staphylococcus aureus* (continuation).** | | | | | | |
| **#** | **ACCESSION NUMBER** | **DESCRIPTION** | **CRISPR-Cas system** | **EVIDENCE LEVEL** | ***cas* GENES** | **DATABASE** |
| 990 | CP030416 | Strain ER01836.3 plasmid unnamed1 | ABS | N/A | ABS | ENA |
| 991 | CP030417 | Strain ER01836.3 plasmid unnamed2 | ABS | N/A | ABS | ENA |
| 992 | CP030418 | Strain ER01836.3 | ABS | N/A | ABS | ENA |
| 993 | CP030419 | Strain ER04115.3 | ABS | N/A | ABS | ENA |
| 994 | CP030420 | Strain ER04115.3 plasmid unnamed1 | ABS | N/A | ABS | ENA |
| 995 | CP030421 | Strain ER04115.3 plasmid unnamed2 | ABS | N/A | ABS | ENA |
| 996 | CP030422 | Strain ER02972.3 | ABS | N/A | ABS | ENA |
| 997 | CP030423 | Strain ER02972.3 plasmid unnamed1 | ABS | N/A | ABS | ENA |
| 998 | CP030424 | Strain ER00551.3 | ABS | N/A | ABS | ENA |
| 999 | CP030425 | Strain ER00551.3 plasmid unnamed1 | ABS | N/A | ABS | ENA |
| 1000 | CP030426 | Strain ER04320.3 | ABS | N/A | ABS | ENA |
| 1001 | CP030427 | Strain ER04320.3 plasmid unnamed1 | ABS | N/A | ABS | ENA |
| 1002 | CP030428 | Strain ER03760.3 | ABS | N/A | ABS | ENA |
| 1003 | CP030429 | Strain ER03760.3 plasmid unnamed1 | ABS | N/A | ABS | ENA |
| 1004 | CP030430 | Strain ER03760.3 plasmid unnamed2 | ABS | N/A | ABS | ENA |
| 1005 | CP030431 | Strain ER04450.3 | ABS | N/A | ABS | ENA |
| 1006 | CP030432 | Strain ER02836.3 | ABS | N/A | ABS | ENA |
| 1007 | CP030433 | Strain ER00610.3 plasmid unnamed1 | ABS | N/A | ABS | ENA |
| 1008 | CP030434 | Strain ER00610.3 | ABS | N/A | ABS | ENA |
| 1009 | CP030435 | Strain ER00610.3 plasmid unnamed2 | ABS | N/A | ABS | ENA |
| 1010 | CP030436 | Strain ER04086.3 plasmid unnamed1 | ABS | N/A | ABS | ENA |
| 1011 | CP030437 | Strain ER04086.3 | ABS | N/A | ABS | ENA |
| 1012 | CP030438 | Strain ER030438.1 | ABS | N/A | ABS | ENA |
| N/A: No applique, ABS: Absent, NCBI: National Center for Biotechnology Information, ENA: European Nucleotide Archive, *Evidence level 4 and 3, *cas* genes | | | | | | |
| **Supplementary table A. Table of CRISPR-Cas system- and CRISPR-Cas system non-bearing strains of *Staphylococcus aureus* (continuation).** | | | | | | |
| **#** | **ACCESSION NUMBER** | **DESCRIPTION** | **CRISPR-Cas system** | **EVIDENCE LEVEL** | ***cas* GENES** | **DATABASE** |
| 1013 | CP030439 | Strain ER02746.3 | ABS | N/A | ABS | ENA |
| 1014 | CP030440 | Strain ER02746.3 plasmid unnamed1 | ABS | N/A | ABS | ENA |
| 1015 | CP030441 | Strain ER04385.3 | ABS | N/A | ABS | ENA |
| 1016 | CP030442 | Strain ER04385.3 plasmid unnamed1 | ABS | N/A | ABS | ENA |
| 1017 | CP030443 | Strain ER01838.3 | ABS | N/A | ABS | ENA |
| 1018 | CP030444 | Strain ER01838.3 plasmid unnamed1 | ABS | N/A | ABS | ENA |
| 1019 | CP030445 | Strain ER04127.3 | ABS | N/A | ABS | ENA |
| 1020 | CP030446 | Strain ER04127.3 plasmid unnamed1 | ABS | N/A | ABS | ENA |
| 1021 | CP030447 | Strain ER04020.4 | ABS | N/A | ABS | ENA |
| 1022 | CP030448 | Strain ER04020.4 plasmid unnamed1 | ABS | N/A | ABS | ENA |
| 1023 | CP030449 | Strain ER04020.4 plasmid unnamed2 | ABS | N/A | ABS | ENA |
| 1024 | CP030450 | Strain ER04225.3 plasmid unnamed1 | ABS | N/A | ABS | ENA |
| 1025 | CP030451 | Strain ER04225.3 | ABS | N/A | ABS | ENA |
| 1026 | CP030452 | Strain PS00001.3 | ABS | N/A | ABS | ENA |
| 1027 | CP030453 | Strain PS00001.3 plasmid unnamed1 | ABS | N/A | ABS | ENA |
| 1028 | CP030454 | Strain PS00001.3 plasmid unnamed2 | ABS | N/A | ABS | ENA |
| 1029 | CP030455 | Strain ER04119.3 | ABS | N/A | ABS | ENA |
| 1030 | CP030456 | Strain ER04119.3 plasmid unnamed1 | ABS | N/A | ABS | ENA |
| 1031 | CP030457 | Strain ER04119.3 plasmid unnamed2 | ABS | N/A | ABS | ENA |
| 1032 | CP030458 | Strain ER04013.3 plasmid unnamed1 | ABS | N/A | ABS | ENA |
| 1033 | CP030459 | Strain ER04013.3 | ABS | N/A | ABS | ENA |
| 1034 | CP030460 | Strain ER04013.3 plasmid unnamed2 | ABS | N/A | ABS | ENA |
| 1035 | CP030461 | Strain ER04013.3 plasmid unnamed3 | ABS | N/A | ABS | ENA |
| N/A: No applique, ABS: Absent, NCBI: National Center for Biotechnology Information, ENA: European Nucleotide Archive, *Evidence level 4 and 3, *cas* genes | | | | | | |
| **Supplementary table A. Table of CRISPR-Cas system- and CRISPR-Cas system non-bearing strains of *Staphylococcus aureus* (continuation).** | | | | | | |
| **#** | **ACCESSION NUMBER** | **DESCRIPTION** | **CRISPR-Cas system** | **EVIDENCE LEVEL** | ***cas* GENES** | **DATABASE** |
| 1036 | CP030462 | Strain ER00695.3 | ABS | N/A | ABS | ENA |
| 1037 | CP030463 | Strain ER00695.3 plasmid unnamed1 | ABS | N/A | ABS | ENA |
| 1038 | CP030464 | Strain ER04261.3 | ABS | N/A | ABS | ENA |
| 1039 | CP030465 | Strain ER04235.3 | ABS | N/A | ABS | ENA |
| 1040 | CP030466 | Strain ER04235.3 plasmid unnamed1 | ABS | N/A | ABS | ENA |
| 1041 | CP030467 | Strain ER03759.3 | ABS | N/A | ABS | ENA |
| 1042 | CP030468 | Strain ER03759.3 plasmid unnamed1 | ABS | N/A | ABS | ENA |
| 1043 | CP030469 | Strain ER03759.3 plasmid unnamed2 | ABS | N/A | ABS | ENA |
| 1044 | CP030470 | Strain ER01109.3 | ABS | N/A | ABS | ENA |
| 1045 | CP030471 | Strain ER01121.3 | ABS | N/A | ABS | ENA |
| 1046 | CP030472 | Strain ER01121.3 plasmid unnamed1 | ABS | N/A | ABS | ENA |
| 1047 | CP030473 | Strain ER00767.3 plasmid unnamed1 | ABS | N/A | ABS | ENA |
| 1048 | CP030474 | Strain ER00767.3 | ABS | N/A | ABS | ENA |
| 1049 | CP030475 | Strain ER03444.3 | ABS | N/A | ABS | ENA |
| 1050 | CP030476 | Strain ER04436.3 | ABS | N/A | ABS | ENA |
| 1051 | CP030477 | Strain ER04436.3 plasmid unnamed1 | ABS | N/A | ABS | ENA |
| 1052 | CP030478 | Strain ER02243.3 plasmid unnamed1 | ABS | N/A | ABS | ENA |
| 1053 | CP030479 | Strain ER02243.3 | ABS | N/A | ABS | ENA |
| 1054 | CP030480 | Strain ER04246.3 plasmid unnamed1 | ABS | N/A | ABS | ENA |
| 1055 | CP030481 | Strain ER04246.3 | ABS | N/A | ABS | ENA |
| 1056 | CP030482 | Strain ER03913.3 plasmid unnamed1 | ABS | N/A | ABS | ENA |
| 1057 | CP030483 | Strain ER03913.3 plasmid unnamed2 | ABS | N/A | ABS | ENA |
| 1058 | CP030484 | Strain ER03913.3 | ABS | N/A | ABS | ENA |
| N/A: No applique, ABS: Absent, NCBI: National Center for Biotechnology Information, ENA: European Nucleotide Archive, *Evidence level 4 and 3, *cas* genes | | | | | | |
| **Supplementary table A. Table of CRISPR-Cas system- and CRISPR-Cas system non-bearing strains of *Staphylococcus aureus* (continuation).** | | | | | | |
| **#** | **ACCESSION NUMBER** | **DESCRIPTION** | **CRISPR-Cas system** | **EVIDENCE LEVEL** | ***cas* GENES** | **DATABASE** |
| 1059 | CP030485 | Strain ER03913.3 plasmid unnamed3 | ABS | N/A | ABS | ENA |
| 1060 | CP030486 | Strain ER03913.3 plasmid unnamed4 | ABS | N/A | ABS | ENA |
| 1061 | CP030487 | Strain ER03913.3 plasmid unnamed5 | ABS | N/A | ABS | ENA |
| 1062 | CP030488 | Strain ER01009.3 plasmid unnamed1 | ABS | N/A | ABS | ENA |
| 1063 | CP030489 | Strain ER01009.3 | ABS | N/A | ABS | ENA |
| 1064 | CP030490 | Strain ER02837.3 | ABS | N/A | ABS | ENA |
| 1065 | CP030491 | Strain ER02837.3 plasmid unnamed1 | ABS | N/A | ABS | ENA |
| 1066 | CP030492 | Strain ER02837.3 plasmid unnamed2 | ABS | N/A | ABS | ENA |
| 1067 | CP030493 | Strain ER00503.3 | ABS | N/A | ABS | ENA |
| 1068 | CP030494 | Strain ER03857.3 plasmid unnamed1 | ABS | N/A | ABS | ENA |
| 1069 | CP030495 | Strain ER03857.3 | ABS | N/A | ABS | ENA |
| 1070 | CP030496 | Strain ER01518.3 | ABS | N/A | ABS | ENA |
| 1071 | CP030497 | Strain ER02658.3 plasmid unnamed1 | ABS | N/A | ABS | ENA |
| 1072 | CP030498 | Strain ER02658.3 | ABS | N/A | ABS | ENA |
| 1073 | CP030499 | Strain ER02612.3 | ABS | N/A | ABS | ENA |
| 1074 | CP030500 | Strain ER02612.3 plasmid unnamed1 | ABS | N/A | ABS | ENA |
| 1075 | CP030501 | Strain ER03762.3 | ABS | N/A | ABS | ENA |
| 1076 | CP030502 | Strain ER03762.3 plasmid unnamed1 | ABS | N/A | ABS | ENA |
| 1077 | CP030503 | Strain ER03762.3 plasmid unnamed2 | ABS | N/A | ABS | ENA |
| 1078 | CP030504 | Strain ER04402.3 plasmid unnamed1 | ABS | N/A | ABS | ENA |
| 1079 | CP030505 | Strain ER04402.3 | ABS | N/A | ABS | ENA |
| 1080 | CP030506 | Strain ER03717.3 | ABS | N/A | ABS | ENA |
| 1081 | CP030507 | Strain ER03717.3 plasmid unnamed1 | ABS | N/A | ABS | ENA |
| N/A: No applique, ABS: Absent, NCBI: National Center for Biotechnology Information, ENA: European Nucleotide Archive, *Evidence level 4 and 3, *cas* genes | | | | | | |
| **Supplementary table A. Table of CRISPR-Cas system- and CRISPR-Cas system non-bearing strains of *Staphylococcus aureus* (continuation).** | | | | | | |
| **#** | **ACCESSION NUMBER** | **DESCRIPTION** | **CRISPR-Cas system** | **EVIDENCE LEVEL** | ***cas* GENES** | **DATABASE** |
| 1082 | CP030508 | Strain ER03717.3 plasmid unnamed2 | ABS | N/A | ABS | ENA |
| 1083 | CP030509 | Strain ER00707.3 plasmid unnamed1 | ABS | N/A | ABS | ENA |
| 1084 | CP030510 | Strain ER00707.3 | ABS | N/A | ABS | ENA |
| 1085 | CP030511 | Strain ER04636.3 plasmid unnamed1 | ABS | N/A | ABS | ENA |
| 1086 | CP030512 | Strain ER04636.3 | ABS | N/A | ABS | ENA |
| 1087 | CP030513 | Strain ER01817.3 plasmid unnamed1 | ABS | N/A | ABS | ENA |
| 1088 | CP030514 | Strain ER01817.3 | ABS | N/A | ABS | ENA |
| 1089 | CP030515 | Strain ER01116.3 plasmid unnamed1 | ABS | N/A | ABS | ENA |
| 1090 | CP030516 | Strain ER01116.3 | ABS | N/A | ABS | ENA |
| 1091 | CP030517 | Strain ER01116.3 plasmid unnamed2 | ABS | N/A | ABS | ENA |
| 1092 | CP030518 | Strain ER04332.2 | ABS | N/A | ABS | ENA |
| 1093 | CP030519 | Strain ER04332.3 plasmid unnamed1 | ABS | N/A | ABS | ENA |
| 1094 | CP030520 | Strain ER01564.3 | ABS | N/A | ABS | ENA |
| 1095 | CP030521 | Strain ER01564.3 plasmid unnamed1 | ABS | N/A | ABS | ENA |
| 1096 | CP030522 | Strain ER01564.3 plasmid unnamed2 | ABS | N/A | ABS | ENA |
| 1097 | CP030523 | Strain ER04163.3 plasmid unnamed1 | ABS | N/A | ABS | ENA |
| 1098 | CP030524 | Strain ER04163.3 | ABS | N/A | ABS | ENA |
| 1099 | CP030525 | Strain ER04174.3 | ABS | N/A | ABS | ENA |
| 1100 | CP030526 | Strain ER02703.3 plasmid unnamed1 | ABS | N/A | ABS | ENA |
| 1101 | CP030527 | Strain ER02703.3 | ABS | N/A | ABS | ENA |
| 1102 | CP030528 | Strain ER02703.3 plasmid unnamed2 | ABS | N/A | ABS | ENA |
| 1103 | CP030529 | Strain ER01776.3 | ABS | N/A | ABS | ENA |
| 1104 | CP030530 | Strain ER04421.3 plasmid unnamed1 | ABS | N/A | ABS | ENA |
| N/A: No applique, ABS: Absent, NCBI: National Center for Biotechnology Information, ENA: European Nucleotide Archive, *Evidence level 4 and 3, *cas* genes | | | | | | |
| **Supplementary table A. Table of CRISPR-Cas system- and CRISPR-Cas system non-bearing strains of *Staphylococcus aureus* (continuation).** | | | | | | |
| **#** | **ACCESSION NUMBER** | **DESCRIPTION** | **CRISPR-Cas system** | **EVIDENCE LEVEL** | ***cas* GENES** | **DATABASE** |
| 1105 | CP030531 | Strain ER04421.3 | ABS | N/A | ABS | ENA |
| 1106 | CP030532 | Strain ER02495.3 | ABS | N/A | ABS | ENA |
| 1107 | CP030533 | Strain ER02495.3 plasmid unnamed1 | ABS | N/A | ABS | ENA |
| 1108 | CP030534 | Strain ER00749.3 | ABS | N/A | ABS | ENA |
| 1109 | CP030535 | Strain ER00749.3 plasmid unnamed1 | ABS | N/A | ABS | ENA |
| 1110 | CP030536 | Strain ER02094.3 plasmid unnamed1 | ABS | N/A | ABS | ENA |
| 1111 | CP030537 | Strain ER02094.3 | ABS | N/A | ABS | ENA |
| 1112 | CP030538 | Strain ER02094.3 plasmid unnamed2 | ABS | N/A | ABS | ENA |
| 1113 | CP030539 | Strain ER01935.3 plasmid unnamed1 | ABS | N/A | ABS | ENA |
| 1114 | CP030540 | Strain ER01935.3 | ABS | N/A | ABS | ENA |
| 1115 | CP030541 | Strain ER04164.3 plasmid unnamed1 | ABS | N/A | ABS | ENA |
| 1116 | CP030542 | Strain ER04164.3 | ABS | N/A | ABS | ENA |
| 1117 | CP030543 | Strain ER04166.3 plasmid unnamed1 | ABS | N/A | ABS | ENA |
| 1118 | CP030544 | Strain ER04166.3 plasmid unnamed2 | ABS | N/A | ABS | ENA |
| 1119 | CP030545 | Strain ER04166.3 | ABS | N/A | ABS | ENA |
| 1120 | CP030546 | Strain ER04181.3 plasmid unnamed1 | ABS | N/A | ABS | ENA |
| 1121 | CP030547 | Strain ER04181.3 | ABS | N/A | ABS | ENA |
| 1122 | CP030548 | Strain ER03928.3 | ABS | N/A | ABS | ENA |
| 1123 | CP030549 | Strain ER03928.3 plasmid unnamed1 | ABS | N/A | ABS | ENA |
| 1124 | CP030550 | Strain ER03364.3 | ABS | N/A | ABS | ENA |
| 1125 | CP030551 | Strain ER03481.3 plasmid unnamed1 | ABS | N/A | ABS | ENA |
| 1126 | CP030552 | Strain ER03481.3 | ABS | N/A | ABS | ENA |
| 1127 | CP030553 | Strain PS00003.3 | ABS | N/A | ABS | ENA |
| N/A: No applique, ABS: Absent, NCBI: National Center for Biotechnology Information, ENA: European Nucleotide Archive, *Evidence level 4 and 3, *cas* genes | | | | | | |
| **Supplementary table A. Table of CRISPR-Cas system- and CRISPR-Cas system non-bearing strains of *Staphylococcus aureus* (continuation).** | | | | | | |
| **#** | **ACCESSION NUMBER** | **DESCRIPTION** | **CRISPR-Cas system** | **EVIDENCE LEVEL** | ***cas* GENES** | **DATABASE** |
| 1128 | CP030554 | Strain PS00003.3 plasmid unnamed1 | ABS | N/A | ABS | ENA |
| 1129 | CP030555 | Strain PS00003.3 plasmid unnamed2 | ABS | N/A | ABS | ENA |
| 1130 | CP030556 | Strain ER03750.3 plasmid unnamed1 | ABS | N/A | ABS | ENA |
| 1131 | CP030557 | Strain ER03750.3 | ABS | N/A | ABS | ENA |
| 1132 | CP030558 | Strain ER04069.3 | ABS | N/A | ABS | ENA |
| 1133 | CP030559 | Strain ER01457.3 plasmid unnamed1 | ABS | N/A | ABS | ENA |
| 1134 | CP030560 | Strain ER01457.3 plasmid unnamed2 | ABS | N/A | ABS | ENA |
| 1135 | CP030561 | Strain ER01457.3 | ABS | N/A | ABS | ENA |
| 1136 | CP030562 | Strain ER03720.3 | ABS | N/A | ABS | ENA |
| 1137 | CP030563 | Strain ER03720.3 plasmid unnamed1 | ABS | N/A | ABS | ENA |
| 1138 | CP030564 | Strain ER01989.3 | ABS | N/A | ABS | ENA |
| 1139 | CP030565 | Strain ER01989.3 plasmid unnamed1 | ABS | N/A | ABS | ENA |
| 1140 | CP030566 | Strain ER03864.3 | ABS | N/A | ABS | ENA |
| 1141 | CP030567 | Strain ER04242.3 plasmid unnamed1 | ABS | N/A | ABS | ENA |
| 1142 | CP030568 | Strain ER04242.3 | ABS | N/A | ABS | ENA |
| 1143 | CP030569 | Strain ER01892.3 plasmid unnamed1 | ABS | N/A | ABS | ENA |
| 1144 | CP030570 | Strain ER01892.3 | ABS | N/A | ABS | ENA |
| 1145 | CP030571 | Strain ER01892.3 plasmid unnamed2 | ABS | N/A | ABS | ENA |
| 1146 | CP030572 | Strain PS00004.3 | ABS | N/A | ABS | ENA |
| 1147 | CP030573 | Strain PS00004.3 plasmid unnamed1 | ABS | N/A | ABS | ENA |
| 1148 | CP030574 | Strain PS00004.3 plasmid unnamed2 | ABS | N/A | ABS | ENA |
| 1149 | CP030575 | Strain ER03910.3 | ABS | N/A | ABS | ENA |
| 1150 | CP030576 | Strain ER03910.3 plasmid unnamed1 | ABS | N/A | ABS | ENA |
| N/A: No applique, ABS: Absent, NCBI: National Center for Biotechnology Information, ENA: European Nucleotide Archive, *Evidence level 4 and 3, *cas* genes | | | | | | |
| **Supplementary table A. Table of CRISPR-Cas system- and CRISPR-Cas system non-bearing strains of *Staphylococcus aureus* (continuation).** | | | | | | |
| **#** | **ACCESSION NUMBER** | **DESCRIPTION** | **CRISPR-Cas system** | **EVIDENCE LEVEL** | ***cas* GENES** | **DATABASE** |
| 1151 | CP030577 | Strain ER01881.3 plasmid unnamed1 | ABS | N/A | ABS | ENA |
| 1152 | CP030578 | Strain ER01881.3 plasmid unnamed2 | ABS | N/A | ABS | ENA |
| 1153 | CP030579 | Strain ER01881.3 | ABS | N/A | ABS | ENA |
| 1154 | CP030580 | Strain ER04397.3 | ABS | N/A | ABS | ENA |
| 1155 | CP030581 | Strain ER04397.3 plasmid unnamed1 | ABS | N/A | ABS | ENA |
| 1156 | CP030582 | Strain ER04397.3 plasmid unnamed2 | ABS | N/A | ABS | ENA |
| 1157 | CP030583 | Strain ER02262.3 | ABS | N/A | ABS | ENA |
| 1158 | CP030584 | Strain ER02262.3 plasmid unnamed1 | ABS | N/A | ABS | ENA |
| 1159 | CP030585 | Strain ER01073.3 | ABS | N/A | ABS | ENA |
| 1160 | CP030586 | Strain ER00594.3 | ABS | N/A | ABS | ENA |
| 1161 | CP030587 | Strain ER00594.3 plasmid unnamed1 | ABS | N/A | ABS | ENA |
| 1162 | CP030588 | Strain ER02988.3 | ABS | N/A | ABS | ENA |
| 1163 | CP030589 | Strain ER04314.3 plasmid unnamed1 | ABS | N/A | ABS | ENA |
| 1164 | CP030590 | Strain ER04314.3 | ABS | N/A | ABS | ENA |
| 1165 | CP030591 | Strain ER02989.3 plasmid unnamed1 | ABS | N/A | ABS | ENA |
| 1166 | CP030592 | Strain ER02989.3 | ABS | N/A | ABS | ENA |
| 1167 | CP030593 | Strain ER02989.3 plasmid unnamed2 | ABS | N/A | ABS | ENA |
| 1168 | CP030594 | Strain ER03588.3 plasmid unnamed1 | ABS | N/A | ABS | ENA |
| 1169 | CP030595 | Strain ER03588.3 | ABS | N/A | ABS | ENA |
| 1170 | CP030596 | Strain ER02443.3 | ABS | N/A | ABS | ENA |
| 1171 | CP030597 | Strain ER02443.3 plasmid unnamed1 | ABS | N/A | ABS | ENA |
| 1172 | CP030598 | Strain ER02524.3 plasmid unnamed1 | ABS | N/A | ABS | ENA |
| 1173 | CP030599 | Strain ER02524.3 | ABS | N/A | ABS | ENA |
| N/A: No applique, ABS: Absent, NCBI: National Center for Biotechnology Information, ENA: European Nucleotide Archive, *Evidence level 4 and 3, *cas* genes | | | | | | |
| **Supplementary table A. Table of CRISPR-Cas system- and CRISPR-Cas system non-bearing strains of *Staphylococcus aureus* (continuation).** | | | | | | |
| **#** | **ACCESSION NUMBER** | **DESCRIPTION** | **CRISPR-Cas system** | **EVIDENCE LEVEL** | ***cas* GENES** | **DATABASE** |
| 1174 | CP030600 | Strain ER00658.3 plasmid unnamed1 | ABS | N/A | ABS | ENA |
| 1175 | CP030601 | Strain ER00658.3 | ABS | N/A | ABS | ENA |
| 1176 | CP030602 | Strain ER00658.3 plasmid unnamed2 | ABS | N/A | ABS | ENA |
| 1177 | CP030603 | Strain ER00484.3 plasmid unnamed1 | ABS | N/A | ABS | ENA |
| 1178 | CP030604 | Strain ER00484.3 | ABS | N/A | ABS | ENA |
| 1179 | CP030605 | Strain ER02693.3 | ABS | N/A | ABS | ENA |
| 1180 | CP030606 | Strain ER02693.3 plasmid unnamed1 | ABS | N/A | ABS | ENA |
| 1181 | CP030608 | Strain ER04021.4 | ABS | N/A | ABS | ENA |
| 1182 | CP030609 | Strain ER04021.4 plasmid unnamed1 | ABS | N/A | ABS | ENA |
| 1183 | CP030610 | Strain ER04021.4 plasmid unnamed2 | ABS | N/A | ABS | ENA |
| 1184 | CP030611 | Strain ER03930.3 | ABS | N/A | ABS | ENA |
| 1185 | CP030612 | Strain ER03930.3 plasmid unnamed1 | ABS | N/A | ABS | ENA |
| 1186 | CP030613 | Strain ER03930.3 plasmid unnamed2 | ABS | N/A | ABS | ENA |
| 1187 | CP030614 | Strain ER01560.3 plasmid unnamed1 | ABS | N/A | ABS | ENA |
| 1188 | CP030615 | Strain ER01560.3 | ABS | N/A | ABS | ENA |
| 1189 | CP030616 | Strain ER01560.3 plasmid unnamed2 | ABS | N/A | ABS | ENA |
| 1190 | CP030617 | Strain ER04440.3 | ABS | N/A | ABS | ENA |
| 1191 | CP030618 | Strain ER04440.3 plasmid unnamed1 | ABS | N/A | ABS | ENA |
| 1192 | CP030619 | Strain ER04440.3 plasmid unnamed2 | ABS | N/A | ABS | ENA |
| 1193 | CP030620 | Strain ER01524.3 plasmid unnamed1 | ABS | N/A | ABS | ENA |
| 1194 | CP030621 | Strain ER01524.3 | ABS | N/A | ABS | ENA |
| 1195 | CP030622 | Strain ER01524.3 plasmid unnamed2 | ABS | N/A | ABS | ENA |
| 1196 | CP030623 | Strain ER01524.3 plasmid unnamed3 | ABS | N/A | ABS | ENA |
| N/A: No applique, ABS: Absent, NCBI: National Center for Biotechnology Information, ENA: European Nucleotide Archive, *Evidence level 4 and 3, *cas* genes | | | | | | |
| **Supplementary table A. Table of CRISPR-Cas system- and CRISPR-Cas system non-bearing strains of *Staphylococcus aureus* (continuation).** | | | | | | |
| **#** | **ACCESSION NUMBER** | **DESCRIPTION** | **CRISPR-Cas system** | **EVIDENCE LEVEL** | ***cas* GENES** | **DATABASE** |
| 1197 | CP030624 | Strain ER03448.3 | ABS | N/A | ABS | ENA |
| 1198 | CP030625 | Strain ER03448.3 plasmid unnamed1 | ABS | N/A | ABS | ENA |
| 1199 | CP030626 | Strain ER01823.3 | ABS | N/A | ABS | ENA |
| 1200 | CP030627 | Strain ER01823.3 plasmid unnamed1 | ABS | N/A | ABS | ENA |
| 1201 | CP030628 | Strain ER03809.3 | ABS | N/A | ABS | ENA |
| 1202 | CP030629 | Strain ER03809.3 plasmid unnamed1 | ABS | N/A | ABS | ENA |
| 1203 | CP030630 | Strain ER03544.3 | ABS | N/A | ABS | ENA |
| 1204 | CP030631 | Strain ER03544.3 plasmid unnamed1 | ABS | N/A | ABS | ENA |
| 1205 | CP030632 | Strain ER03544.3 plasmid unnamed2 | ABS | N/A | ABS | ENA |
| 1206 | CP030633 | Strain ER03755.3 plasmid unnamed1 | ABS | N/A | ABS | ENA |
| 1207 | CP030634 | Strain ER03755.3 | ABS | N/A | ABS | ENA |
| 1208 | CP030635 | Strain ER04085.3 | ABS | N/A | ABS | ENA |
| 1209 | CP030636 | Strain ER04085.3 plasmid unnamed1 | ABS | N/A | ABS | ENA |
| 1210 | CP030637 | Strain ER04085.3 plasmid unnamed2 | ABS | N/A | ABS | ENA |
| 1211 | CP030638 | Strain ER04165.3 | ABS | N/A | ABS | ENA |
| 1212 | CP030639 | Strain ER04165.3 plasmid unnamed1 | ABS | N/A | ABS | ENA |
| 1213 | CP030640 | Strain ER04165.3 plasmid unnamed2 | ABS | N/A | ABS | ENA |
| 1214 | CP030641 | Strain ER03737.3 | ABS | N/A | ABS | ENA |
| 1215 | CP030642 | Strain ER00385.3 | ABS | N/A | ABS | ENA |
| 1216 | CP030643 | Strain ER00385.3 plasmid unnamed1 | ABS | N/A | ABS | ENA |
| 1217 | CP030644 | Strain ER03298.3 plasmid unnamed1 | ABS | N/A | ABS | ENA |
| 1218 | CP030645 | Strain ER03298.3 | ABS | N/A | ABS | ENA |
| 1219 | CP030646 | Strain ER03710.3 | ABS | N/A | ABS | ENA |
| N/A: No applique, ABS: Absent, NCBI: National Center for Biotechnology Information, ENA: European Nucleotide Archive, *Evidence level 4 and 3, *cas* genes | | | | | | |
| **Supplementary table A. Table of CRISPR-Cas system- and CRISPR-Cas system non-bearing strains of *Staphylococcus aureus* (continuation).** | | | | | | |
| **#** | **ACCESSION NUMBER** | **DESCRIPTION** | **CRISPR-Cas system** | **EVIDENCE LEVEL** | ***cas* GENES** | **DATABASE** |
| 1220 | CP030647 | Strain ER03556.3 | ABS | N/A | ABS | ENA |
| 1221 | CP030648 | Strain ER03556.3 plasmid unnamed1 | ABS | N/A | ABS | ENA |
| 1222 | CP030649 | Strain ER03556.3 plasmid unnamed2 | ABS | N/A | ABS | ENA |
| 1223 | CP030650 | Strain ER04324.3 | ABS | N/A | ABS | ENA |
| 1224 | CP030651 | Strain ER04324.3 plasmid unnamed1 | ABS | N/A | ABS | ENA |
| 1225 | CP030652 | Strain ER04324.3 plasmid unnamed2 | ABS | N/A | ABS | ENA |
| 1226 | CP030653 | Strain ER01570.3 | ABS | N/A | ABS | ENA |
| 1227 | CP030654 | Strain ER01746.3 | ABS | N/A | ABS | ENA |
| 1228 | CP030655 | Strain ER04448.3 plasmid unnamed1 | ABS | N/A | ABS | ENA |
| 1229 | CP030656 | Strain ER04448.3 | ABS | N/A | ABS | ENA |
| 1230 | CP030657 | Strain ER04448.3 plasmid unnamed2 | ABS | N/A | ABS | ENA |
| 1231 | CP030658 | Strain ER02969.3 | ABS | N/A | ABS | ENA |
| 1232 | CP030659 | Strain ER02217.3 | ABS | N/A | ABS | ENA |
| 1233 | CP030660 | Strain ER02217.3 plasmid unnamed1 | ABS | N/A | ABS | ENA |
| 1234 | CP030661 | Strain ER02826.3 | ABS | N/A | ABS | ENA |
| 1235 | CP030662 | Strain ER02826.3 plasmid unnamed1 | ABS | N/A | ABS | ENA |
| 1236 | CP030663 | Strain ER03489.3 plasmid unnamed1 | ABS | N/A | ABS | ENA |
| 1237 | CP030663 | Strain ER03489.3 plasmid unnamed1 | ABS | N/A | ABS | ENA |
| 1238 | CP030664 | Strain ER03489.3 | ABS | N/A | ABS | ENA |
| 1239 | CP030665 | Strain ER01454.3 | ABS | N/A | ABS | ENA |
| 1240 | CP030666 | Strain ER01454.3 plasmid unnamed1 | ABS | N/A | ABS | ENA |
| 1241 | CP030667 | Strain ER02069.3 plasmid unnamed1 | ABS | N/A | ABS | ENA |
| 1242 | CP030668 | Strain ER02069.3 | ABS | N/A | ABS | ENA |
| N/A: No applique, ABS: Absent, NCBI: National Center for Biotechnology Information, ENA: European Nucleotide Archive, *Evidence level 4 and 3, *cas* genes | | | | | | |
| **Supplementary table A. Table of CRISPR-Cas system- and CRISPR-Cas system non-bearing strains of *Staphylococcus aureus* (continuation).** | | | | | | |
| **#** | **ACCESSION NUMBER** | **DESCRIPTION** | **CRISPR-Cas system** | **EVIDENCE LEVEL** | ***cas* GENES** | **DATABASE** |
| 1243 | CP030669 | Strain ER00951.3 plasmid unnamed1 | ABS | N/A | ABS | ENA |
| 1244 | CP030670 | Strain ER00951.3 | ABS | N/A | ABS | ENA |
| 1245 | CP030671 | Strain ER00951.3 plasmid unnamed2 | ABS | N/A | ABS | ENA |
| 1246 | CP030672 | Strain pt053 plasmid unnamed1 | ABS | N/A | ABS | ENA |
| 1247 | CP030673 | Strain pt053 | ABS | N/A | ABS | ENA |
| 1248 | CP030674 | Strain ER01533.3 plasmid unnamed1 | ABS | N/A | ABS | ENA |
| 1249 | CP030675 | Strain ER01533.3 | ABS | N/A | ABS | ENA |
| 1250 | CP030676 | Strain ER01533.3 plasmid unnamed2 | ABS | N/A | ABS | ENA |
| 1251 | CP030677 | Strain ER01533.3 plasmid unnamed3 | ABS | N/A | ABS | ENA |
| 1252 | CP030678 | Strain ER00573.3 plasmid unnamed1 | ABS | N/A | ABS | ENA |
| 1253 | CP030679 | Strain ER00573.3 plasmid unnamed2 | ABS | N/A | ABS | ENA |
| 1254 | CP030680 | Strain ER00573.3 plasmid unnamed3 | ABS | N/A | ABS | ENA |
| 1255 | CP030681 | Strain ER00573.3 | ABS | N/A | ABS | ENA |
| 1256 | CP030682 | Strain ER05857.3 plasmid unnamed1 | ABS | N/A | ABS | ENA |
| 1257 | CP030683 | Strain ER05857.3 | ABS | N/A | ABS | ENA |
| 1258 | CP030684 | Strain ER04407.3 | ABS | N/A | ABS | ENA |
| 1259 | CP030685 | Strain ER04407.3 plasmid unnamed1 | ABS | N/A | ABS | ENA |
| 1260 | CP030686 | Strain ER04407.3 plasmid unnamed2 | ABS | N/A | ABS | ENA |
| 1261 | CP030687 | Strain ER02919.3 plasmid unnamed1 | ABS | N/A | ABS | ENA |
| 1262 | CP030688 | Strain ER02919.3 | ABS | N/A | ABS | ENA |
| 1263 | CP030689 | Strain ER01507.3 | ABS | N/A | ABS | ENA |
| 1264 | CP030690 | Strain ER01507.3 plasmid unnamed1 | ABS | N/A | ABS | ENA |
| 1265 | CP030691 | Strain ER01422.3 plasmid unnamed1 | ABS | N/A | ABS | ENA |
| N/A: No applique, ABS: Absent, NCBI: National Center for Biotechnology Information, ENA: European Nucleotide Archive, *Evidence level 4 and 3, *cas* genes | | | | | | |
| **Supplementary table A. Table of CRISPR-Cas system- and CRISPR-Cas system non-bearing strains of *Staphylococcus aureus* (continuation).** | | | | | | |
| **#** | **ACCESSION NUMBER** | **DESCRIPTION** | **CRISPR-Cas system** | **EVIDENCE LEVEL** | ***cas* GENES** | **DATABASE** |
| 1266 | CP030692 | Strain ER01422.3 | ABS | N/A | ABS | ENA |
| 1267 | CP030693 | Strain ER01803.3 | ABS | N/A | ABS | ENA |
| 1268 | CP030694 | Strain ER01803.3 plasmid unnamed1 | ABS | N/A | ABS | ENA |
| 1269 | CP030695 | Strain ER01532.3 | ABS | N/A | ABS | ENA |
| 1270 | CP030696 | Strain ER01532.3 plasmid unnamed1 | ABS | N/A | ABS | ENA |
| 1271 | CP030697 | Strain ER01532.3 plasmid unnamed2 | ABS | N/A | ABS | ENA |
| 1272 | CP030698 | Strain ER01334.3 plasmid unnamed1 | ABS | N/A | ABS | ENA |
| 1273 | CP030699 | Strain ER01334.3 | ABS | N/A | ABS | ENA |
| 1274 | CP030700 | Strain ER03023.3 plasmid unnamed1 | ABS | N/A | ABS | ENA |
| 1275 | CP030701 | Strain ER03023.3 | ABS | N/A | ABS | ENA |
| 1276 | CP030702 | Strain ER03321.3 | ABS | N/A | ABS | ENA |
| 1277 | CP030703 | Strain ER03321.3 plasmid unnamed1 | ABS | N/A | ABS | ENA |
| 1278 | CP030704 | Strain ER03763.3 | ABS | N/A | ABS | ENA |
| 1279 | CP030705 | Strain ER03763.3 plasmid unnamed1 | ABS | N/A | ABS | ENA |
| 1280 | CP030706 | Strain ER03763.3 plasmid unnamed2 | ABS | N/A | ABS | ENA |
| 1281 | CP030707 | Strain ER03493.3 plasmid unnamed1 | ABS | N/A | ABS | ENA |
| 1282 | CP030708 | Strain ER03493.3 | ABS | N/A | ABS | ENA |
| 1283 | CP030709 | Strain ER04060.3 | ABS | N/A | ABS | ENA |
| 1284 | CP030710 | Strain ER01174.3 plasmid unnamed1 | ABS | N/A | ABS | ENA |
| 1285 | CP030711 | Strain ER01174.3 plasmid unnamed2 | ABS | N/A | ABS | ENA |
| 1286 | CP030712 | Strain ER01174.3 | ABS | N/A | ABS | ENA |
| 1287 | CP030713 | Strain ER02878.3 | ABS | N/A | ABS | ENA |
| 1288 | CP030714 | Strain ER02878.3 plasmid unnamed1 | ABS | N/A | ABS | ENA |
| N/A: No applique, ABS: Absent, NCBI: National Center for Biotechnology Information, ENA: European Nucleotide Archive, *Evidence level 4 and 3, *cas* genes | | | | | | |
| **Supplementary table A. Table of CRISPR-Cas system- and CRISPR-Cas system non-bearing strains of *Staphylococcus aureus* (continuation).** | | | | | | |
| **#** | **ACCESSION NUMBER** | **DESCRIPTION** | **CRISPR-Cas system** | **EVIDENCE LEVEL** | ***cas* GENES** | **DATABASE** |
| 1289 | CP041746 | Strain 6538P | ABS | N/A | ABS | ENA |
| 1290 | CP042008 | Strain B3-4A | ABS | N/A | ABS | ENA |
| 1291 | CP042048 | Strain B1-4A | ABS | N/A | ABS | ENA |
| 1292 | CP042081 | Strain B9-22D | ABS | N/A | ABS | ENA |
| 1293 | CP042110 | Strain B6-55A | ABS | N/A | ABS | ENA |
| 1294 | CP043843 | Strain NCCP 16830 | ABS | N/A | ABS | ENA |
| 1295 | CP045468 | Strain SAW1 | ABS | N/A | ABS | ENA |
| 1296 | CP047021 | Strain M2024 | ABS | N/A | ABS | ENA |
| 1297 | CP047321 | Strain RJ1267 | ABS | N/A | ABS | ENA |
| 1298 | CP047322 | Strain RJ1267 plasmid unnamed | ABS | N/A | ABS | ENA |
| 1299 | CP047777 | Strain UP_1632 | ABS | N/A | ABS | ENA |
| 1300 | CP047778 | Strain UP_1572 | ABS | N/A | ABS | ENA |
| 1301 | CP047779 | Strain UP_1559 | ABS | N/A | ABS | ENA |
| 1302 | CP047780 | Strain UP_1539 | ABS | N/A | ABS | ENA |
| 1303 | CP047781 | Strain UP_1452 | ABS | N/A | ABS | ENA |
| 1304 | CP047782 | Strain UP_1352 | ABS | N/A | ABS | ENA |
| 1305 | CP047783 | Strain UP_1313 | ABS | N/A | ABS | ENA |
| 1306 | CP047784 | Strain UP_1209 | ABS | N/A | ABS | ENA |
| 1307 | CP047785 | Strain UP_1186 | ABS | N/A | ABS | ENA |
| 1308 | CP047786 | Strain UP_1150 | ABS | N/A | ABS | ENA |
| 1309 | CP047787 | Strain UP_1108 | ABS | N/A | ABS | ENA |
| 1310 | CP047788 | Strain UP_1033 | ABS | N/A | ABS | ENA |
| 1311 | CP047789 | Strain UP_967 | ABS | N/A | ABS | ENA |
| N/A: No applique, ABS: Absent, NCBI: National Center for Biotechnology Information, ENA: European Nucleotide Archive, *Evidence level 4 and 3, *cas* genes | | | | | | |
| **Supplementary table A. Table of CRISPR-Cas system- and CRISPR-Cas system non-bearing strains of *Staphylococcus aureus* (continuation).** | | | | | | |
| **#** | **ACCESSION NUMBER** | **DESCRIPTION** | **CRISPR-Cas system** | **EVIDENCE LEVEL** | ***cas* GENES** | **DATABASE** |
| 1312 | CP047790 | Strain UP_830 | ABS | N/A | ABS | ENA |
| 1313 | CP047791 | Strain UP_818 | ABS | N/A | ABS | ENA |
| 1314 | CP047792 | Strain UP_794 | ABS | N/A | ABS | ENA |
| 1315 | CP047793 | Strain UP_764 | ABS | N/A | ABS | ENA |
| 1316 | CP047794 | Strain UP_551 | ABS | N/A | ABS | ENA |
| 1317 | CP047795 | Strain UP_490 | ABS | N/A | ABS | ENA |
| 1318 | CP047796 | Strain UP_462 | ABS | N/A | ABS | ENA |
| 1319 | CP047797 | Strain UP_426 | ABS | N/A | ABS | ENA |
| 1320 | CP047798 | Strain UP_378 | ABS | N/A | ABS | ENA |
| 1321 | CP047799 | Strain UP_322 | ABS | N/A | ABS | ENA |
| 1322 | CP047800 | Strain UP_296 | ABS | N/A | ABS | ENA |
| 1323 | CP047801 | Strain UP_248 | ABS | N/A | ABS | ENA |
| 1324 | CP047802 | Strain UP_1442 | ABS | N/A | ABS | ENA |
| 1325 | CP047803 | Strain UP_1097 | ABS | N/A | ABS | ENA |
| 1326 | CP047804 | Strain UP_774 | ABS | N/A | ABS | ENA |
| 1327 | CP047805 | Strain UP_1654 | ABS | N/A | ABS | ENA |
| 1328 | CP047807 | Strain UP_1612 | ABS | N/A | ABS | ENA |
| 1329 | CP047809 | Strain UP_1591 | ABS | N/A | ABS | ENA |
| 1330 | CP047811 | Strain UP_1522 | ABS | N/A | ABS | ENA |
| 1331 | CP047813 | Strain UP_1500 | ABS | N/A | ABS | ENA |
| 1332 | CP047815 | Strain UP_1484 | ABS | N/A | ABS | ENA |
| 1333 | CP047817 | Strain UP_1433 | ABS | N/A | ABS | ENA |
| 1334 | CP047820 | Strain UP_1395 | ABS | N/A | ABS | ENA |
| N/A: No applique, ABS: Absent, NCBI: National Center for Biotechnology Information, ENA: European Nucleotide Archive, *Evidence level 4 and 3, *cas* genes | | | | | | |
| **Supplementary table A. Table of CRISPR-Cas system- and CRISPR-Cas system non-bearing strains of *Staphylococcus aureus* (continuation).** | | | | | | |
| **#** | **ACCESSION NUMBER** | **DESCRIPTION** | **CRISPR-Cas system** | **EVIDENCE LEVEL** | ***cas* GENES** | **DATABASE** |
| 1335 | CP047822 | Strain UP_1278 | ABS | N/A | ABS | ENA |
| 1336 | CP047826 | Strain UP_1239 | ABS | N/A | ABS | ENA |
| 1337 | CP047828 | Strain UP_1073 | ABS | N/A | ABS | ENA |
| 1338 | CP047830 | Strain UP_996 | ABS | N/A | ABS | ENA |
| 1339 | CP047833 | Strain UP_926 | ABS | N/A | ABS | ENA |
| 1340 | CP047835 | Strain UP_883 | ABS | N/A | ABS | ENA |
| 1341 | CP047837 | Strain UP_719 | ABS | N/A | ABS | ENA |
| 1342 | CP047839 | Strain UP_678 | ABS | N/A | ABS | ENA |
| 1343 | CP047841 | Strain UP_644 | ABS | N/A | ABS | ENA |
| 1344 | CP047843 | Strain UP_620 | ABS | N/A | ABS | ENA |
| 1345 | CP047845 | Strain UP_591 | ABS | N/A | ABS | ENA |
| 1346 | CP047847 | Strain UP_522 | ABS | N/A | ABS | ENA |
| 1347 | CP047849 | Strain UP_403 | ABS | N/A | ABS | ENA |
| 1348 | CP047851 | Strain UP_338 | ABS | N/A | ABS | ENA |
| 1349 | CP047852 | Strain UP_274 | ABS | N/A | ABS | ENA |
| 1350 | CP047854 | Strain UP_1525 | ABS | N/A | ABS | ENA |
| 1351 | CP047856 | Strain UP_1435 | ABS | N/A | ABS | ENA |
| 1352 | CP047859 | Strain UP_1405 | ABS | N/A | ABS | ENA |
| 1353 | CP047861 | Strain UP_1322 | ABS | N/A | ABS | ENA |
| 1354 | CP047863 | Strain UP_1106 | ABS | N/A | ABS | ENA |
| 1355 | CP047865 | Strain UP_844 | ABS | N/A | ABS | ENA |
| 1356 | CP047867 | Strain UP_419 | ABS | N/A | ABS | ENA |
| 1357 | CP048431 | Strain SA1428 | ABS | N/A | ABS | ENA |
| N/A: No applique, ABS: Absent, NCBI: National Center for Biotechnology Information, ENA: European Nucleotide Archive, *Evidence level 4 and 3, *cas* genes | | | | | | |
| **Supplementary table A. Table of CRISPR-Cas system- and CRISPR-Cas system non-bearing strains of *Staphylococcus aureus* (continuation).** | | | | | | |
| **#** | **ACCESSION NUMBER** | **DESCRIPTION** | **CRISPR-Cas system** | **EVIDENCE LEVEL** | ***cas* GENES** | **DATABASE** |
| 1358 | CP048643 | Strain SR153 | ABS | N/A | ABS | ENA |
| 1359 | FM207042 | Plasmid pGO1 | ABS | N/A | ABS | ENA |
| 1360 | HF937103 | Strain M1 | ABS | N/A | ABS | ENA |
| 1361 | HF937104 | Strain M1 plasmid pSK67-M1 | ABS | N/A | ABS | ENA |
| 1362 | LC012933 | Strain OC160 plasmid pOC160-2 | ABS | N/A | ABS | ENA |
| 1363 | M19652 | Strain T48 plasmid pT48 | ABS | N/A | ABS | ENA |
| 1364 | MF706324 | Strain 4185 plasmid pRJ101 | ABS | N/A | ABS | ENA |
| 1365 | U96609 | Plasmid pSK3 | ABS | N/A | ABS | ENA |
| 1366 | U96610 | Plasmid pSK6 | ABS | N/A | ABS | ENA |
| 1367 | FN377602 | Strain ST398 multiresistance plasmid pKKS825 | ABS | N/A | ABS | ENA |
| 1368 | NZ_CP038183 | Strain MJ015 | ABS | N/A | ABS | NCBI |
| 1369 | NZ_CP038184 | Strain MJ015 plasmid unnamed1 | ABS | N/A | ABS | NCBI |
| 1370 | NZ_CP038185 | Strain MJ015 plasmid unnamed2 | ABS | N/A | ABS | NCBI |
| 1371 | NZ_CP050690 | Strain PMB179-1 | ABS | N/A | ABS | NCBI |
| 1372 | NZ_CP050691 | Strain PMB196-1 | ABS | N/A | ABS | NCBI |
| 1373 | NZ_CP053353 | Strain 16405 | ABS | N/A | ABS | NCBI |
| 1374 | NZ_CP053354 | Strain 16405 plasmid unnamed | ABS | N/A | ABS | NCBI |
| 1375 | NZ_CP053355 | Strain 14507 plasmid unnamed | ABS | N/A | ABS | NCBI |
| 1376 | NZ_CP053356 | Strain 14507 | ABS | N/A | ABS | NCBI |
| 1377 | NZ_CP053634 | Strain 14638 | ABS | N/A | ABS | NCBI |
| 1378 | NZ_CP053635 | Strain 14638 plasmid unnamed | ABS | N/A | ABS | NCBI |
| 1379 | NZ_CP053636 | Strain 14640 | ABS | N/A | ABS | NCBI |
| 1380 | NZ_CP053637 | Strain 14640 plasmid unnamed | ABS | N/A | ABS | NCBI |
| N/A: No applique, ABS: Absent, NCBI: National Center for Biotechnology Information, ENA: European Nucleotide Archive, *Evidence level 4 and 3, *cas* genes | | | | | | |
| **Supplementary table A. Table of CRISPR-Cas system- and CRISPR-Cas system non-bearing strains of *Staphylococcus aureus* (continuation).** | | | | | | |
| **#** | **ACCESSION NUMBER** | **DESCRIPTION** | **CRISPR-Cas system** | **EVIDENCE LEVEL** | ***cas* GENES** | **DATABASE** |
| 1381 | NZ_CP053638 | Strain 14732 plasmid unnamed | ABS | N/A | ABS | NCBI |
| 1382 | NZ_CP053639 | Strain 14732 | ABS | N/A | ABS | NCBI |
| 1383 | NZ_CP053640 | Strain 14505 | ABS | N/A | ABS | NCBI |
| 1384 | NZ_CP053641 | Strain 14505 plasmid unnamed | ABS | N/A | ABS | NCBI |
| 1385 | NZ_CP054876 | Subsp. *aureus* Strain Dresden-275757 | ABS | N/A | ABS | NCBI |
| N/A: No applique, ABS: Absent, NCBI: National Center for Biotechnology Information, ENA: European Nucleotide Archive, *Evidence level 4 and 3, *cas* genes | | | | | | |
