# Supplementary material for: Identification and Characterization of the CRISPR/Cas System in Staphylococcus aureus Strains From Diverse Sources
Source: Front Microbiol. 2021 Jun 2;12:656996. doi: 10.3389/fmicb.2021.656996 (PMC8206494; doi:10.3389/fmicb.2021.656996)
Supplement: Supplementary file 2 [file Table_2.DOCX]

Supplementary table B. Genomics characteristics of the CRISPR-Cas systems founded in *Staphylococcus aureus* strains.

This supplementary table content the characteristics of the CRISPR-Cas system, all of them are obtained from server web CRISPRCasfinder and they were put together at the next tables.

| Additional information B. Genomics characteristics of CRISPR-Cas systems founded in *Staphylococcus aureus* strains. | | | | | | | | |
| --- | --- | --- | --- | --- | --- | --- | --- | --- |
| Supplementary table A* | Database | Accession number | Description | CRISPR-Cas system | Start locus CRISPR | End locus CRISPR | Spacer sequence | DR consensus (Repeat sequence consensus) |
| 57 | NCBI | NC_018608 | 08BA02176 | III-A | 55513 | 56620 | 15 | GATCGATAACTACCCCGAATAACAGGGGACGAGAAT |
| 121 | NCBI | NZ_AP020315 | KUH140087 | III-A | 1951227 | 1951627 | 5 | GTTCTCGTCCCCTCTTCTTCGGGGTAGTTATCGAAT |
| 201 | NCBI | NZ_CP012756 | Subsp. *aureus* cepa JS395 | III-A | 1407844 | 1408306 | 6 | GATCGATAACTACCCCGAATAACAGGGGACGAGAATA |
| 530 | NCBI | NZ_CP029649 | AR 0472 | III-A | 1734173 | 1735063 | 12 | ATTCTCGTCCCCTGTTATTCGGGGTAGTTATCGATC |
| 534 | NCBI | NZ_CP029653 | AR 0470 | III-A | 61276 | 62170 | 12 | GATCGATAACTACCCCGAATAACAGGGGACGAGAATT |
| 561 | NCBI | NZ_CP029681 | AR 0473 | III-A | 2360243 | 2361133 | 12 | ATTCTCGTCCCCTGTTATTCGGGGTAGTTATCGATC |
| *Row number at supplementary table A, pb: pair base number, DR: repeat sequence consensus, NCBI: National Center for Biotechnology Information | | | | | | | | |

| Additional information B. Genomics characteristics of CRISPR-Cas systems founded in *Staphylococcus aureus* strains. | | | | | | | | | |
| --- | --- | --- | --- | --- | --- | --- | --- | --- | --- |
| Supplementary table A* | Database | Number accession | Description | Length DR | DR homology (%) | Spacer sequence homology (%) | Direction | Orientation (%AT) | LE |
| 57 | NCBI | NC_018608 | 08BA02176 | 36 | 95.80 | 0.00 | + | - | 4 |
| 121 | NCBI | NZ_AP020315 | KUH140087 | 36 | 89.30 | 9.52 | ND | - | 3 |
| 201 | NCBI | NZ_CP012756 | Subsp. a*ureus* strain JS395 | 37 | 89.43 | 4.26 | + | - | 4 |
| 530 | NCBI | NZ_CP029649 | AR 0472 | 36 | 95.15 | 0.00 | ND | + | 4 |
| 534 | NCBI | NZ_CP029653 | AR 0470 | 37 | 87.47 | 0.00 | ND | - | 4 |
| 561 | NCBI | NZ_CP02968 | AR 0473 | 36 | 95.15 | 0.00 | ND | + | 4 |
| *Row number at supplementary table A, LE: evidence level, DR: repeat sequence consensus, ND: Non-determinate. | | | | | | | | | |
