# Supplementary material for: Identification and Characterization of the CRISPR/Cas System in Staphylococcus aureus Strains From Diverse Sources
Source: Front Microbiol. 2021 Jun 2;12:656996. doi: 10.3389/fmicb.2021.656996 (PMC8206494; doi:10.3389/fmicb.2021.656996)
Supplement: Supplementary file 3 [file Table_3.DOCX]

Supplementary table C. Spacer sequence (SS) of locus CRISPR from *Staphylococcus aureus* matching to mobile genetic element (MGE) known.

This supplementary table contains the parameters of BLAST analysis to each spacer sequence (SS). The SS was submitted to alignment with BLAST to find a possible match with a mobile genetic element (MGE) known. At first column content name of CRISPR-Cas system-bearing strain, at second column are the nucleotide sequence of each SS, at the third column is MGE that make a match with SS, at the fourth column are shown max score for each alignment, at the fifth column are shown total score for each alignment, at sixth column are shown cover percentage for each alignment, at seventh column are shown e-value for each alignment, at the eighth column are shown the identity percentage and at the ninth column are indicated the accession number of MGE.

| Supplementary table C. Spacer sequence (SS) from each CRISPR-Cas system-bearing strain of *Staphylococcus aureus* associated with a mobile genetic element (MGE) known. | | | | | | | | |
| --- | --- | --- | --- | --- | --- | --- | --- | --- |
| Strain | Spacer sequence | MGE | Max score | Total score | Coverage (%) | e-value | Identity (%) | Accession number |
| 08BA02176 | TCTATAAGTTCATTAATTCCGATACCTAGATTATCT | NR | NA | NA | NA | NA | NA | NA |
|  | TTTTTTCCACCCTTTCAGATCATCTATGATCTTG | NR | NA | NA | NA | NA | NA | NA |
|  | AATTTTCTAATTCTATAAGTTCATTAATTCCGAT | NR | NA | NA | NA | NA | NA | NA |
|  | TATACTATTTACATAATTTTTTATGTGTCTGTCTAC | NR | NA | NA | NA | NA | NA | NA |
|  | TAATAGTGTTGTTCTCTATTAAAAGATACAATCCTGT | NR | NA | NA | NA | NA | NA | NA |
|  | TAGAATGTTATTATCTAAGTGGTCGATGTATTCC | *Staphylococcus* phage Stab20 | 58.4 | 116 | 100 | 4e-06 | 97.06 | LR215718.1 |
|  |  | *Staphylococcus* phage philPLA-RODI | 58.4 | 58.4 | 100 | 4e-06 | 97.06 | KP027446.1 |
|  | TCATACTAGCACCCCACTCTCTACTGAACAAGTATCA | NR | NA | NA | NA | NA | NA | NA |
|  | CTTAAAATCTAATTGCATTGTTATCAATTCCTTTA | NR | NA | NA | NA | NA | NA | NA |
|  | TCTGTAATGTATTCATTTAATGTAATCATAATTTTTTC | NR | NA | NA | NA | NA | NA | NA |
|  | TAGACCATTTACCTCATTATATTTATAGTCTTTATTA | NR | NA | NA | NA | NA | NA | NA |
|  | TTTTCTTTAACTGTTTTTACTGCCCATTTAATAGT | NR | NA | NA | NA | NA | NA | NA |
|  | ATATTTCTTCCATGAATAACACCCTCCTTTTTTCTA | NR | NA | NA | NA | NA | NA | NA |
|  | AAGTTAACGGCATTACCTAATAAAAATATTTTAGG | NR | NA | NA | NA | NA | NA | NA |
|  | TCATCTTTCATGTCACTGATTAATTCATTTGTA | NR | NA | NA | NA | NA | NA | NA |
| NR: no matching sequence, NA: No applicated | | | | | | | | |
| Supplementary table C. Spacer sequence (SS) from each CRISPR-Cas system-bearing strain of *Staphylococcus aureus* associated with a mobile genetic element (MGE) known. | | | | | | | | |
| Strain | Spacer sequence | MGE | Max score | Total score | Coverage (%) | e-value | Identity (%) | Accession number |
|  | GGTAATAGTTGCTCAATAGGTAATAAAACGTCGGT | NR | NA | NA | NA | NA | NA | NA |
| KUH140087 | ACAGAAAATATGGGACGCTATTGTTGAGATCTTTAA | *Staphylococcus aureus* strain AR_0471plasmid unanmed 1 | 62.1 | 62.1 | 100 | 4e-07 | 97.22 | CP010952.1 |
|  |  | *Staphylococcus aureus* plasmid Pwbg762 | 62.1 | 62.1 | 100 | 4e-07 | 97.22 | GQ900475.1 |
|  | AGACGAATTCACTAAAACAGTTAAAAAGTCAGTTGATG | *Staphylococcus aureus* phage Vb_SauS-SAP27 | 71.3 | 71.3 | 100 | 8e-10 | 100 | MN904510.1 |
|  |  | *Staphylococcus aureus* phage StauST398-5 | 71.3 | 71.3 | 100 | 8e-10 | 100 | KC595279.1 |
|  |  | *Staphylococcus aureus* phage SA13 | 71.3 | 71.3 | 100 | 8e-10 | 100 | JX094501.1 |
|  |  | *Staphylococcus aureus* phage StaST398-1 | 71.3 | 71.3 | 100 | 8e-10 | 100 | JX013863.1 |
|  | AAAGAAATGAGACTAGATGAATTAATTAAGTGGGCA | *Staphylococcal* phageMR003 | 67.6 | 67.6 | 100 | 8e-09 | 100 | AP019522.1 |
|  |  | *Staphylococcus* phage StaST398-3 | 67.6 | 67.6 | 100 | 8E-09 | 100 | JQ973847.1 |
|  |  | *Staphylococcus* phage SAP40 | 65.8 | 65.8 | 97 | 3-e08 | 100 | MK801683.1 |
| NR: no matching sequence, NA: No applicated | | | | | | | | |
| Supplementary table C. Spacer sequence (SS) from CRISPR-Cas system-bearing strain of *Staphylococcus aureus* associated to mobile genetic element (MGE) known. | | | | | | | | |
| Strain | Spacer sequence | MGE | Max score | Total score | Coverage (%) | e-value | Identity (%) | Accession number |
|  |  | *Staphylococcus* phage SAP33 | 65.8 | 65.8 | 97 | 3-e08 | 100 | MK801682.1 |
|  |  | *Staphylococcus* phage P954 | 65.8 | 65.8 | 97 | 3e-08 | 100 | GQ398772.2 |
|  |  | Bacterióphage 92 | 65.8 | 65.8 | 97 | 3e-08 | 100 | AY954967.1 |
|  |  |  |  |  |  |  |  |  |
|  |  |  |  |  |  |  |  |  |
|  | ATAAACAAATTGAAAATATGATTAGAATGGATAAGCATTT | NR | NA | NA | NA | NA | NA | NA |
|  | GATATGGACGGTTACAAAAGCAGTAACTATTATAG | NR | NA | NA | NA | NA | NA | NA |
| JS395 | TAAACCCGTTCAATTCGTTATCTTTAAATTCTTG | NR | NA | NA | NA | NA | NA | NA |
|  | CAACTTCGTCATCTTTCATCATTTCTCTTACATCA | NR | NA | NA | NA | NA | NA | NA |
|  | TATTTCTTCCATGAATAACACCCTCCTTTTTTCTA | NR | NA | NA | NA | NA | NA | NA |
|  | AGTTAACGGCATTACCTAATAAAAATATTTTAGG | NR | NA | NA | NA | NA | NA | NA |
|  | CATCTTTCATGTCACTGATTAATTCATTTGTA | NR | NA | NA | NA | NA | NA | NA |
|  | GTAATAGTTGCTCAATAGGTAATAAAACGTCGGT | NR | NA | NA | NA | NA | NA | NA |
| AR 0472 | ACCGACGTTTTATTACCTATTGAGCAACTATTACC | NR | NA | NA | NA | NA | NA | NA |
|  | TACAAATGAATTAATCAGTGACATGAAAGATGA | NR | NA | NA | NA | NA | NA | NA |
|  | CCTAAAATATTTTTATTAGGTAATGCCGTTAACTT | NR | NA | NA | NA | NA | NA | NA |
| NR: no matching sequence, NA: No applicated | | | | | | | | |
| Supplementary table C. Spacer sequence (SS) from each CRISPR-Cas system-bearing strain of *Staphylococcus aureus* associated with a mobile genetic element (MGE) known. | | | | | | | | |
| Strain | Spacer sequence | MGE | Max score | Total score | Coverage (%) | e-value | Identity (%) | Accession number |
|  | TAGAAAAAAGGAGGGTGTTATTCATGGAAGAAATAT | NR | NA | NA | NA | NA | NA | NA |
|  | TGATGTAAGAGAAATGATGAAAGATGACGAAGTTGT | NR | NA | NA | NA | NA | NA | NA |
|  | CAAGAATTTAAAGATAACGAATTGAACGGGTTTAT | NR | NA | NA | NA | NA | NA | NA |
|  | ACAGGATTGTATCTTTTAATAGAGAACAACACTATTA | NR | NA | NA | NA | NA | NA | NA |
|  | GTAGACAGACACATAAAAAATTATGTAAATAGTATA | NR | NA | NA | NA | NA | NA | NA |
|  | ATCGGAATTAATGAACTTATAGAATTAGAAAATT | NR | NA | NA | NA | NA | NA | NA |
|  | CAAGATCATAGATGATCTGAAAGGGTGGAAAAAA | NR | NA | NA | NA | NA | NA | NA |
|  | AGATAATCTAGGTATCGGAATTAATGAACTTATAGA | NR | NA | NA | NA | NA | NA | NA |
|  | CAAAGTAGAACAATTAGCAGAAATGGCGAGAGAAGT | NR | NA | NA | NA | NA | NA | NA |
| AR 0470 | CTTCTCTCGCCATTTCTGCTAATTGTTCTACTTTG | NR | NA | NA | NA | NA | NA | NA |
|  | CTATAAGTTCATTAATTCCGATACCTAGATTATCT | NR | NA | NA | NA | NA | NA | NA |
|  | TTTTTCCACCCTTTCAGATCATCTATGATCTTG | NR | NA | NA | NA | NA | NA | NA |
|  | CTGTAATGTATTCATTTAATGTAATCATAATTTTTTC | NR | NA | NA | NA | NA | NA | NA |
|  | AGACCATTTACCTCATTATATTTATAGTCTTTATTA | NR | NA | NA | NA | NA | NA | NA |
|  | TTTCTTTAACTGTTTTTACTGCCCATTTAATAGT | NR | NA | NA | NA | NA | NA | NA |
|  | TAAACCCGTTCAATTCGTTATCTTTAAATTCTTG | NR | NA | NA | NA | NA | NA | NA |
|  | CAACTTCGTCATCTTTCATCATTTCTCTTACATCA | NR | NA | NA | NA | NA | NA | NA |
| NR: no matching sequence, NA: No applicated | | | | | | | | |
| Supplementary table C. Spacer sequence (SS) from each CRISPR-Cas system-bearing strain of *Staphylococcus aureus* associated with a mobile genetic element (MGE) known. | | | | | | | | |
| Strain | Spacer sequence | MGE | Max score | Total score | Coverage (%) | e-value | Identity (%) | Accession number |
|  | TATTTCTTCCATGAATAACACCCTCCTTTTTTCTA | NR | NA | NA | NA | NA | NA | NA |
|  | AGTTAACGGCATTACCTAATAAAAATATTTTAGG | NR | NA | NA | NA | NA | NA | NA |
|  | CATCTTTCATGTCACTGATTAATTCATTTGTA | NR | NA | NA | NA | NA | NA | NA |
|  | GTAATAGTTGCTCAATAGGTAATAAAACGTCGGT | NR | NA | NA | NA | NA | NA | NA |
| AR 0473 | ACCGACGTTTTATTACCTATTGAGCAACTATTACC | NR | NA | NA | NA | NA | NA | NA |
|  | TACAAATGAATTAATCAGTGACATGAAAGATGA | NR | NA | NA | NA | NA | NA | NA |
|  | CCTAAAATATTTTTATTAGGTAATGCCGTTAACTT | NR | NA | NA | NA | NA | NA | NA |
|  | TAGAAAAAAGGAGGGTGTTATTCATGGAAGAAATAT | NR | NA | NA | NA | NA | NA | NA |
|  | TGATGTAAGAGAAATGATGAAAGATGACGAAGTTGT | NR | NA | NA | NA | NA | NA | NA |
|  | CAAGAATTTAAAGATAACGAATTGAACGGGTTTAT | NR | NA | NA | NA | NA | NA | NA |
|  | ACAGGATTGTATCTTTTAATAGAGAACAACACTATTA | NR | NA | NA | NA | NA | NA | NA |
|  | GTAGACAGACACATAAAAAATTATGTAAATAGTATA | NR | NA | NA | NA | NA | NA | NA |
|  | ATCGGAATTAATGAACTTATAGAATTAGAAAATT | NR | NA | NA | NA | NA | NA | NA |
|  | CAAGATCATAGATGATCTGAAAGGGTGGAAAAAA | NR | NA | NA | NA | NA | NA | NA |
|  | AGATAATCTAGGTATCGGAATTAATGAACTTATAGA | NR | NA | NA | NA | NA | NA | NA |
|  | CAAAGTAGAACAATTAGCAGAAATGGCGAGAGAAGT | NR | NA | NA | NA | NA | NA | NA |
| NR: no matching sequence, NA: No applicated | | | | | | | | |
